# Novel 4,8-benzobisthiazole copolymers and their field-effect transistor and photovoltaic applications†

**DOI:** 10.1039/c7tc03959j

**Published:** 2017-11-13

**Authors:** Gary Conboy, Rupert G. D. Taylor, Neil J. Findlay, Alexander L. Kanibolotsky, Anto R. Inigo, Sanjay S. Ghosh, Bernd Ebenhoch, Lethy Krishnan Jagadamma, Gopala Krishna V. V. Thalluri, Muhammad T. Sajjad, Ifor D. W. Samuel, Peter J. Skabara

**Affiliations:** a WestCHEM, Department of Pure and Applied Chemistry, Thomas Graham Building, University of Strathclyde Glasgow G1 1XL UK peter.skabara@glasgow.ac.uk; b Institute of Physical-Organic Chemistry and Coal Chemistry 02160 Kyiv Ukraine; c Organic Semiconductor Centre, SUPA, School of Physics and Astronomy, University of St. Andrews, North Haugh, St. Andrews KY16 9SS UK

## Abstract

A series of copolymers containing the benzo[1,2-*d*:4,5-*d*′]bis(thiazole) (BBT) unit has been designed and synthesised with bisthienyl-diketopyrrolopyrrole (DPP), dithienopyrrole (DTP), benzothiadiazole (BT), benzodithiophene (BDT) or 4,4′-dialkoxybithiazole (BTz) comonomers. The resulting polymers possess a conjugation pathway that is orthogonal to the more usual substitution pathway through the 2,6-positions of the BBT unit, facilitating intramolecular non-covalent interactions between strategically placed heteroatoms of neighbouring monomer units. Such interactions enable a control over the degree of planarity through altering their number and strength, in turn allowing for tuning of the band gap. The resulting 4,8-BBT materials gave enhanced mobility in p-type organic field-effect transistors of up to 2.16 × 10^−2^ cm^2^ V^−1^ s^−1^ for pDPP2ThBBT and good solar cell performance of up to 4.45% power conversion efficiency for pBT2ThBBT.

## Abstract 2

A series of BBT-containing copolymers featuring the 4,8-conjugation pathway is presented and evaluated.

## Graphical abstract

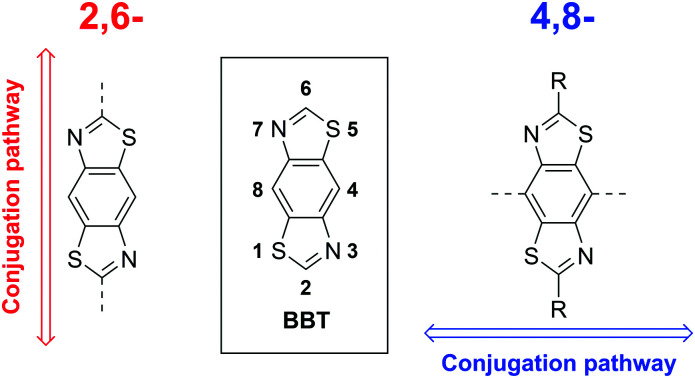

## Introduction

The use of organic materials as the active components of electronic devices has been an area of much research over the past few decades. Such interest over inorganic materials (*e.g.* silicon based), is largely due to their inherent tunability, solubility and flexibility, allowing for devices to be made from organic materials with finely tuned properties that can be processed from solution onto flexible substrates.^1–3^ The development of new materials for use in organic field-effect transistors (OFETs) is facilitating great advancements in charge carrier mobility, with these materials finding many applications in modern technology such as biosensors,^4^ addressing in active matrix light-emitting diodes (AMOLEDs)^5^ and as a low cost, flexible alternative to amorphous silicon in radiofrequency identification tagging (RFIDs).^6^ In order for organic materials to find useful applications, the mobility should be at least comparable to amorphous silicon, which has a hole mobility (*μ*) of 0.1 ≤ *μ* ≤ 1 cm^2^ V^−1^ s^−1^.^7^ There are now many reports of organic materials that have values *μ* > 10 cm^2^ V^−1^ s^−1^.^8–15^ Many of these leading candidates currently require pre-treatment of the SiO_2_ substrate to control polymer chain self-assembly^12^ or increase wettability,^2^ and/or thermal annealing at high temperature^16^ in order to achieve a suitable morphology capable of affording high mobility.^17^ Such processes add to the time, cost and complexity of fabrication and, as such, materials which show high mobility with minimal processing steps are highly desired for industrial use.

Organic semiconductors are also often studied for use as functional active layer materials in organic photovoltaic (OPV) devices, as they can be engineered to have a broad absorption across the solar spectrum. When combined with a suitable acceptor species, resultant organic solar cells can have power conversion efficiencies (PCEs) of over 12%.^18,19^ In order to minimise the band gap, as well as to increase the carrier mobility and self-assembly within the bulk phase, it is beneficial to place alternating conjugated donor and acceptor units into the backbone of the conductive material to facilitate a push–pull effect.^20,21^ Such a structure is readily achievable in polymeric form through the copolymerisation of suitably functionalised donor and acceptor units. Many of these units contain multiple heteroatoms that offer further advantages, such as planarisation and strong interchain packing due to a combination of π–π stacking and non-covalent heteroatom/weak hydrogen bonding interactions in the bulk material.^22–25^ For example, the use of thiazole (rather than thiophene) facilitates these advantages in combination with deeper highest occupied molecular orbital (HOMO) and lowest unoccupied molecular orbital (LUMO) energy levels^26^ to give increased device performance in both OFETs^27,28^ and OPVs.^29,30^ Additionally, intermolecular non-covalent interactions in the bulk are evident, further contributing to improved charge carrier properties.^26,31–33^

**Fig. 1:**
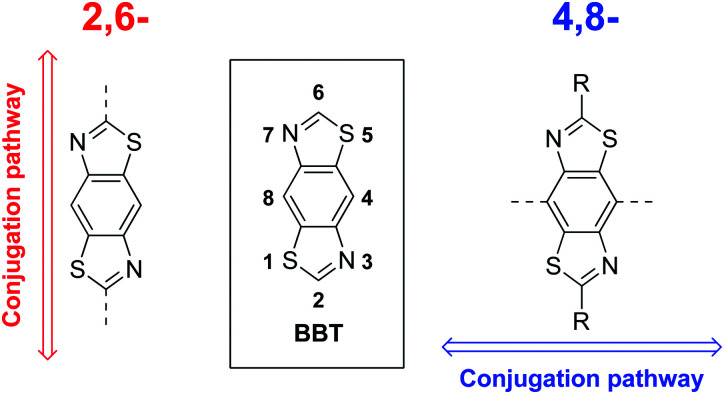
Typical 2,6-BBT conjugation pathway (left) and alternate 4,8-BBT conjugation pathway (right).

**Fig. 2:**
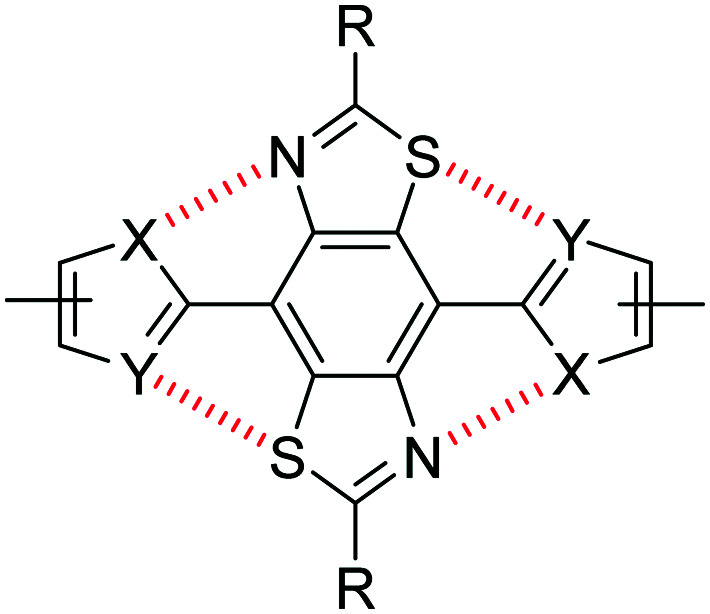
Non-covalent intramolecular interactions between S and N atoms of a BBT core and flanking heterocycles.

Recently we highlighted the use of a thiazole-containing benzobisthiazole (BBT) unit with an orthogonal (4,8- *vs.* traditional 2,6-) conjugation pathway (Fig. 1).^25^ Whilst BBT units possessing the 2,6-conjugation pathway are common and well-studied, those with 4,8-substitution have been underexplored in comparison,^25,34–38^ despite offering a better template with which to facilitate planarising intramolecular non-covalent interactions with neighbouring heterocycles.^25^ By carefully selecting the type and location of heteroatoms in the flanking heterocycles (Fig. 2), the planarity of the resultant molecule or polymer can be tuned and, in turn, their solubility and energy gap can be modified.

Non-covalent interactions are defined as contact distances between two atoms (often heteroatoms) which are shorter than the sum of the van der Waals radii of the two corresponding atoms. Through a combination of X-ray crystallography and computational simulations we have previously demonstrated that the inclusion of thiophene units either side of the BBT heterocycle (Fig. 2: X = CH, Y = S) gives a twisted, non-rigid structure. In the orientation depicted in Fig. 2, C–H⋯N hydrogen bonding interactions are offset by the repulsive S⋯S interactions, whilst S⋯N interactions (when the flanking heterocycles are flipped 180° relative to Fig. 2) are deterred by steric hindrance between the C–H and S groups.^25^ Conversely, utilising thiazole moieties in place of thiophene (Fig. 2: X = S, Y = N) results in four intramolecular S⋯N non-covalent interactions and a highly planarised structure (maximum torsion angle of 5.1° across the C–C bond connecting the BBT unit and heterocycle), and the use of furan (Fig. 2: X = CH, Y = O) leads to a similarly highly planarised structure (maximum torsion angle of 4.1°) through non-covalent S⋯O interactions.^25^ These, and other results, have shown that non-covalent S⋯N and S⋯O contacts offer favourable interactions of comparable strength, whilst S⋯S interactions are repulsive and N⋯O interactions are weak/negligible. It is important to note that additional intramolecular hydrogen bonding interactions with the BBT nitrogen atoms may be playing a role when X = CH (Fig. 2), however it has been unequivocally shown that heteroatom-heteroatom interactions are influential on the structure of such molecules.^25^

In this work we describe the synthesis of a series of BBT copolymers conjugated along the 4,8-substitution pathway (Fig. 1) and report their semiconducting properties in OFET and OPV devices. Bisthienyl-diketopyrrolopyrrole (DPP), dithienopyrrole (DTP), benzothiadiazole (BT), benzodithiophene (BDT) and 4,4′-dialkoxybithiazole (BTz) were selected as comonomers due to their reported behaviour in high performance OFET^32,39^ and OPV^40^ devices, but also to allow for band gap variation through HOMO and LUMO energy level tuning and to provide varying degrees of planarity through non-covalent heteroatom interactions (as discussed above).

## Synthesis

**Scheme 1:**
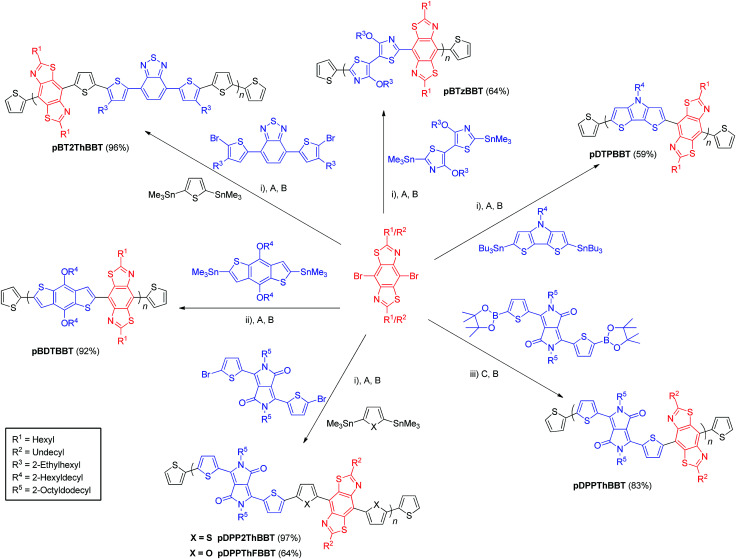
Synthesis of BBT-containing copolymers. Yields calculated from repeat units as drawn. *Reagents and conditions*: (i) Pd_2_(dba)_3_, P(*o*-tol)_3_, chlorobenzene, 160 °C, μW. (ii) Pd_2_(dba)_3_, P(*o*-tol)_3_, toluene, reflux. (iii) Pd_2_(dba)_3_, P(*o*-tol)_3_, K_3_PO_4_, THF, reflux. *End-capping reagents*: (A) tributyl(thiophen-2-yl)stannane. (B) 2-Bromothiophene. (C) Thiophen-2-ylboronic acid.

The targeted BBT-containing polymers were realised *via* Stille or Suzuki cross-coupling mediated polymerisations between alkylated 4,8-dibromo-BBT and suitably functionalised bisthienyl-diketopyrrolopyrrole (DPP),^41,42^ dithienopyrrole (DTP), benzothiadiazole (BT),^43^ benzodithiophene (BDT)^44^ or 4,4′-dialkoxybithiazole (BTz)^32^ monomers. Each polymer was then end-capped with thiophene units *via* subsequent Stille or Suzuki cross-coupling reactions with commercial tributyl(thiophen-2-yl)stannane (or thiophen-2-ylboronic acid in the case of pDPPThBBT) then 2-bromothiophene. Polymers containing a biheterocyclic bridge between donor/acceptor units were prepared as random copolymers using 2,5-bis(trimethylstannyl)thiophene^45^ (pDPP2ThBBT and pBT2ThBBT) or 2,5-bis(trimethylstannyl)furan^45^ (pDPPThFBBT) as a third monomer. As a result, their structures shown in Scheme 1 represent an idealised structure in each case. Detailed synthetic procedures for all monomers and polymers can be found in the ESI.†

Molecular weights (Table 1) of the resultant polymers were determined by gel permeation chromatography (GPC) in chloroform or *o*-dichlorobenzene solution and show a range of different values (14.4–96.0 kg mol^−1^). The limiting factor in molecular weight for each polymer is solubility – all polymers precipitated from solution during their respective polymerisations. Despite their very different molecular weights, pDPP2ThBBT and pDPPThFBBT have very similar solubility, suggesting that there is reduced rotational freedom in pDPPThFBBT due to intramolecular S⋯O and C–H⋯N interactions,^25^ facilitated by the furan rings flanking the BBT unit. In contrast, the thiophene moieties flanking the BBT unit in pDPP2ThBBT result in unfavourable S⋯S interactions and hence a more twisted structure, allowing the growing polymer chain to remain in solution longer before precipitating. Molecular weight variation between pDPPThBBT, pBT2ThBBT and pBDTBBT (which all feature thiophene units flanking the BBT units and hence contain unfavourable S⋯S interactions) is likely due to a combination of different alkyl chain lengths (leading to different solubility limits of the growing polymer chains during polymerisation) and the variety of polymerisation conditions used across the series.

**Table 1:** GPC, optical, electrochemical and thermal decomposition (*T*_d_) data for the 4,8-BBT copolymers. GPC data not obtained for pBTzBBT and pDTPBBT due to incomplete polymer solubility

Polymer	*M*_w_ (kg mol^−1^)	PDI	*E* ^opt^_g_ c (eV)	*E* ^elect^_g_ d (eV)	Solution^e^		Film		HOMO^d^ (eV)	LUMO^d^ (eV)	*T*_d_ (°C)
					*λ*_max_ (nm)	*λ*_onset_ (nm)	*λ*_max_ (nm)	*λ*_onset_ (nm)			
pBTzBBT	—	—	1.53	1.69	685, 740^f^	795	657, 731^f^	808	−4.69	−3.00	360
pDPPThBBT	14.4^a^	1.70	1.36	1.39	790	875	728	910	−4.90	−3.51	395
pDPP2ThBBT	96.0^a^	2.75	1.39	1.43	765	865	748	895	−5.10	−3.67	411
pDPPThFBBT	18.0^a^	2.04	1.43	1.26	716^f^, 751	855	705, 756	870	−4.86	−3.60	365
pBT2ThBBT	17^a^	1.80	1.58	—	548	690	620	785	−5.20	—	451
pBDTBBT	61^b^	1.90	2.00	2.24	493	555	567	620	−5.29	−3.05	332
pDTPBBT	—	—	1.84	2.11	530	620	536	675	−4.80	−2.69	401

a Calculated from GPC using 0.5 mg ml^−1^ solutions in chlorobenzene at 80 °C.

b Calculated from GPC using 1 mg ml^−1^ solutions in chloroform at 22 °C.

c Calculated from the onset of the longest solid state wavelength absorption peak.

d Found from CV, using the onset of redox activity and referenced to Fc/Fc^+^ (−4.8 eV).

e Absorption spectra obtained from *o*-dichlorobenzene solutions.

f Shoulder.

## Optical properties

**Fig. 3:**
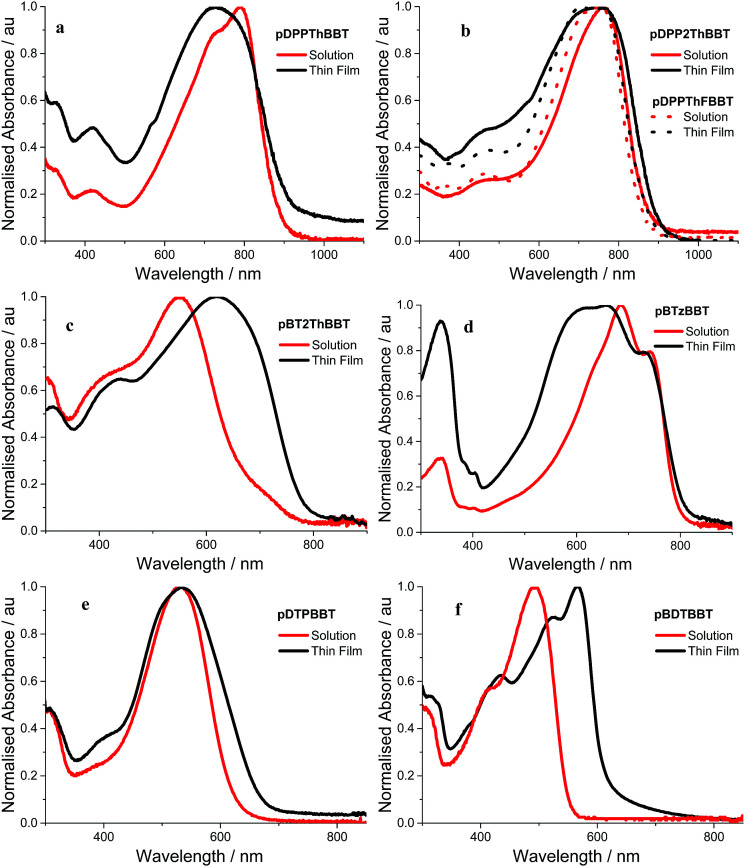
Absorption spectra of (a) pDPPThBBT, (b) pDPP2ThBBT/pDPPThFBBT, (c) pBT2ThBBT, (d) pBTzBBT, (e) pDTPBBT and (f) pBDTBBT in *o*-dichlorobenzene solution (red) and as thin films drop cast from *o*-dichlorobenzene (a–e) or chloroform (f) (black).

Absorption spectra of the 4,8-BBT copolymers were obtained in *o*-dichlorobenzene solution and as thin films. The resulting spectra (Fig. 3a–f) all show broad absorption, extending up to 900 nm and resulting in optical band gaps in the range 1.36–2.00 eV (as calculated from the onset of the longest wavelength solid state absorption peak). All of the 4,8-BBT copolymers show a red shift in their longest wavelength absorbance onset when moving from solutions to thin films due to increased intermolecular interactions. However, this red shift is very small (≤35 nm) for the three DPP containing copolymers (pDDPThBBT, pDDP2ThBBT and pDPPThFBBT) and pBTzBBT, indicating that these materials possess a rigid, aggregated structure even in solution. In contrast, pBT2ThBBT, pDTPBBT and pBDTBBT show a more significant red shift (up to 95 nm) in their film form, suggesting enhanced order due to aggregation and molecular packing in the solid state. This is in part due to these polymers possessing thiophene groups flanking the BBT unit, allowing for a more twisted and hence less conjugated structure in solution.

Comparison of the optical band gaps reveals that the DPP containing polymers (pDDPThBBT, pDDP2ThBBT and pDPPThFBBT) have very similar optical properties, and the smallest optical band gaps (1.36–1.43 eV) of all the 4,8-BBT copolymers. This suggests that the strong electron-accepting nature of the DPP unit causes it to dominate the optical properties of these copolymers, resulting in red-shifted absorption. Copolymers featuring weaker acceptor units (BTz and BT) show slightly wider optical band gaps (1.53 and 1.58 eV for pBTzBBT and pBT2ThBBT, respectively), whilst those containing electron donating units have the widest optical band gaps (1.84 and 2.00 eV for pDTPBBT and pBDTBBT, respectively).

**Fig. 4:**
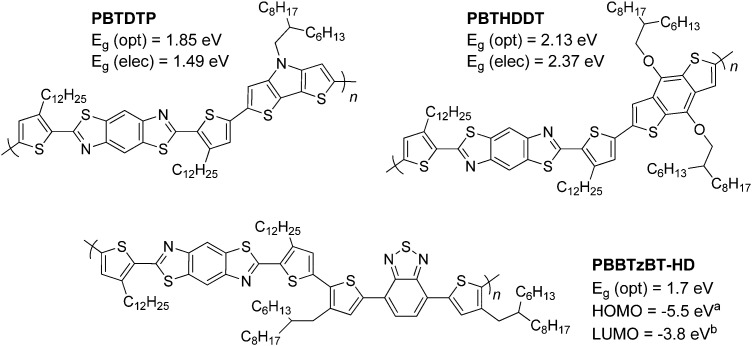
Previously reported 2,6-BBT copolymers featuring DTP (top left),^47^ BDT (top right)^47^ and BT (bottom) units.^46^^*a*^calculated from photoelectron yield spectroscopy, ^*b*^calculated using (*E*_g_ (opt) + *E*_HOMO_).

The optical properties of pBT2ThBBT, pBDTBBT and pDTPBBT reveal comparable absorption profiles to their equivalent 2,6-BBT copolymers (PBBTzBT-HD,^46^PBTHDDT^47^ and PBTDTP,^47^ respectively, Fig. 4) when measured in solution, with either similar or slightly hypsochromically shifted absorption onsets. As thin films, the absorption properties of pDTPBBT and its 2,6-substituted counterpart (PBTDTP)^47^ are also similar, and result in essentially identical optical band gaps (1.84 and 1.85 eV, respectively). However, the solid state absorption profiles of pBT2ThBBT and pBDTBBT are bathochromically shifted compared to their 2,6-analogues, resulting in lower optical band gaps (1.58 *vs.* 1.7 eV^46^ for the BT-containing polymers and 2.00 *vs.* 2.13 eV^47^ for the BDT-containing polymers). Moreover, the absorption window of pBT2ThBBT is significantly broader than that of its 2,6-analogue (PBBTzBT-HD),^46^ allowing for increased photon absorption.

## Electrochemical properties

To establish their electronic characteristics, cyclic voltammetry (CV) was performed on solutions and thin films (cast on platinum disc electrodes) of the 4,8-BBT copolymers. Electronic band gaps (Table 1) were found to be between 1.26–2.24 eV and broadly consistent with the trend in optical values. Cyclic voltammograms for all 4,8-BBT copolymers in solution and solid state are shown in Fig. S8–S20 (ESI†), however pBT2ThBBT did not reveal a reduction peak at potentials as low as −2.0 V, preventing electrochemical determination of the polymer's LUMO level.

pDPPThBBT and pDPP2ThBBT exhibit relatively similar HOMO and LUMO energy levels, and by extension closely matching electrochemical band gaps (1.39 and 1.43 eV, respectively). pDPPThFBBT shows a slightly lower electrochemical band gap of 1.26 eV, which is likely due to a combination of the lower resonance stabilisation energy of the furan groups flanking the BBT unit and increased conjugation through planarising intramolecular S–O interactions. The electrochemical data of the DPP-containing 4,8-BBT copolymers is consistent with the optical data in demonstrating that the strongly electron-accepting nature of the DPP unit results in it dominating the materials electronic behaviour, although thiophene or furan flanking units can fine tune this behaviour further.

In agreement with the optical data and comparing to those copolymers containing DPP, copolymers featuring weaker electron acceptor units have slightly wider electrochemical band gaps (1.69 eV for pBTzBBT), whilst those featuring electron-donating units (pDTPBBT and pBDTBBT) have much wider electrochemical band gaps (2.11 and 2.24 eV, respectively). In comparison to its 2,6-analogue, pBDTBBT has a smaller electrochemical band gap (2.24 eV *vs.* 2.37 eV for PBTHDDT),^47^ which is in agreement to the bathochromic shift seen in the absorption measurements.

## Organic field-effect transistor (OFET) fabrication

Bottom gate/bottom contact (BGBC) OFETs were prepared from commercially available (Fraunhofer Institute für Photonische Mikrosysteme IPMS, Dresden) n-doped silicon substrates with a 200 nm layer of thermally grown SiO_2_ dielectric and prefabricated interdigitated gold source/drain electrodes (channel lengths: 2.5, 5, 10 and 20 μm, channel width: 1 cm). Unless otherwise stated, solutions were pre-stirred at 50 °C for at least three hours, then spin-coated whilst hot at 2000 rpm. Following any required annealing, the devices were dried under vacuum (5 × 10^−2^ mbar), then their performance was measured on a Keithley 4200 semiconductor characterisation system, all whilst in a dry, nitrogen-filled atmosphere. Mobilities were calculated in the saturation region *via* the standard method using the following equation:
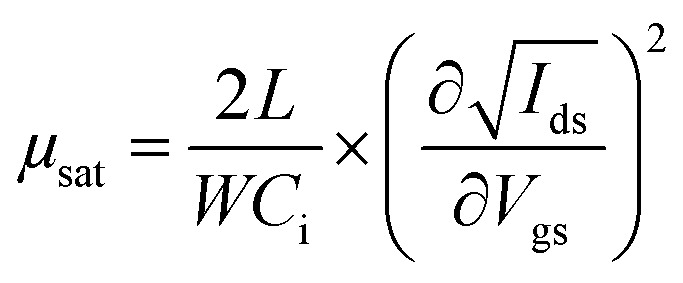
where *I*_ds_ is the source–drain current, *μ* is the carrier mobility, *V*_gs_ is the gate voltage, *L* is the channel length, *W* is the channel width and *C*_i_ is the capacitance per unit area of the insulator material.

**Fig. 5:**
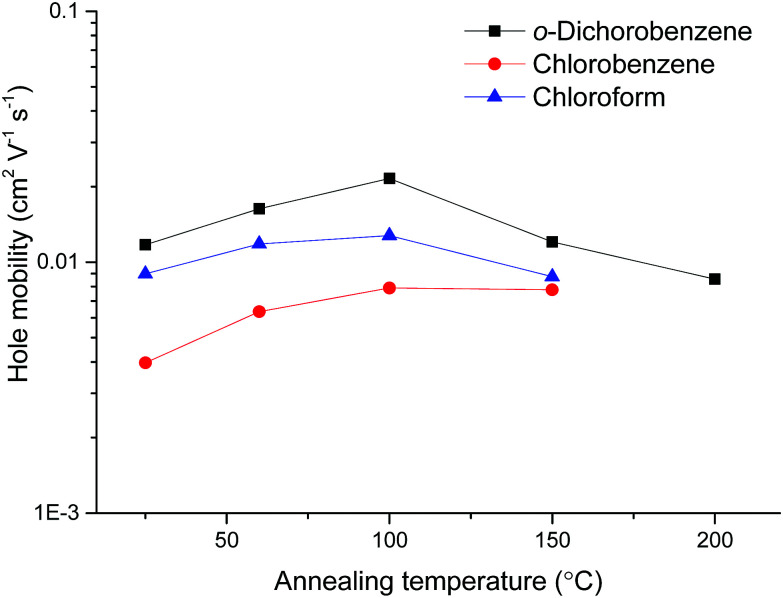
Hole mobilities of OFETs based on pDPP2ThBBT as a function of annealing temperature.

Device optimisation studies were carried out for pDPP2ThBBT (selected for its solubility and relatively high molecular weight, which has been shown to result in improved charge carrier mobility)^48,49^ through variation of the annealing temperature (*as cast*, 60, 100, 150 and 200 °C) and solvent (chloroform, chlorobenzene or *o*-dichlorobenzene). Solutions of 10 mg ml^−1^ concentration were prepared and deposited onto the prefabricated substrates in accordance with the previously stated procedure, with the same device tested at each annealing temperature increment. To facilitate accurate comparison of the materials and limit further processing steps, the use of self-assembled monolayers (*e.g.* pentafluorobenzenethiol)^50^ or processing additives were avoided. Summarised data for devices based on pDPP2ThBBT are shown in Table 2 and Fig. 5, with full device data in Fig. S21–S33 (ESI†).

**Table 2:** Device characteristics for pDPP2ThBBT based OFETs, averaged across 3 devices

Solvent	Annealing temperature (°C)	*μ*_h_ (cm^2^ V^−1^ s^−1^)	*I*_on_/*I*_off_	*V*_th_ (V)
*o*-Dichlorobenzene	As cast	1.17 × 10^−2^	10^3^	12
	60	1.63 × 10^−2^	10^3^	14
	100	2.16 × 10^−2^	10^3^	5
	150	1.20 × 10^−2^	10^3^	0
	200	8.56 × 10^−3^	10^2^	−6

Chlorobenzene	As cast	3.97 × 10^−3^	10^3^	13
	60	6.35 × 10^−3^	10^3^	14
	100	7.89 × 10^−3^	10^3^	6
	150	7.76 × 10^−3^	10^3^	1
	200^a^	—	—	—

Chloroform	As cast	8.98 × 10^−3^	10^2^	12
	60	1.18 × 10^−2^	10^3^	11
	100	1.28 × 10^−2^	10^3^	1
	150	8.76 × 10^−3^	10^3^	−1
	200^a^	—	—	—

a Devices gave no response due to poor film morphology.

Fig. 5 shows that *o*-dichlorobenzene is the best solvent for preparing OFETs from pDPP2ThBBT, with the devices outperforming analogous devices prepared from chlorobenzene or chloroform solutions at all annealing temperatures. However, annealing was only beneficial up to 100 °C, after which point further annealing resulted in a drop-off in device performance due to visibly poor film morphology. This is likely a result of the polymer exhibiting significant rigidity, meaning that modest annealing temperatures are enough to force the *as cast* film towards the thermodynamically most stable (crystalline) state resulting in grain boundaries. Accordingly, OFETs made from other 4,8-BBT copolymers were processed from *o*-dichlorobenzene solution and annealed at 100 °C; data obtained from these OFETs are shown in Table 3, with output and transfer characteristics in Fig. S34–S39 (ESI†).

**Table 3:** OFET device data for the 4,8-BBT copolymers. Device parameters: BGBC, spin coated from 10 mg ml^−1^*o*-dichlorobenzene solution, annealed at 100 °C. Averaged across 3 devices

Polymer	*μ*_h_ (cm^2^ V^−1^ s^−1^)	*I*_on_/*I*_off_	*V*_th_ (V)
pBTzBBT	2.16 × 10^−3^	10^5^	−2
pDPPThBBT	3.23 × 10^−6^	10^5^	−10
pDPP2ThBBT	2.16 × 10^−2^	10^3^	5
pDPPThFBBT	2.03 × 10^−3^	10^5^	−2
pBT2ThBBT	3.60 × 10^−3^	10^3^	−5
pBDTBBT	8.69 × 10^−5^	10^3^	−2
pDTPBBT	2.11 × 10^−5^	10^3^	11

**Fig. 6:**
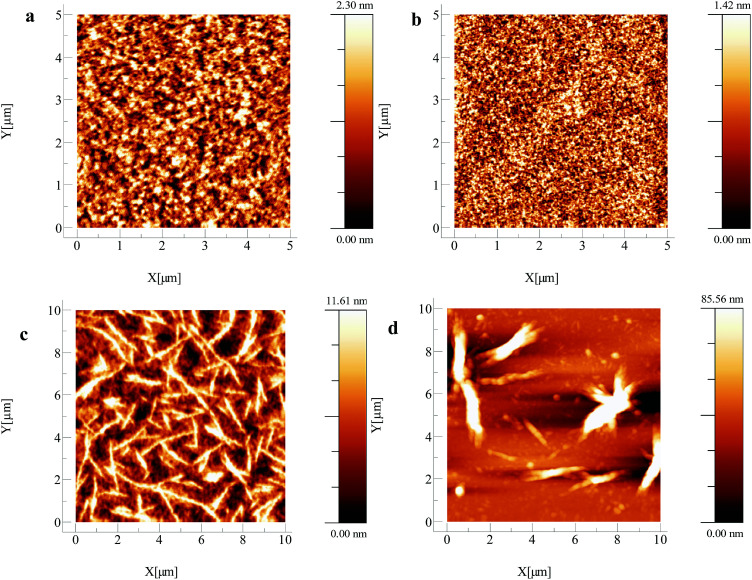
AFM images of OFETs after annealing at 100 °C fabricated using (a) pDPP2ThBBT (RMS roughness = 0.55 nm), (b) pDDPThFBBT (RMS roughness = 0.34 nm), (c) pDPPThBBT (RMS roughness = 2.82 nm) and (d) pBTzBBT (RMS roughness = 15.63 nm).

The hole mobility values obtained show a large variation, spanning four orders of magnitude. pDPP2ThBBT based OFETs gave the highest hole mobility (2.16 × 10^−2^ cm^2^ V^−1^ s^−1^) in combination with a moderate *I*_on_/*I*_off_ ratio (10^3^) and low threshold voltage (5 V) for a p-type device, but suffer from increased hysteresis in both output and transfer characteristics (Fig. S21–S33, ESI†). This could be indicative of charge trapping in the bulk film or non-optimal device structure. pDPPThFBBT exhibits a slightly lower hole mobility (2.03 × 10^−3^ cm^2^ V^−1^ s^−1^), possibly due to its lower molecular weight^48,49^ and larger threshold voltage for p-type devices (−2 V). This is likely due to a non-ohmic contact between the gold electrode (work function −5.0 to −5.1 eV) and the shallow HOMO of pDPPThFBBT (−4.86 eV) resulting in poor charge injection. AFM images of the thin films of pDPP2ThBBT and pDPPThFBBT cast on OFET substrates (Fig. 6a and b) show very smooth uniform films with a root mean square (RMS) roughness of 0.55 and 0.34 nm, respectively, and grain boundaries on the order of 0.1 μm or less. Similarly acquired images of pDPPThBBT (Fig. 6c) show a much rougher film (RMS roughness of 2.82 nm) with domains extending up to 5 μm, and grain boundaries greater than 1 μm wide. This poor film morphology combined with the relatively low molecular weight of pDPPThBBT is likely the cause of the significantly inferior hole mobility (3.23 × 10^−6^ cm^2^ V^−1^ s^−1^).^49^

To overcome the poor solubility of pBTzBBT, all solutions used for OFET fabrication were pre-stirred at 100 °C for three hours then spin-coated whilst hot to prevent precipitation of the material. In spite of this, pBTzBBT produced the most *ideal* OFETs of those presented in this work, with modest hole mobility (2.16 × 10^−3^ cm^2^ V^−1^ s^−1^), but crucially a high *I*_on_/*I*_off_ ratio (10^5^) and a low driving voltage (−2 V). AFM (Fig. 6d) revealed that the film morphology of pBTzBBT was very rough (RMS roughness = 15.63 nm), which is likely due to the poor solubility of the polymer (despite the extra pre-treatment), meaning that further optimisation of the device preparation, or increasing the solubility of the polymer through the use of longer alkyl chains, could lead to a higher-performing solution-processed OFET.

An additional example of the impact of the BBT unit on OFET performance is evident in pBT2ThBBT. OFETs featuring pBT2ThBBT showed modest mobility (3.60 × 10^−3^ cm^2^ V^−1^ s^−1^), but this is over two orders of magnitude higher than a literature example featuring only BT units,^51^ highlighting the enhanced crystallinity afforded by incorporation of BBT units into the polymer backbone through increased potential for non-covalent interactions and 2D conjugation.

## Organic photovoltaic (OPV) devices

Due to the absorption characteristics in the visible spectral range, narrow band gap and efficient charge carrier transport properties of many of these 4,8-BBT copolymers, they were investigated for use as donor molecules in bulk heterojunction (BHJ) OPV devices. Unfortunately, due to the very poor solubility of pBTzBBT, blend solutions with fullerene acceptors of concentrations suitable for OPV devices could not be obtained. The remaining materials were employed as donor materials, blended with [6,6]-phenyl-C_71_-butyric acid methyl ester (PC_71_BM) and fabricated into OPV devices using the conventional architecture of ITO/PEDOT:PSS/BHJ/Ca/Al unless otherwise stated. The results are summarised in Table 4.

**Table 4:** OPV device data for the 4,8-BBT copolymers

Polymer	PCE avg. (%)	PCE best (%)	*V*_OC_ (V)	*J*_SC_ (mA cm^−2^)	FF (%)
pBTzBBT	—		—	—	—
pDPPThBBT	0.5 ± 0.03^a^	0.53^a^	0.62 ± 0.03^a^	1.60 ± 0.05^a^	50.2 ± 1.0^a^
pDPP2ThBBT	0.9 ± 0.3	1.2	0.6 ± 0.02	2.40 ± 0.50	60.0 ± 5.0
pDPPThFBBT	0.90 ± 0.02	0.92	0.64 ± 0.03	2.50 ± 0.12	55.0 ± 1.1
pBT2ThBBT	4.33 ± 0.12	4.45	0.65 ± 0.02	14.3 ± 0.39	48.0 ± 1.3
pBDTBBT	0.82 ± 0.02	0.84	0.81 ± 0.02	2.71 ± 0.05	39 ± 0.78
pDTPBBT	0.57 ± 0.04	0.62	0.69 ± 0.19	2.80 ± 0.06	30.8 ± 7.7

a Inverted architecture: ITO/Cs_2_CO_3_/BHJ/MoO_3_/Ag. The data are for the active layer blend ratios (donor/acceptor, w/w) giving the best and average (over 4 to 8 OPV devices) power conversion efficiency, namely pDPPThBBT : PC_71_BM, 1 : 3. pDPP2ThBBT : PC_71_BM, 1 : 2. pDPPThFBBT : PC_71_BM, 1 : 3. pBT2ThBBT : PC_71_BM, 1 : 1 (3% DIO). pBDTBBT : PC_71_BM, 1 : 1 (3% DIO). pDTPBBT : PC_71_BM, 1 : 1. Optimisation details of these devices are included in the ESI. The error bars (±) are standard deviations of the measured data.

pDPP2ThBBT and pDPPThFBBT exhibit very similar device performances across all measurements (PCEs of 0.89 and 0.87%, respectively) representing a small improvement over the simpler pDPPThBBT material (PCE 0.50%). The increased PCEs are attributed to the higher FF of 60% and 55% respectively, compared to 50% for pDPPThBBT. The FF obtained for each of the DPP-containing BBT copolymers increases proportionally with increasing hole mobility, which is in good agreement with previously published data.^52^

**Fig. 7:**
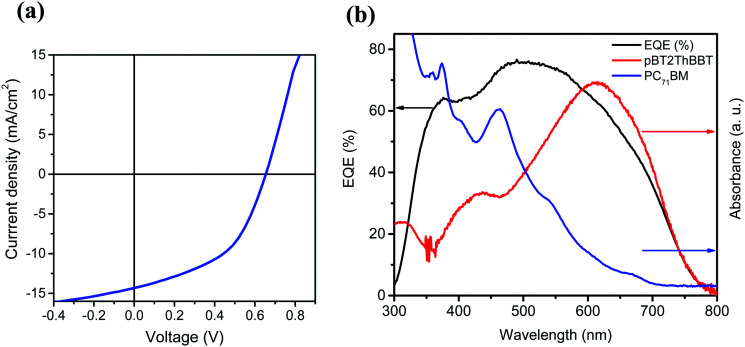
(a) *J*–*V* characteristics and (b) EQE spectra for the best performing pBT2ThBBT:PC_71_BM OPV devices.

The highest PCE (4.45%) was obtained from pBT2ThBBT using a 1 : 1 blend with PC_71_BM and 3% diiodooctane (DIO) as an additive. The high PCE can be largely attributed to a very high *J*_SC_ (14.32 mA cm^−2^) as well as moderate *V*_OC_ and FF (0.65 V, 48%). The impressive *J*_SC_ generated from this device, comparable to that of PTB7,^53^ indicates that this material is a promising candidate for high-efficiency OPV devices. The *J*–*V* characteristics, external quantum efficiency (EQE) and absorption spectra of the active layer blend components (pBT2ThBBT and PC_71_BM) corresponding to the best OPV device are shown in Fig. 7. The higher photocurrent of this polymer compared to others can be due to its broad absorption (as shown in Fig. 3) and the higher extinction coefficient (Fig. S44, ESI†) compared to other BBT polymers. Moreover, the exciton diffusion length 
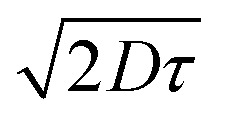
 of pBT2ThBBT determined by time resolved fluorescence studies is found to be ∼10 nm which is higher than many donor–acceptor polymers. For example, the reported value of 
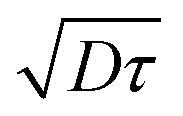
 for PTB7 of 4–5 nm^54^ would give 
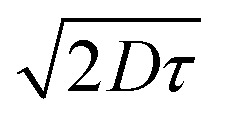
 of 6–7 nm. This enhanced exciton diffusion length of the pBT2ThBBT polymer can also contribute towards the increased photon harvesting of the pBT2ThBBT:PC_71_BM blend. The experimental details and calculations of exciton diffusion length are included in the ESI.†

In comparison to a 2,6-BBT analogue (PBBTzBT-DT),^46^pBT2ThBBT gives a higher base PCE (4.45 *vs.* 2.37%) using the same conventional device structure. However, PCEs of PBBTzBT-DT were shown to improve to 3.84%^46^ and 6.53%^55^ by utilising an inverted device structure and a ZnO electron transport layer respectively, suggesting further improvements in pBT2ThBBT based devices are possible.

## Summary

Copolymers of 4,8-benzobisthiazole (BBT) with bisthienyl-diketopyrrolopyrrole (DPP), dithienopyrrole (DTP), benzothiadiazole (BT), benzodithiophene (BDT) and 4,4′-dialkoxybithiazole (BTz) units have been synthesised for the first time. The resultant copolymers were found to possess optical band gaps (1.36–2.00 eV), equal to or narrower than their 2,6-BBT copolymer counterparts, with good agreement to their electrochemical band gaps (1.26–2.24 eV). The novel materials were fabricated into BGBC OFET devices resulting in record hole mobilities amongst similar 2,6-BBT or 4,8-BBT-containing polymers (up to 2.16 × 10^−2^ cm^2^ V^−1^ s^−1^ for pDPP2ThBBT). OFETs fabricated from pBTzBBT gave a highly optimal *I*_on_/*I*_off_ ratio (10^5^) and a low driving voltage (−2 V), despite poor film quality (established by AFM). Optimisation of this material/device structure is expected to result in efficient, low cost, solution-processed OFETs. OPV devices utilising the 4,8-BBT copolymers as donor materials with fullerene acceptors yielded (for the best material, pBT2ThBBT) a high short-circuit current of over 14 mA cm^−2^ and a respectable power conversion efficiency of 4.45%. Further improvements in device performance for pBT2ThBBT are expected to be realised from an inverted device structure and the use of a ZnO electron transport layer.^55^

## Conflicts of interest

There are no conflicts to declare.

## Supplementary Material

TC-005-C7TC03959J-s001
