# Supplementary material for: Novel 4,8-benzobisthiazole copolymers and their field-effect transistor and photovoltaic applications
Source: J Mater Chem C Mater. 2017 Nov 13;5(45):11927–36. doi: 10.1039/c7tc03959j (PMC13088948; doi:10.1039/c7tc03959j)
Supplement: TC-005-C7TC03959J-s001 [file TC-005-C7TC03959J-s001.pdf]

Electronic Supplementary Material (ESI) for Journal of Materials Chemistry C.
This journal is © The Royal Society of Chemistry 2017

Supporting information for: Novel 4,8-benzobisthiazole copolymers and their field-effect transistor and photovoltaic applications

Gary Conboy,[a] Rupert G. D. Taylor,[a] Neil J. Findlay,[a] Alexander L. Kanibolotsky,[a,b] Anto R. Inigo,[a] Sanjay S. Ghosh,[c] Bernd Ebenhoch,[c] Lethy K. Jagadamma,[c] Gopala Krishna V. V.Thalluri,[c] Muhammad T. Sajjad,[c] Ifor D. W. Samuel[c] and Peter J. Skabara[a]*

[a]WestCHEM, Department of Pure and Applied Chemistry, Thomas Graham Building, University of Strathclyde, Glasgow, G1 1XL, UK

[b]Institute of Physical-Organic Chemistry and Coal Chemistry, 02130 Kyiv, Ukraine

[c]Organic Semiconductor Centre, SUPA, School of Physics and Astronomy, University of St. Andrews, North Haugh, St. Andrews, KY16 9SS, UK

E-mail: peter.skabara@strath.ac.uk

General Remarks

Lithium diisopropylamide (LDA) was purchased from Sigma Aldrich and titrated against (+)-menthol with 2,2-bipyridyl as an indicator before use. *n*-BuLi was purchased from Sigma Aldrich and titrated against diphenylacetic acid before use. 2,2,6,6-Tetramethylpiperidine (TMP) was purchased from Alfa Aesar and freshly distilled from CaH before use. N-bromosuccinimide (NBS) was purchased from Sigma Aldrich and recrystallised from water, dried under vacuum and stored under Argon. $Pd(PPh_3)_4$ was synthesised prior to use and stored under Argon. Unless otherwise stated all other reagents were sourced commercially and used without further purification. Microwave syntheses were conducted using Biotage Initiator Classic microwave heating apparatus. Commercial TLC plates (Silica gel 60 F254) were used for thin-layer chromatography and column chromatography was performed on silica

gel Zeoprep 60 Hyd (40-63 μm mesh). Solvents were removed using a rotary evaporator (vacuum supplied by low vacuum pump) and, when necessary, a high vacuum pump was used to remove residual volatiles. Distillation of high boiling liquids was performed on a Kugelrohr Z24 with a high vacuum pump. Dry solvents were obtained from a solvent purification system using alumina as drying agent (SPS 400 from Innovative Technologies).

^{1}H and ^{13}C NMR spectra were recorded on either a Bruker AVIII 400 or Bruker AV400 at 400 MHz and 100 MHz respectively, or a Bruker DRX500 apparatus at 500 MHz and 125 MHz respectively. Chemical shifts are given in ppm; all *J* values are in Hz and samples are referenced to residual solvent peaks. Elemental analyses were obtained on a Perkin-Elmer 2400 analyser and MALDI-TOF spectra were run on a Shimadzu Axima-CFR spectrometer (mass range 1-150000 Da).

Thermogravimetric analysis (TGA) was performed on a Perkin Elmer Thermogravimetric Analyser (TGA7) under a constant flow of Ar (20 ml min^{-1}). Samples (approx. 5 mg) were placed on a standard platinum pan and loaded at 35°C to be analysed. The temperature was raised to 50°C followed by an isothermal period of 5 minutes. The temperature was raised again at a rate of 10°C min^{-1} until the desired temperature, at which point, the material was left for an isothermal period of 30 minutes. The percentage weight loss over time was recorded at this temperature and the data was processed using the Pyris Series Software.

UV-Vis-NIR spectroscopy was conducted on a Shimadzu 2600 spectrophotometer fitted with an integrating sphere and results normalised to λ_{max}.

CV measurements were performed on a CH Instruments 660A electrochemical workstation with *iR* compensation using anhydrous solvents (dichloromethane or acetonitrile). The electrodes were 8 mm platinum disk, platinum wire, and silver wire as the working, counter, and reference electrodes, respectively. All solutions were degassed (Ar) and contained monomer substrates in concentrations *ca.* 10^{-4} M, together with NBu_4PF_6 (0.1 M) as the supporting electrolyte. All measurements are displayed

relative to the $E_{1/2}$ of the Fc/Fc^+ redox couple. Thin-film CV measurements were conducted using material drop-cast from an appropriate solvent ($CHCl_3$ or chlorobenzene) onto the disk electrode and dried under low vacuum before use.

Gel permeation chromatography (GPC) was performed on an Agilent 1200 series GPC system with a column temperature of 80°C (chlorobenzene as eluent), or on a Viscotek GPC Max1000 system which includes a refractive index detector and two columns (KF-805L Shodex) at a flow rate of 1 ml min^{-1} (chloroform eluent).

Atomic force microscopy (AFM) experiments were performed on a Digital Instruments, Vecco metrology group, Dimension™ 3100, version 4.43B under ambient conditions.

Bottom gate/bottom contact OFETs were prepared using commercially available (Fraunhofer institute) n-doped silicon chips with 200 nm of thermally grown SiO_2 and prefabricated interdigitated Au fingers (30 nm thick S/D electrodes). Channel lengths were 2.5, 5, 10 and 20 μm, and the channel width was 1 cm. Polymer solutions (chloroform, chlorobenzene or *o*-dichlorobenzene, 10 mg ml^{-1}) were stirred at 50°C on a hot plate for at least 3 hours, then deposited by spin-coating the polymer at 2000 rpm in a nitrogen filled glovebox whilst hot. The films were annealed at elevated temperature (60, 100, 150 or 200°C) under N_2. The semiconductor performance was measured on a Keithley 4200 semiconductor characterisation system. Mobilities were calculated in the saturation region *via* the standard method using Equation 1 as follows:

$$\mu_{sat} = \frac{2L}{WC_i} \times \left(\frac{\partial\sqrt{I_{ds}}}{\partial V_{gs}}\right)^2$$

Equation 1- Calculation of saturation mobility

Where I_{ds} is the source drain current, μ is the carrier mobility, V_{gs} is the gate voltage, L is the channel length, W is the channel width and C_i is the capacitance per unit area of the insulator material.

Solar cells were fabricated on patterned ITO coated glass substrates (Xinyan Technology Ltd.) having sheet resistance of 10-13 ohm cm^{-2}. The substrates were cleaned by ultra-sonication in acetone and isopropanol successively for 10 mins each before drying in an oven at 100°C. The dried substrates were then treated with oxygen plasma for 3 mins before being coated with poly(3,4-ethylenedioxythiophene):poly(styrenesulfonate) (PEDOT:PSS) (CLEVIOS™ P VP AI 4083) *via* spin-coating at 4000 rpm to obtain films of ~30 nm thickness. The films were then annealed on a hot plate at 140°C for 10 mins to remove the residual moisture. The dried substrates were then transferred into a nitrogen glove box to deposit the active layer blend. The blend solution (in *o*-dichlorobenzene) was stirred at 40°C for at least 24 hrs prior to being spin coated at 800 rpm. 3% (v/v) diiodooctane (DIO) was added into the solution before spin coating as a solvent additive. The solution concentration and rpm were varied to provide films of different thicknesses. Calcium layer of thickness of 15 nm followed by a 100 nm aluminium layer were evaporated at a pressure of 2×10^{-6} mbar. Active areas of devices (2 x 4 mm^2) were defined by a shadow mask. The devices were then encapsulated by using UV-curable epoxy to glue a glass slide over the device. The current-voltage (*J-V*) characteristics were measured using a Keithley 2400 source measure unit and a Science-Tech, SS150 solar simulator under illumination at 100 mW cm^{-2} intensity. The light intensity at the test surface was calibrated using a Newport Oriel reference cell. For *J-V* measurement of the solar cells a shadow mask equal to the size of the test pixel was used.

Experimental

1,1'-(1,4-Phenylene)bis(thiourea)

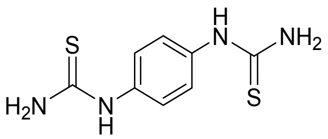

Benzene-1,4-diamine (17.0 g, 157 mmol), hydrochloric acid (30.7 mL, 125 mmol) and activated charcoal (1.1 g, 157 mmol) were added to a 500 mL round bottom flask then heated to 50 °C. The mixture was then filtered through a pad of celite, directly into another 500 mL flask. Ammonium

thiocyanate (48.4 g, 636 mmol) was then added and the reaction heated at 95 °C for 24 h with stirring. A yellow granular product precipitated during the reaction. The mixture was allowed to cool and the product was filtered and washed with hot water (2 x 40 mL). The yellow granular product was dried in a vacuum desiccator for 48 h and used without further purification (31.9 g, 90 %); m.p.: 280 °C (dec); ^{1}H NMR; δ_H (400 MHz DMSO-d^6): 10.08 (4H, s, NH_2), 7.80-8.04 (4H, m, Ar-H). The analysis was comparable with previously published data.[1]

*Benzo[1,2-d:4,5-d']bis(thiazole)-2,6-diamine (**1**):*

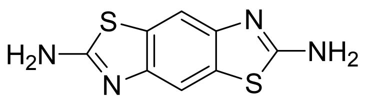

To a stirred suspension of 1,1'-(1,4-phenylene)bis(thiourea) (30 g, 133 mmol) in chloroform (80 mL) was added a solution of bromine (15.8 mL, 307 mmol) in chloroform (100 mL) slowly, as to prevent the reaction exceeding 50 °C. The orange slurry was stirred at rt. overnight. The reaction was then heated to reflux for 24 h. The reaction was allowed to cool before being filtered and washed with chloroform (4 x 300 mL). The product was then stirred with 20% sodium bisulfite solution at 90 °C until the orange colour subsided. The solid was filtered off when cold before being dissolved in boiling dilute HCl (1500 mL) and stirred with activated carbon for 3 h before being filtered through celite. Basification with ammonia precipitated a white amorphous solid. The solid was filtered, washed (3 x 100 mL methanol then 100 mL diethyl ether) and then dried in a vacuum desiccator (24.8 g, 84 %); m.p: >350 °C (>350 °C lit.); ^{1}H NMR; δ_H (400 MHz DMSO-d^6): 7.74 (2H, s, Ar-H), 7.43 (4H, s, NH_2).[2]

*2,5-Diaminobenzene-1,4-dithiol dihydrochloride (**2**):*

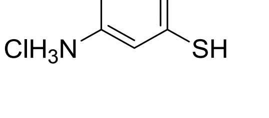

Benzo[1,2-*d*:4,5-*d'*]bis(thiazole)-2,6-diamine (12.0 g, 54.0 mmol) **(1)** was added to a 250 mL round bottomed flask and the flask degassed several times. Potassium hydroxide (48.5 g, 864 mmol) was dissolved in another round bottom flask in degassed water (60 mL) then transferred via syringe to the reaction flask. The mixture was stirred for 5 h under reflux then cooled overnight with stirring. The resulting mixture was filtered under Ar. The yellow precipitate was then dissolved in deaerated water (60 mL) and filtered directly into a flask containing deaerated water (120 mL) and concentrated hydrochloric acid (120 mL). The resulting white crystals were filtered, washed with degassed methanol and dried under Ar for 1 h before being used immediately in the next step without purification or exposure to air.

2,6-Dihexylbenzo[1,2-d:4,5-d']bis(thiazole) ***(3a)***

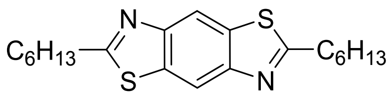

To an evacuated 250 mL 2-neck flask containing freshly synthesized **(2)** under Ar was added 1,2-dichlorobenzene (100 mL), heptanoyl chloride (18.9 mL, 122 mmol) and trimethylsilyl polyphosphate (33.7 mL, 294 mmol). The flask was heated to reflux for 48 h under Ar. Upon cooling, the solution was quenched with sat. $NaHCO_3$ solution and then extracted with dichloromethane (3 x 100 mL). The solution was then washed with sat. $NaHCO_3$ (3 x 200 mL) and brine (200 mL). The combined organics were then dried ($MgSO_4$) and the dichloromethane removed by rotary evaporation. The dichlorobenzene was distilled via Kughelrohr distillation and the resulting dark brown residue purified by silica gel column chromatography eluting with dichloromethane. The off-white solid was reprecipitated from dichloromethane/methanol and dried under vacuum to yield the *title compound* as a white solid (8.9 g, 46% over two steps); m.p.: 102 – 104 °C; ^{1}H NMR; δ_H (400 MHz $CDCl_3$): 8.38 (2H, s, Ar-H), 3.13 (4H, t, *J* 7.8 Hz, CH_2), 1.90 (4H, quint, *J* 7.7 Hz CH_2), 1.50-1.26 (12H, m, CH_2), 0.90 (6H, t, *J* 7.0 Hz, CH_3); ^{13}C NMR; δ_C (125 MHz, $CDCl_3$): 173.7, 151.3, 134.4, 114.9, 35.0, 31.8, 29.9, 29.2, 22.8, 14.3; *m/z* (MALDI-TOF): 361; HRMS calculated for $C_{20}H_{28}N_2S_2$: 361.1761. Found 361.1767.[3]

4,8-Dibromo-2,6-dihexylbenzo[1,2-d:4,5-d']bis(thiazole) ***(4a)***

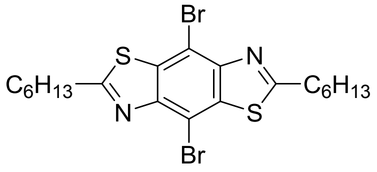

2,6-Dihexylbenzo[1,2-*d*:4,5-*d'*]bis(thiazole) (8.0 g, 22.19 mmol) **(3a)** was added to a 250 mL two neck flask under Ar. Dichloromethane (80 mL) was then added and the flask cooled to 0 °C. Bromine (2.8 mL, 55.5 mmol) in dichloromethane (80 mL) was then added dropwise while at 0 °C. The reaction was then stirred at 0 °C for 6 h and then allowed to stir at r.t. overnight. The mixture was then diluted with dichloromethane (250 mL), quenched with sat. sodium sulphite (50 mL) and washed with water (2 x 100 mL), before being dried ($MgSO_4$) and concentrated to dryness. Purification by silica gel column chromatography eluting with 3:2 hexane:dichloromethane yielded a white solid, which was recrystallized with hexane:methanol to yield the *title compound* as a white crystalline solid (5.1 g, 45%); m.p.: 114 – 117 °C; ^{1}H NMR; δ_H (400 MHz $CDCl_3$): 3.17 (4H, t, *J* 7.8 Hz, CH_2), 1.90 (4H, quint, *J* 7.7 Hz CH_2), 1.50-1.26 (12H, m, CH_2), 0.92 (6H, t, *J* 7.0 Hz, CH_3); ^{13}C NMR; δ_C (125 MHz, $CDCl_3$): 174.8, 148.1, 137.4, 106.2, 35.3, 31.8, 30.1, 29.2, 22.8, 14.4; *m/z* (MALDI-TOF): 519; Anal. Calculated for $C_{20}H_{26}Br_2N_2S_2$: C, 46.34; H, 5.06; N, 5.40. Found C, 46.46; H, 5.01; N, 5.28.[3]

2,4-Dibromothiazole ***(5)***

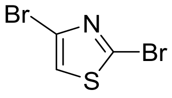

To a 100 mL round bottom flask under Ar was added thiazolidine-2,4-dione (25.0 g, 0.213 mol), phosphorus pentoxide (142.0 g, 1.003 mol) and tetrabutylammonium bromide (158.0 g, 0.491 mol) and the flask evacuated several times. Anhydrous toluene (480 mL) was then added *via* syringe and the reaction heated to reflux for 20 h under Ar. The solution was concentrated and the resulting residue partitioned between Et_2O (150 mL) and H_2O (450 mL). The aqueous layer was adjusted to pH 9 with solid Na_2CO_3. The organic layer was separated and the aqueous layer was extracted with Et_2O (3 x 75 mL). The combined organic extracts were washed with brine (200 mL), dried ($MgSO_4$) and

concentrated to give a dark brown solid. Recrystalization from hexane yielded the product as off-white crystals (41.3g, 80%); m.p.: 80 – 81 °C (81 – 82 °C lit.); ^{1}H NMR; δ_H (400 MHz $CDCl_3$): 7.22 (1H, s, Ar-H). [4]

*4-Bromothiazole (**6**)*

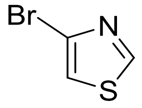

To a stirred solution of 2,4-dibromothiazole **(5)** (10.0 g, 41.2 mmol) in anhydrous diethyl ether (200 mL) under Ar at -78 °C was added a solution of *n*-butyllithium (18.5 mL, 45.3 mmol, 2.45M in hexane) dropwise. The reaction was then allowed to stir for 30 min at -78 °C before addition of methanol (3.3 g, 103 mmol) dropwise. The reaction was then slowly allowed to increase to rt overnight with stirring. The reaction mixture was filtered through a plug of silica and washed with a 1:2 mixture of ethyl acetate:hexane. The solvent was removed in *vacuo* to yield the title compound as a clear oil, which was used without further purification (6.5 g, 96%); ^{1}H NMR; δ_H (400 MHz $CDCl_3$): 8.75 (1H, d, *J* 2.2 Hz, Ar-H), 7.31 (1H, d, *J* 2.2 Hz, Ar-H). [5]

4-Methoxythiazole (7):

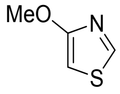

A mixture of sodium methoxide - prepared by treating anhydrous methanol (54.7 mL, 1.35 mol) with sodium (2.6 g, 114 mmol) - and 4-bromothiazole **(6)** (5.0 g, 30.5 mmol) was refluxed for 24 h. The mixture was then cooled to room temperature. The solution was then dissolved in water (100 mL) and extracted with diethyl ether (3 x 50 mL). The combined organics were washed with brine (50 mL) before being dried ($MgSO_4$) and concentrated to a deep red oil which was purified by silica gel column

chromatography eluting with 1:1 hexane:chloroform to yield the title compound as a colourless oil (2.5 g, 70%); ^{1}H NMR; δ_H (400 MHz $CDCl_3$): 8.55 (1H, d, *J* 2.3 Hz, Ar-H), 6.13 (1H, d, *J* 2.4 Hz, Ar-H), 3.93 (3H, s, CH_3). [6]

*4-((2-Ethylhexyl)oxy)thiazole (**8**):*

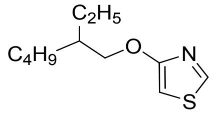

A mixture of 2-ethylhexan-1-ol (23.1 mL, 148 mmol), 4-methoxythiazole **(7)** (8.5 g, 73.8 mmol) and p-toluenesulfonic acid monohydrate (1.53 g, 8.05 mmol) in toluene (200 mL) was heated to 130 °C for 24 h. The mixture was cooled to room temperature and passed through a short plug of silica eluting with dichloromethane. Further purification *via* silica gel column chromatography eluting with 1:1 dichloromethane:hexane yielded the title compound as a pale yellow oil (11.2 g, 71%). ^{1}H NMR; δ_H (400 MHz $CDCl_3$): 8.57 (1H, d, *J* 2.3 Hz, Ar-H), 6.12 (1H, d, *J* 2.4 Hz, Ar-H), 4.01 (2H, m, CH_2), 1.80-1.70 (1H, m, CH), 1.58-1.25 (8H, m, CH_2), 0.93 (6H, m, CH_3). [6]

*4-((2-Ethylhexyl)oxy)-2-(triisopropylsilyl)thiazole **(9)***

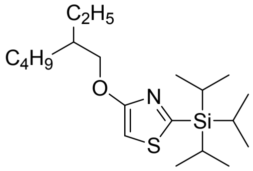

To a stirred solution of 4-((2-ethylhexyl)oxy)thiazole **(8)** (2.0 g, 9.37 mmol) in anhydrous tetrahydrofuran (80 mL) at -78 °C under Ar was added a solution of n-BuLi (3.75 mL, 9.37 mmol) in hexane dropwise over 30 min. The resulting suspension was stirred at -78 °C for 2 h before addition of chlorotriisopropylsilane (2.41 mL, 11.25 mmol). The mixture was allowed to stir and warm to rt overnight before being concentrated in vacuo and purified *via* silica gel column chromatography eluting with 6:1 hexane:dichloromethane to yield the title compound as a pale yellow oil (3.1 g, 89%). ^{1}H NMR; δ_H (400 MHz $CDCl_3$): 6.62 (1H, s, Ar-H), 4.04 (2H, m, CH_2), 1.85-1.74 (1H, m, CH), 1.60-1.23 (11H, m, CH/CH_2), 1.14 (18H, d, *J* 7.4 Hz, CH_3), 0.95-0.85 (6H, m, CH_3). [6]

4,4'-Bis((2-ethylhexyl)oxy)-2,2'-bis(triisopropylsilyl)-5,5'-bithiazole ***(10)***

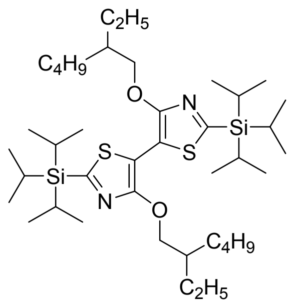

A solution of 4-((2-ethylhexyl)oxy)-2-(triisopropylsilyl)thiazole **(9)** (2.5 g, 6.76 mmol) in anhydrous tetrahydrofuran (70 mL) was cooled to -78 °C under Ar. A solution of *n*-BuLi in hexanes (2.98 mL, 7.44 mmol) was then added dropwise over 30 mins. After addition the mixture was stirred for 45 min at -78 °C then a further 1 hour at rt. The solution was then cooled to 0 °C before addition of ferric acetylacetonate (2.63 g, 7.44 mmol). The reaction was then heated to 80 °C for 2 h, filtered, and the filter cake washed with THF (3 x 25 mL). The filtrate was concentrated in *vacuo* and purified by column chromatography eluting with hexane to 9:1 hexane:dichloromethane which yielded a pale yellow oil which was recrystallized from hexane/ethanol to yield the title compound as white prisms (2.2 g, 88%); m.p.: 58 °C; ^{1}H NMR; δ_H (400 MHz $CDCl_3$): 4.59-4.38 (4H, m, CH_2), 1.81-1.72 (2H, m, CH), 1.65-1.30 (22H, m, CH/CH_2), 1.18 (36H, d, *J* 7.4 Hz, CH_3), 1.02-0.88 (12H, m, CH_3). [6]

4,4'-Bis((2-ethylhexyl)oxy)-5,5'-bithiazole ***(11)***

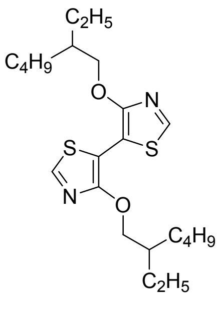

To a solution of 4,4'-bis((2-ethylhexyl)oxy)-2,2'-bis(triisopropylsilyl)-5,5'-bithiazole **(10)** (2.0 g, 2.71 mmol) in anhydrous tetrahydrofuran (25 mL) under Ar at 0 °C was added a solution of tetra-n-butylammonium fluoride (8.14 mL, 8.14 mmol) in tetrahydrofuran dropwise over 5 min. The solution was stirred at 0 °C for 30 min before being allowed to warm to rt and stirred for 90 min. Water (1 mL)

was then added to the reaction and the solvent concentrated in *vacuo* before being purified by column chromatography eluting with dichloromethane:hexane 1:1 to yield the title compound as a pale yellow oil (680 mg, 59%); ^{1}H NMR; δ_H (400 MHz $CDCl_3$): 8.40 (2H, s, Ar-H), 4.38 (4H, m, CH_2), 1.87-1.69 (2H, m, CH), 1.69-1.24 (16H, m, CH_2), 0.96 (12H, m, CH_3). [6]

4,4'-Bis((2-ethylhexyl)oxy)-2,2'-bis(trimethylstannyl)-5,5'-bithiazole ***(12)***

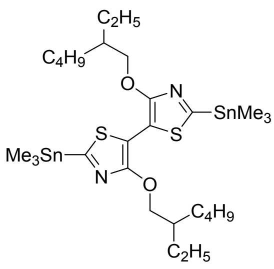

To a stirred solution of 4,4'-bis((2-ethylhexyl)oxy)-5,5'-bithiazole **(11)** (380 mg, 0.895 mmol) in anhydrous tetrahydrofuran (30 mL) under Ar at -78°C was added a solution of n-butyllithium (0.92 mL, 2.237 mmol) in hexane dropwise. The reaction was then stirred for 30 min before being allowed to warm to rt and stirred for a further 30 min. Trimethyltin chloride (3.13 mL, 3.13 mmol) (1M in THF) was then added in a single portion to the reaction and the reaction heated to 60 °C for 1 h before being carefully quenched with water (2 mL). The resulting mixture was then poured into water (100 mL) and extracted with hexane (3 x 50 mL). The combined organic layers were then washed with water (2 x 100 mL) and brine (100 mL) before being dried ($MgSO_4$) and concentrated to a red oil which was used without further purification (660 mg, 98%); ^{1}H NMR; δ_H (400 MHz $CDCl_3$): 4.48-4.29 (4H, m, CH_2), 1.83-1.67 (2H, m, CH), 1.62-1.23 (16H, m, CH_2), 1.04-0.81 (12H, m, CH_3), 0.43 (18H, s, CH_3). [6]

pBTzBBT

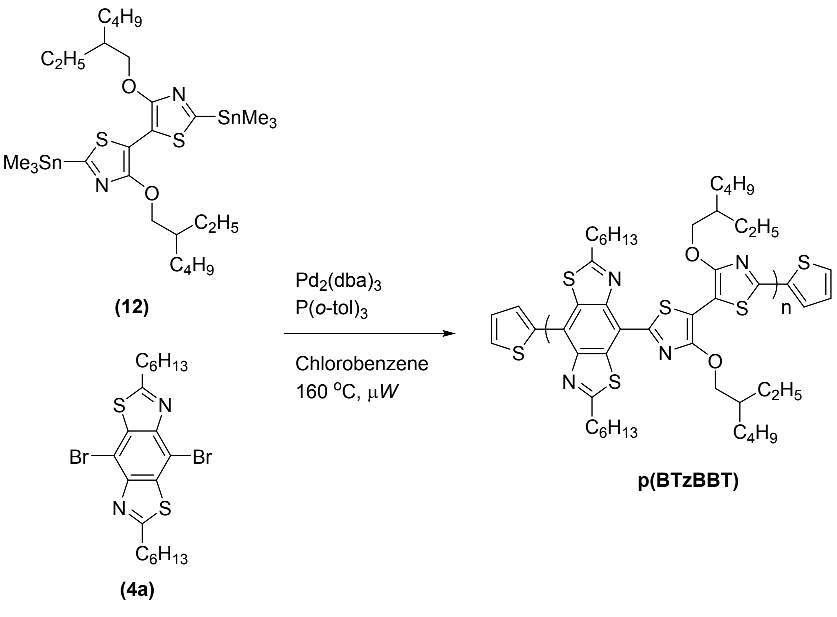

Synthesis of **pBTzBB)**

To a 5 mL microwave vial under Ar was added 4,4'-bis((2-ethylhexyl)oxy)-2,2'-bis(trimethylstannyl)-5,5'-bithiazole **(12)** (301 mg, 0.401 mmol) and 4,8-dibromo-2,6-dihexylbenzo[1,2-d:4,5-d']bis(thiazole) **(4a)** (208 mg, 0.401 mmol) and the vial purged several times with Ar. Anhydrous chlorobenzene (2 mL), $Pd_2(dba)_3$ (18 mg, 0.020 mmol) and $P(o\text{-}tol)_3$ (24 mg, 0.080 mmol) were then added and the vial purged for a further 10 min before being heated to 160 °C for 1 h under microwave irradiation. Once cool, tributyl(thiophen-2-yl)stannane (0.1 mL, 0.315 mmol) was then added and the flask heated to 160 °C for 10 min. 2-Bromothiophene (0.5 mL, 2.066 mmol) was then added and the flask heated to 160 °C for a further 10 min before being cooled to rt and precipitated into methanol (150 mL). The precipitated polymer was then purified via Soxhlet extraction with methanol, acetone, hexane and chloroform to remove low molecular weight impurities. The product was then collected in chlorobenzene and concentrated. The resulting blue residue was dissolved in the minimum volume of o-dichlorobenzene and precipitated into acetone to yield the title compound as a dark blue solid (201 mg); Anal. Calculated for $C_{42}H_{62}N_4O_2S_4$: C, 64.41; H, 7.98; N, 7.15. Found: C, 64.57; H, 7.73; N, 7.03; *GPC data not obtained due to incomplete polymer solubility.*

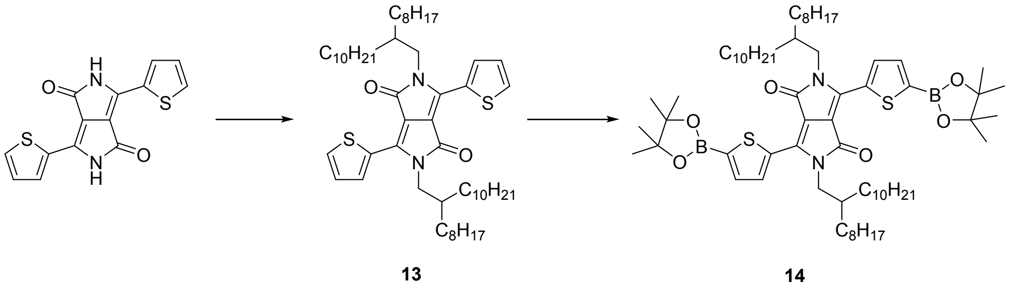

Syntheses of compounds 13 and 14

2,5-Bis(2-octyldodecyl)-3,6-di(thiophen-2-yl)pyrrolo[3,4-c]pyrrole-1,4(2H,5H)-dione ***(13)***

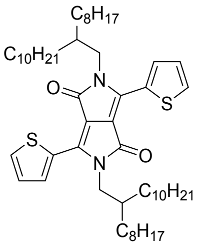

To a 250 mL oven-dried flask under Ar was added 3,6-di(thiophen-2-yl)pyrrolo[3,4-c]pyrrole-1,4(2H,5H)-dione (3.0 g, 9.99 mmol) and anhydrous potassium carbonate (4.1 g, 30.0 mmol) and the flask evacuated several times with Ar. Anhydrous dimethylformamide (85 mL) was then added and the reaction heated to 120 °C for 1 h, at which point 9-(bromomethyl)nonadecane (10.8 g, 30.0 mmol) was added dropwise. After addition the reaction was heated to 130 °C overnight. After cooling to room temperature, diethyl ether (250 mL) was added and the organic layer extracted with water (3 x 200 mL) and brine (200 mL) before being dried ($MgSO_4$) and concentrated in *vacuo*. Silica gel column chromatography eluting with hexane:ethyl acetate 20:1 yielded the title compound as a dark purple solid which was reprecipitated from dichloromethane/methanol to yield the title compound as a purple solid (3.7 g, 43%); m.p.: 70 – 74 °C; ^{1}H NMR; δ_H (400 MHz $CDCl_3$): 8.86 (2H, dd, ^4J = 1.1, ^3J = 7.7 Hz, Ar-H), 7.61 (2H, dd, ^4J = 1.1, ^3J = 5.0 Hz, Ar-H), 7.26 (2H, m, Ar-H), 4.02 (4H, d, J 7.7 Hz, Ar-H), 1.89 (2H, br, CH), 1.45-1.13 (64H, m, CH_2), 0.87 (12H, m, CH_3).[7]

2,5-Bis(2-octyldodecyl)-3,6-bis(5-(4,4,5,5-tetramethyl-1,3,2-dioxaborolan-2-yl)thiophen-2-yl)pyrrolo[3,4-c]pyrrole-1,4(2H,5H)-dione ***(14)***

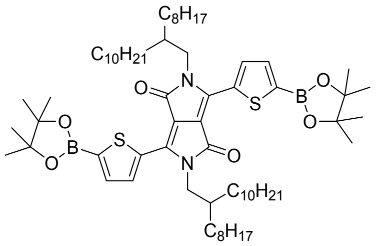

To a stirred solution of 2,5-bis(2-octyldodecyl)-3,6-di(thiophen-2-yl)pyrrolo[3,4-*c*]pyrrole-1,4(2H,5H)-dione **(13)** (1.1 g, 1.277 mmol) and 2-isopropoxy-4,4,5,5-tetramethyl-1,3,2-dioxaborolane (0.78 mL, 3.83 mmol) in anhydrous tetrahydrofuran (12 mL) at -25 °C under Ar was added a solution of lithium diisopropylamide (1.63 mL, 2.94 mmol) in tetrahydrofuran dropwise. The solution was stirred at 0 °C for 1 h before addition of a 0.1M solution of HCl (12 mL) and the product extracted with dichloromethane (3 x 25 mL). The combined organic layers were washed with water (25 mL), dried ($MgSO_4$) and concentrated to dryness. The crude mixture was dissolved in a minimal amount of dichloromethane and precipitated into cold acetone and filtered. The filtered solid was washed with cold acetone and dried in *vacuo* to yield a dark pink-red solid (0.97 g, 68%); m.p.: 165 – 168 °C (166 – 168 °C lit.); ^{1}H NMR; δ_H (400 MHz $CDCl_3$): 8.91 (2H, d, *J* 3.9 Hz, Ar-H), 7.71 (2H, d, *J* 3.9 Hz, Ar-H), 4.05 (4H, d, *J* 7.7 Hz, CH_2), 1.89 (2H, br, CH), 1.37 (24H, s, CH_3), 1.30-1.17 (64H, m, CH_2), 0.86 (12H, m, CH_3).[8]

2,6-Diundecylbenzo[1,2-d:4,5-d']bis(thiazole) ***(3b)***

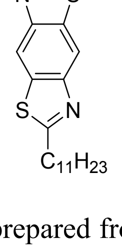

To a flask containing compound **(2)**, freshly prepared from 6 g of compound **(1)** was added a solution of trimethylsilyl polyphosphate (19.91 g, 147 mmol) in anhydrous 1,2-dichlorobenzene (50 mL) under Ar. Dodecanoyl chloride (14.15 mL, 61.2 mmol) was then added *via* syringe and the flask heated to

reflux for 48 h under Ar. Once cool the solution was diluted with dichloromethane (250 mL) and carefully washed several times with sat. $NaHCO_3$ solution. The solution was then washed with brine (2 x 200 mL) and water (2 x 200 mL) before being dried ($MgSO_4$) and the dichloromethane removed by rotary evaporation. The residual volatiles were distilled *via* vacuum distillation and the resulting dark brown residue purified *via* silica gel column chromatography eluting with dichloromethane. Reprecipitation from dichloromethane/methanol yielded the *title compound* as a crystalline white solid (8.24 g, 67 % over 2 steps); m.p.: 83 – 88 °C; ^{1}H NMR; δ_H (400 MHz $CDCl_3$): 8.38 (2H, s, Ar-H), 3.17 (4H, t, J 7.8 Hz CH_2), 1.91 (4H, quint, J 7.7 Hz CH_2), 1.51-1.20 (32H, m, CH_2), 0.88 (6H, t, J 6.8 Hz CH_3); ^{13}C NMR; δ_C (125 MHz, $CDCl_3$): 173.4, 151.0, 134.1, 114.6, 34.6, 31.9, 29.6, 29.5, 29.32, 29.28, 29.17, 22.7, 14.1; *m/z* (MALDI-TOF): 501; Anal. Calculated for $C_{30}H_{48}N_2S_2$: C, 71.94; H, 9.66; N, 5.59. Found: C, 72.00; H, 9.57; N, 5.84.

4,8-Dibromo-2,6-diundecylbenzo[1,2-d:4,5-d']bis(thiazole) ***(4b)***

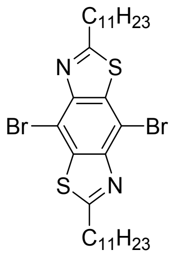

2,6-Diundecylbenzo[1,2-d:4,5-d']bis(thiazole) **(3b)** (4.5 g, 8.98 mmol) was added to a 250 mL two neck flask under Ar. Chloroform (100 mL) was then added and allowed to stir until complete dissolution. Bromine (1.2 mL, 22.46 mmol) was then added dropwise and the reaction then stirred at rt for 24 h. The reaction was then diluted with chloroform (100 mL), quenched with sat. Na_2SO_3 (50 mL) and washed with sat. $NaHCO_3$ (150 mL) and water (150 mL) before being dried ($MgSO_4$) and concentrated in *vacuo* to an off-white solid. Silica gel column chromatography eluting with 1:4 dichloromethane:hexane yielded the *title compound* as a white solid. (550 mg, 9%); m.p.: 96 – 99 °C; ^{1}H NMR; δ_H (400 MHz $CDCl_3$): 3.17 (4H, t, J 7.8 Hz, CH_2), 1.91 (4H, quint, J 7.7 Hz, CH_2), 1.51-1.20 (32H, m, CH_2), 0.88 (6H, t, J 6.8 Hz, CH_3); ^{13}C NMR; δ_C (125 MHz, $CDCl_3$): 174.5, 147.8, 137.1, 105.9, 34.9, 31.9, 29.8, 29.6, 29.4, 29.3, 29.25, 29.2, 22.7; *m/z* (MALDI-TOF): 657 (^{79}Br, 100),

659 (^{81}Br, 100); Anal. Calculated for $C_{30}H_{46}Br_2N_2S_2$: C, 54.71; H, 7.04; N, 4.25. Found: C, 54.95; H, 7.01; N, 4.14. *N.B. compound* ***(3b)*** *recovered in 54% yield after chromatography.*

pDPPThBBT

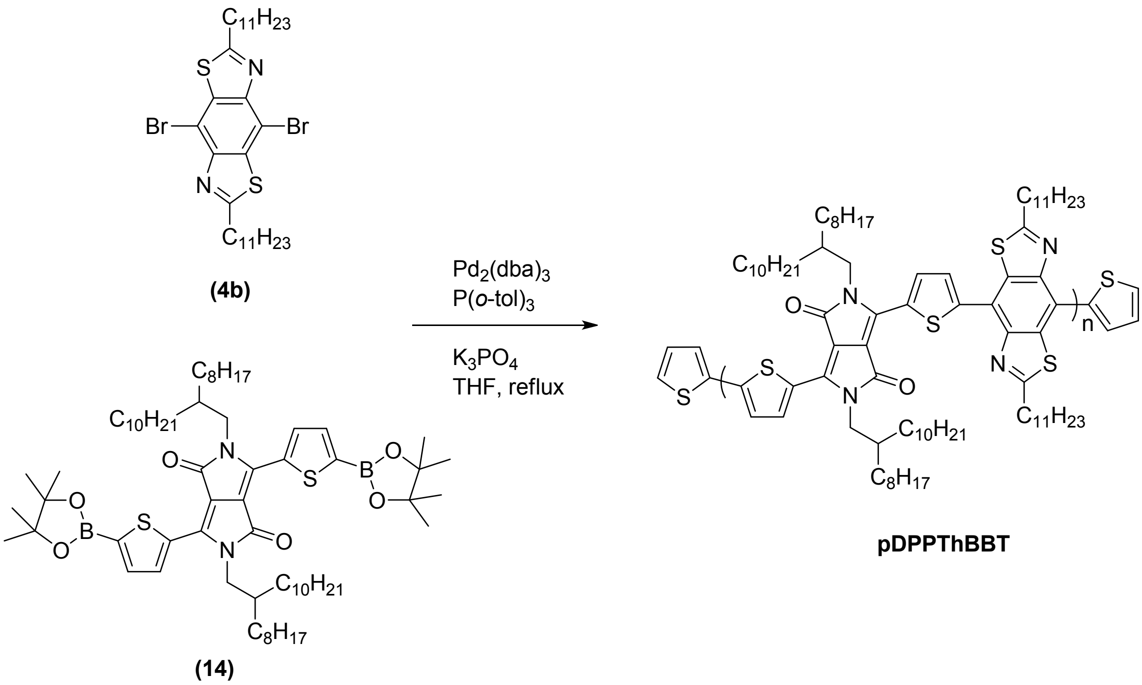

To a 25 mL two neck flask under Ar was added 2,5-bis(2-octyldodecyl)-3,6-bis(5-(4,4,5,5-tetramethyl-1,3,2-dioxaborolan-2-yl)thiophen-2-yl)pyrrolo[3,4-c]pyrrole-1,4(2H,5H)-dione **(14)** (290 mg, 0.260 mmol), 4,8-dibromo-2,6-diundecylbenzo[1,2-d:4,5-d']bis(thiazole) **(4b)** (172 mg, 0.260 mmol), P(*o*-tol)$_3$ (24 mg, 0.078 mmol) and $Pd_2(dba)_3$ (17 mg, 0.018 mmol). The flask was evacuated several times with Ar and to the flask was added anhydrous THF (10 mL). A solution of potassium phosphate (79 mg, 0.372 mmol) in degassed water (1.5 mL) was added to this solution and the mixture stirred at reflux under Ar for 24 h. To the reaction was then added thiophen-2-ylboronic acid (17mg, 0.130 mmol), P(o-tol)$_3$ (12 mg, 0.039 mmol) and $Pd_2(dba)_3$ (8 mg, 7.81 µmol) and the flask stirred at reflux for a further 2 h before addition of 2-bromothiophene (0.5 mL) and the reaction stirred for a further 2 h at reflux. The mixture was then cooled to rt, poured into cold methanol (400 mL) and filtered. The crude product was then subjected to Soxhlet extraction with methanol, acetone and chloroform respectively. The chloroform fraction was concentrated in *vacuo* then dissolved in the

minimum volume of chloroform, precipitated into acetone and filtered yielding the *title compound* as a dark green-black solid (292mg); ^{1}H NMR; δ_H (400 MHz $CDCl_3$): 9.4-8.8 (2H, br, Ar-H), 8.4-7.6 (2H br, Ar-H), 4.5-3.8 (4H, br, CH_2), 3.6-3.1 (4H, br, CH_2), 2.4-2.2 (2H, br, CH), 2.15-1.85 (4H, br, CH_2), 1.85-0.65 (96H, br, CH_2), 1.0-0.7 (18H, br, CH_3); Anal. Calculated for $C_{84}H_{134}N_4O_2S_4$: C, 74.17; H, 9.93; N, 4.12. Found: C, 72.33; H, 9.59; N, 4.09; GPC: M_n = 9 kDa, M_w = 14 kDa, PDI = 1.68.

3,6-Bis(5-bromothiophen-2-yl)-2,5-bis(2-octyldodecyl)pyrrolo[3,4-c]pyrrole-1,4(2H,5H)-dione ***(15)***

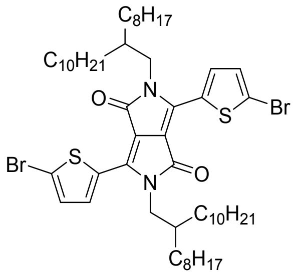

To a stirred solution of 2,5-bis(2-octyldodecyl)-3,6-di(thiophen-2-yl)pyrrolo[3,4-c]pyrrole-1,4(2H,5H)-dione **(13)** (2.0 g, 2.322 mmol) in chloroform (15 mL) in the absence of light was added a solution of bromine (0.25 mL, 4.76 mmol) in chloroform (5 mL) dropwise at rt. The solution was then allowed to stir at rt overnight before being poured into sat. aqueous sodium sulfite (20 mL) and stirred. The product was extracted with chloroform (3 x 50 mL) and washed with water (2 x 50 mL) before being dried ($MgSO_4$) and concentrated to dryness. Silica gel column chromatography eluting with 3:7 dichloromethane:hexane yielded the title compound as a purple solid which was reprecipitated from dichloromethane/methanol (2.1 g, 90%); m.p.: 88 – 90 °C; ^{1}H NMR; δ_H (400 MHz $CDCl_3$): 8.62 (2H, d, J 4.2 Hz, Ar-H), 7.22 (2H, d, J 4.2 Hz, Ar-H), 3.92 (4H, d, J 7.7 Hz, CH_2), 1.88 (2H, br, CH), 1.47-1.10 (64H, m, CH_2), 0.86 (12H, m, CH_3). [7]

2,5-Bis(trimethylstannyl)thiophene ***(16)***

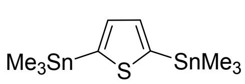

To a stirred solution of thiophene (0.46 mL, 5.94 mmol) and *N,N,N',N'*-tetramethylethylene-1,2-diamine (1.82 mL, 12.18 mmol) in anhydrous hexane (15 mL) was added a solution of *n*-butyllithium (4.99 mL, 12.18 mmol, 2.4 M in hexane) dropwise over 10 min. The reaction was then heated to reflux for 45 min before being cooled to 0 °C. A solution of chlorotrimethylstannane (17.83 mL, 17.83 mmol) (1M in THF) was then added over 10 min and the reaction stirred at rt overnight before being quenched with sat. ammonium chloride solution (100 mL). The aqueous layer was then extracted with diethyl ether (3 x 50 mL) and washed with brine (2 x 50 mL) before being dried ($MgSO_4$) and concentrated to a yellow solid which was further purified by recrystallization from hexane to yield the title compound as white needles (1.93 g, 79%); ^{1}H NMR; δ_H (400 MHz $CDCl_3$): 7.40 (2H, s, Ar-H), 0.39, (18H, s, CH_3).[9]

pDPP2ThBBT

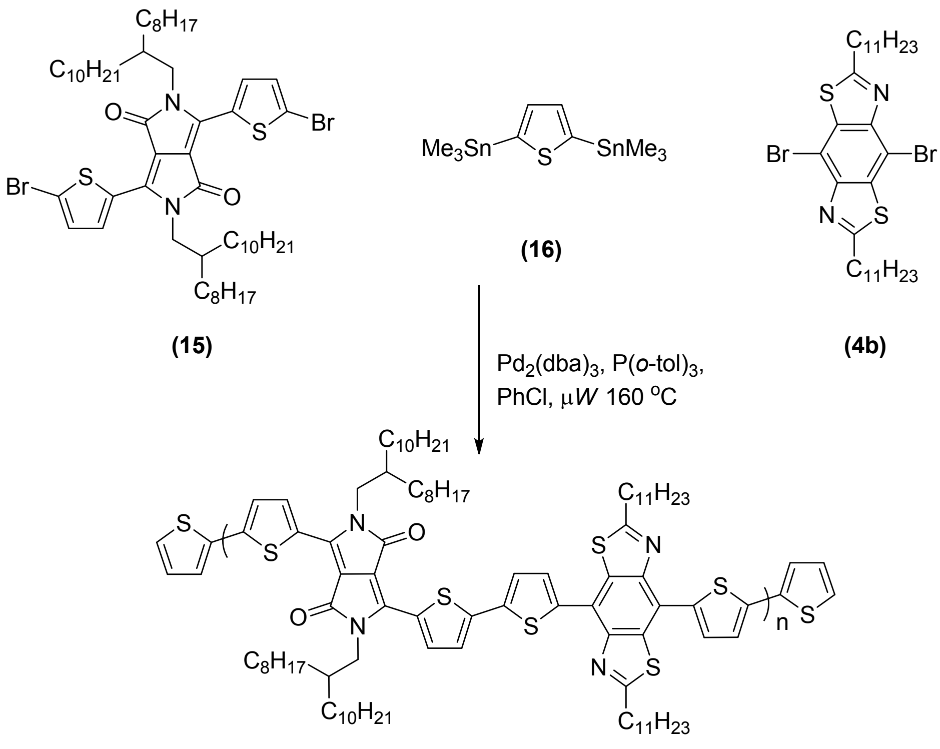

pDPP2ThBBT

To a 5 mL microwave vial under Ar was added 2,5-bis(trimethylstannyl)thiophene **(16)** (195 mg, 0.477 mmol), 4,8-dibromo-2,6-diundecylbenzo[1,2-d:4,5-d']bis(thiazole) **(4b)** (157 mg, 0.238 mmol) and 3,6-bis(5-bromothiophen-2-yl)-2,5-bis(2-octyldodecyl)pyrrolo[3,4-c]pyrrole-1,4(2H,5H)-dione **(15)** (243mg, 0.238 mmol) and the flask purged with Ar for 10 min. Anhydrous chlorobenzene (2 mL) was then added *via* syringe and the vial purged for a further 15 min with Ar. $Pd_2(dba)_3$ (11 mg, 0.012 mmol) and $P(o\text{-}tol)_3$ (15 mg, 0.048 mmol) were then added and the vial purged for a final 5 min. The reaction was then heated to 160 °C for 1 h before being cooled to rt and tributyl(thiophen-2-yl)stannane (0.1 mL) added. The flask was then heated to 160 °C for 10 min before addition of 2-bromothiophene (0.5 mL) and heated to 160 °C for a further 10 min. The crude solution was then precipitated into methanol (400 mL) and filtered. The crude product was then subjected to Soxhlet extraction with methanol, acetone and chloroform respectively. The chloroform fraction was concentrated in *vacuo* then dissolved in the minimum volume of chloroform, precipitated into acetone and filtered yielding the *title compound* as a dark green-black solid (351 mg); Anal. Calculated for $C_{92}H_{138}N_4O_2S_6$: C, 72.48; H, 9.12; N, 3.68. Found: C, 72.37; H, 9.06; N, 3.55; GPC: M_n = 35 kDa, M_w = 96 kDa, PDI = 2.75.

2,5-Bis(trimethylstannyl)furan ***(17)***

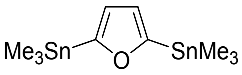

To a stirred solution of furan (0.53 mL, 7.34 mmol) and *N,N,N',N'*-tetramethylethylene-1,2-diamine (2.75 mL, 18.36 mmol) in anhydrous hexane (15 mL) at 0 °C was added a solution of sec-butyllithium in hexane (15.05 mL, 18.36 mmol) dropwise over 10 min. The reaction was stirred for 1 h at 0 °C followed by rt for 4 h after which the solution was cooled to 0 °C again. A solution of chlorotrimethylstannane (22.03 mL, 22.03 mmol) in THF was then added over 10 min and the reaction stirred at rt overnight before being quenched with sat. ammonium chloride solution (100 mL). The aqueous layer was then extracted with diethyl ether (3 x 50 mL) and washed with brine (2 x 50 mL) before being dried ($MgSO_4$) and concentrated to a yellow oil which was purified by Kugelrohr

distillation (bp 140 °C, low vacuum) to yield the title compound as a pale yellow oil (1.98 g, 69%); ^{1}H NMR; δ_H (400 MHz $CDCl_3$): 6.63 (2H, s, Ar-H), 0.32 (18H, s, CH_3). [9]

pDPPThFBBT

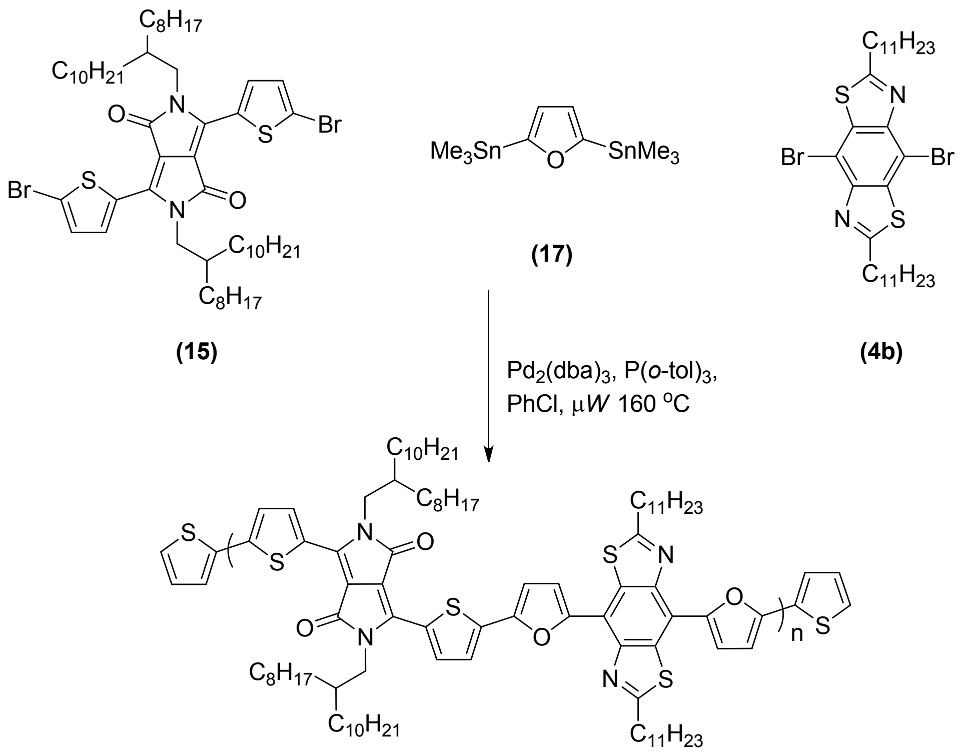

pDPPThFBBT

To a 5 mL microwave vial under Ar was added 2,5-bis(trimethylstannyl)furan **(17)** (161mg, 0.409 mmol), 4,8-dibromo-2,6-diundecylbenzo[1,2-d:4,5-d']bis(thiazole) **(4b)** (135 mg, 0.204 mmol) and 3,6-bis(5-bromothiophen-2-yl)-2,5-bis(2-octyldodecyl)pyrrolo[3,4-c]pyrrole-1,4(2H,5H)-dione **(15)** (208 mg, 0.204 mmol) and the flask purged with Ar for 10 min. Anhydrous chlorobenzene (2 mL) was then added *via* syringe and the vial purged for a further 15 min with Ar. $Pd_2(dba)_3$ (9 mg, 10.22 μmol) and P(o-tol)$_3$ (12 mg, 0.041 mmol) were then added and the vial purged for a final 5 minutes. The reaction was then heated to 160 °C for 1 h before being cooled to rt and tributyl(thiophen-2-yl)stannane (0.1 mL) added. The flask was then heated to 160 °C for 10 mins before addition of 2-bromothiophene (0.5 mL) and heated to 160 °C for a further 10 mins. The crude solution was then precipitated into methanol (400 mL) and filtered. Soxhlet extraction with methanol, hexane and

acetone removed low molecular weight impurities and the product was collected *via* Soxhlet extraction using chloroform and precipitated into acetone to yield the product as a dark green solid (195 mg); Anal. Calculated for $C_{92}H_{138}N_4O_4S_4$: C, 74.04; H, 9.32; N, 3.75. Found: C, 73.63; H, 8.85; N, 3.77; GPC: M_n = 9 kDa, M_w = 18 kDa, PDI = 2.04.

3-(2-Ethylhexyl)thiophene ***(18)***

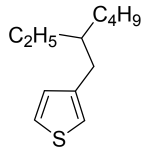

To a suspension of magnesium turnings (1.61 g, 66.2 mmol) in anhydrous tetrahydrofuran (5 mL) was added a solution of 2-ethylhexyl bromide (10.93 mL, 61.1 mmol) in anhydrous tetrahydrofuran (15 mL) at a rate sufficient to maintain gentle reflux. After addition, reflux was maintained for 2 h before being cooled to rt and transferred dropwise *via* syringe into a flask containing a solution of 3-bromothiophene (4.77 mL, 50.9 mmol) and dichloro(1,3-bis(diphenylphosphino)propane)nickel (0.276 g, 0.509 mmol) in anhydrous tetrahydrofuran (30 mL). The resulting solution was stirred at reflux under Ar overnight. After cooling to rt the reaction was quenched with water (10 mL) followed by conc. HCl (2 mL) and extracted with dichloromethane (3 x 50 mL). The combined organic phase was washed with brine (100 mL) and water (100 mL) before being dried ($MgSO_4$) and concentrated to a yellow oil, which was purified by Kughelrohr distillation (b.p, 110 °C, low vacuum) to yield the title compound as a colourless oil (8.0 g, 80%); ^{1}H NMR; δ_H (400 MHz $CDCl_3$): 7.22 (1H, dd, ^4J = 3.0 ^4J = 4.9 Hz, Ar-H), 6.93-6.87 (2H, m, Ar-H), 2.57 (2H, d, J 6.9 Hz, CH_2), 1.68-1.48 (1H, m, CH), 1.36-1.17 (8H, m, CH_2), 0.99-0.80 (6H, m, CH_3).[10]

(4-(2-Ethylhexyl)thiophen-2-yl)trimethylstannane ***(19)***

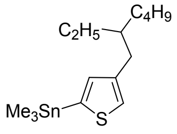

A solution of n-butyllithium (2.31 mL, 5.63 mmol) in hexane was added to a flask containing 3-(2-ethylhexyl)thiophene **(18)** (1.1 g, 5.60 mmol) in diethyl ether (25 ml) and N,N,N',N' -tetramethylethylenediamine (0.93 mL, 6.16 mmol) under Ar at room temperature. The mixture was then allowed to reflux for 1 hr before being cooled to 0 °C using an ice bath. A solution of trimethyltinchloride (8.40 mL, 8.40 mmol) (1M in THF) was then added and allowed to stir overnight. The mixture was quenched using sat. ammonium chloride solution (20 mL) before being washed with brine (50 mL), dried ($MgSO_4$) and concentrated to a pale yellow oil which was used without further purification (2.0 g, 98%); ^{1}H NMR; δ_H (400 MHz $CDCl_3$): 7.19 (1H, s, Ar-H), 6.99 (1H, s, Ar-H), 2.61 (2H, d, *J* 6.8 Hz), 1.59 (1H, m, CH), 1.30 (8H, m, CH_2), 0.90 (6H, m, CH_3), 0.38 (9H, s, CH_3).[10]

4,7-Bis(4-(2-ethylhexyl)thiophen-2-yl)benzo[c][1,2,5]thiadiazole ***(20)***

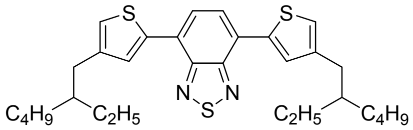

To a 25 mL flask under Ar was added 4,7-dibromobenzo[c][1,2,5]thiadiazole (200 mg, 0.68 mmol), (4-(2-ethylhexyl)thiophen-2-yl)trimethylstannane **(19)** (733 mg, 2.041 mmol) and tetrakis(triphenylphosphine) palladium(0) (157 mg, 0.136 mmol), and the flask purged several times with Ar. Anhydrous toluene (10 mL) was then added and the flask heated to reflux for 24 h. Once cool the solution was poured into water (20 mL) and extracted with dichloromethane (3 x 50 mL). The combined organic layers were washed with water (2 x 50 mL) and brine (50 mL) before being dried ($MgSO_4$) and concentrated to dryness. The crude product was purified by silica gel column chromatography eluting with 20:1 hexane:dichloromethane to yield the product as an orange solid which was further recrystallised from ethanol to yield the title compound as a bright orange solid (302 mg, 85%); m.p.: 49 – 50 °C; ^{1}H NMR; δ_H (400 MHz $CDCl_3$): 7.97 (2H, s, Ar-H), 7.86 (2H, s, Ar-H), 7.04 (2H, s, Ar-H), 2.66 (4H, d, *J* 6.9 Hz, CH_2), 1.68 (2H, m, CH), 1.35 (16H, m, CH_2), 0.92 (12H, t, *J* 7.4 Hz, CH_3).[10]

4,7-Bis(5-bromo-4-(2-ethylhexyl)thiophen-2-yl)benzo[c][1,2,5]thiadiazole ***(21)***

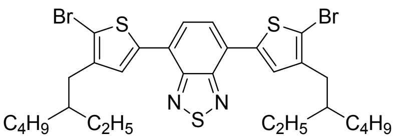

To a stirred solution of 4,7-bis(4-(2-ethylhexyl)thiophen-2-yl)benzo[c][1,2,5]thiadiazole **(20)** (240 mg, 0.457 mmol) in anhydrous tetrahydrofuran (20 mL) under Ar and in the absence of light was added *N*-bromosuccinimide (203 mg, 1.143 mmol) in several portions at 0 °C. After addition, the reaction was stirred for a further 30 min before being warmed to rt and stirred overnight. The reaction was then quenched with sat. NH_4Cl solution, extracted with dichloromethane (3 x 50 mL) and the combined organic layers washed with water (2 x 50mL) before being dried ($MgSO_4$) and concentrated to dryness. The pure product was obtained *via* silica gel column chromatography eluting with hexane to yield the product as a dark red oil (305 mg, 98%); ^{1}H NMR; δ_H (400 MHz $CDCl_3$): 7.75 (4H, m, Ar-H), 2.58 (4H, d, *J* 7.2 Hz, CH_2), 1.77-1.62 (2H, m, CH), 1.45-1.25 (16H, m, CH_2), 0.97-0.85 (12H, m, CH_3).[10]

pBT2ThBBT

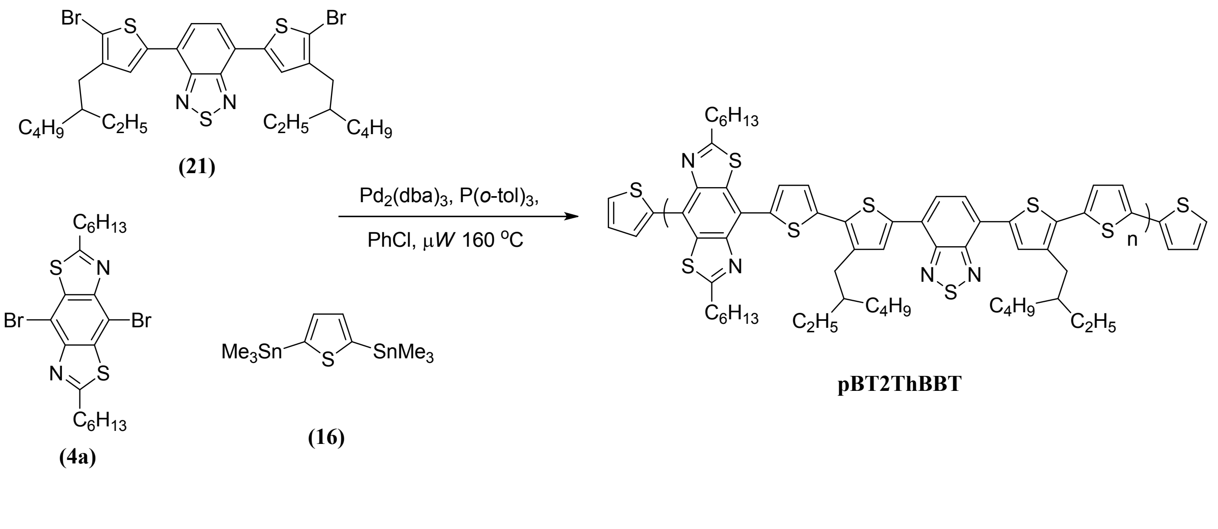

To a 20 mL microwave vial under Ar was added 2,5-bis(trimethylstannyl)thiophene **(16)** (420 mg, 1.025 mmol), 4,8-dibromo-2,6-dihexylbenzo[1,2-d:4,5-d']bis(thiazole) **(4a)** (266 mg, 0.513 mmol) and 4,7-bis(5-bromo-4-(2-ethylhexyl)thiophen-2-yl)benzo[c][1,2,5]thiadiazole **(21)** (350 mg, 0.513 mmol) and the flask purged several times with Ar. P(*o*-tol)$_3$ (31 mg, 0.103 mmol) and $Pd_2(dba)_3$ (23 mg, 0.026 mmol) were then added followed by anhydrous chlorobenzene (5 mL) and the solution purged with Ar for 15 min. The vial was then heated to 160 °C in the microwave for 50 min. Once cool, tributyl(thiophen-2-yl)stannane (0.1 mL) was then added and the flask heated to 160 °C for 10 min followed by addition of 2-bromothiophene (0.5 mL) and heating for a final 10 min. The crude reaction mixture was precipitated into methanol (400 mL) and the resulting precipitate was purified *via* Soxhlet extraction with methanol, acetone and chloroform, followed by collection of the product with chlorobenzene which was then concentrated in *vacuo* and precipitated into acetone to yield the product as a dark purple-blue solid (512 mg); Anal. Calculated for $C_{58}H_{70}N_4S_7$: C, 66.50; H, 6.74; N, 5.35. Found: C, 66.29; H, 6.47; N, 5.41; GPC: M_n = 9 kDa, M_w = 17 kDa, PDI = 1.8.

*2-Hexyldecyl methanesulfonate **(22)***

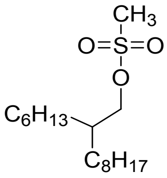

To a 250 mL round bottom flask under Ar was added 2-hexyldecan-1-ol (4.78 mL, 16.50 mmol), methanesulfonyl chloride (1.41 mL, 18.15 mmol) and anhydrous dichloromethane (80 mL). Triethylamine (2.53 mL, 18.15 mmol) was then added dropwise *via* syringe. After stirring for 1 hour at rt the solvent was removed in *vacuo* and the oily residue was extracted with diethyl ether (150 mL) and washed with water (3 x 100 mL) before being dried ($MgSO_4$) and concentrated to a yellow oil, which was purified by column chromatography (9:1 hexane:dichloromethane) to yield the title compound as a colourless oil (3.7 g, 70%); ^{1}H NMR; δ_H (400 MHz $CDCl_3$): 4.09 (2H, d, *J* 5.5 Hz, CH_2), 2.96 (3H, s, CH_3), 1.68 - 1.66 (1H, m, CH), 1.42-1.25 (22H, m, CH_2), 0.86 ppm (6H, m, CH_3).[11]

4,8-Bis((2-hexyldecyl)oxy)benzo[1,2-b:4,5-b']dithiophene ***(23)***

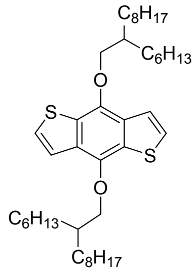

To a 50 mL round bottom flask under Ar was added tetrabutylammonium bromide (220 mg, 0.681 mmol), benzo[1,2-b:4,5-b']dithiophene-4,8-dione (500 mg, 2.270 mmol), zinc powder (386 mg, 5.90 mmol), sodium hydroxide pellets (1.362 g, 34.0 mmol) and degassed water (7 mL), and the flask heated to reflux for 1 h, then 2-hexyldecyl methanesulfonate **(22)** (2.183 g, 6.81 mmol) was added and the reaction heated to reflux for a further 2 h yielding an orange suspension. An additional portion of zinc powder (148 mg, 2.270 mmol) was added and then the reaction heated to reflux overnight. The reaction was then poured into water and extracted with diethyl ether (3 x 50 mL) and the combined organic layer washed with water (2 x 50 mL) before being dried ($MgSO_4$) and concentrated to an orange oil. The crude product was purified by silica gel column chromatography eluting with hexane to yield the title compound as a pale yellow oil (1.28 g, 84%); ^{1}H NMR; δ_H (400 MHz $CDCl_3$): 7.47 (2H, d, *J* 5.5 Hz, Ar-H), 7.36 (2H, d, *J* 5.5 Hz, Ar-H), 4.20 (4H, d, *J* 5.4 Hz, CH_2), 1.89 (2H, m, CH), 1.65 (8H, m, CH_2), 1.45-1.35 (40H, m, CH_2), 0.93 (12H, m, CH_3).[11]

(4,8-Bis((2-hexyldecyl)oxy)benzo[1,2-b:4,5-b']dithiophene-2,6-diyl)bis(trimethylstannane) ***(24)***

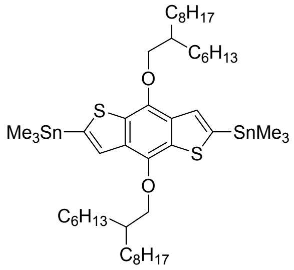

To a solution of 4,8-bis((2-hexyldecyl)oxy)benzo[1,2-b:4,5-b']dithiophene **(23)** (800 mg, 1.192 mmol) in anhydrous tetrahydrofuran (15 mL) at -78 °C was added a solution of *n*-butyllithium (1.19 mL, 2.86

mmol, 2.4M in hexane) dropwise under Ar. This mixture was stirred at -78 °C for 15 mins before being warmed to rt and stirred for 1.5 h. The solution was subsequently cooled to 0 °C and trimethyltin chloride (3.58 mL, 3.58 mmol, 1M in tetrahydrofuran) added. The solution was allowed to warm to rt and stirred overnight. After this time the reaction was quenched with water (100 mL) and extracted with diethyl ether (3 x 100 mL). The combined organic layers were washed with water (3 x 50 mL) before being dried ($MgSO_4$) and concentrated to dryness. The resulting pale yellow oil was recrystallized from isopropanol to yield the title compound as a white solid (725 mg, 61%); ^{1}H NMR; δ_H (400 MHz $CDCl_3$): 7.51 (2H, s, Ar-H), 4.18 (4H, d, J 5.3 Hz, CH_2), 1.96-1.80 (2H, m, CH), 1.73-1.58 (4H, m, CH_2), 1.52-1.15 (44H, m, CH_2), 1.0-0.77 (12H, m, CH_3), 0.44 (18H, s, CH_3).[11]

pBDTBBT

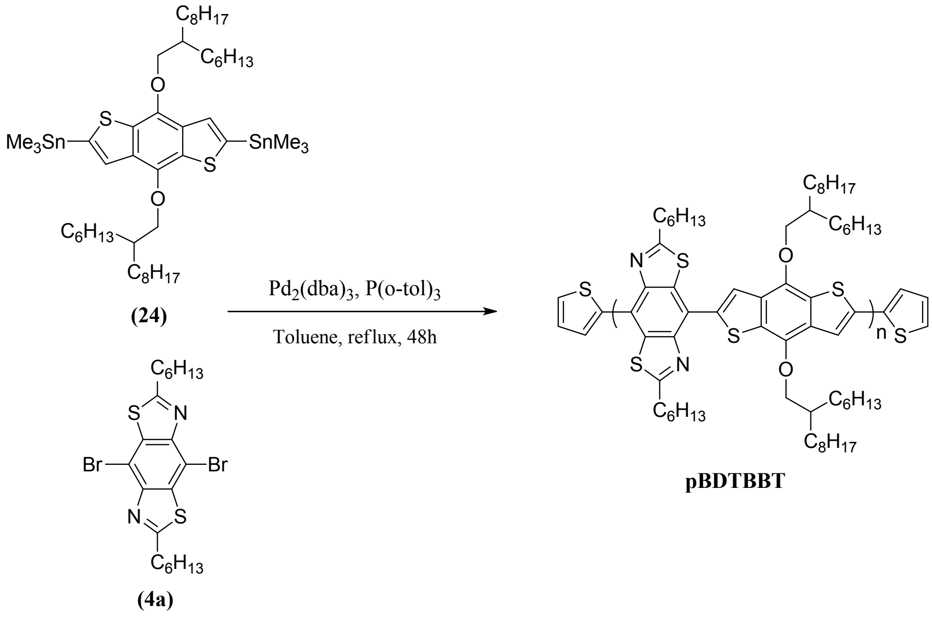

To a 25 mL round bottom flask under Ar was added (4,8-bis((2-hexyldecyl)oxy)benzo[1,2-b:4,5-b']dithiophene-2,6-diyl)bis(trimethylstannane) **(24)** (299 mg, 0.3 mmol) and 4,8-dibromo-2,6-dihexylbenzo[1,2-d:4,5-d']bis(thiazole) **(4a)** (156 mg, 0.300 mmol) and the flask purged with Ar several times before addition of anhydrous toluene (10 mL) *via* syringe. The solution was then bubbled with Ar for 5 min and then $Pd_2(dba)_3$ (13 mg, 0.015 mmol) and P(*o*-tol)$_3$ (18 mg, 0.060 mmol) added. The solution was then heated to reflux for 48 h under Ar. 2-Tributylstannyl thiophene (0.1 mL) was then added *via* syringe and the reaction heated to reflux for a further 8 h before addition of 2-

bromothiophene (0.5 mL) and heating overnight. The reaction mixture was cooled to rt and precipitated into methanol (100 mL). The precipitate was filtered into a Soxhlet thimble and extracted with methanol, acetone and hexane before being collected in chloroform. The chloroform fraction was then passed through a short silica gel plug to remove inorganic impurities before being concentrated and precipitated into acetone to yield the title compound as a red solid (284 mg); Anal. Calculated for $C_{62}H_{96}N_2O_2S_4$: C, 72.32; H, 9.40; N, 2.72. Found: C, 72.26; H, 9.13; N, 2.69; GPC: M_n = 31.5 kDa, M_w = 60.7 kDa, PDI = 1.93.

*7-(Bromomethyl)pentadecane **(25)***

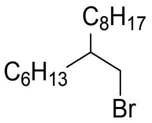

A solution of triphenylphosphine (10.82 g, 41.2 mmol) in dichloromethane (60 mL) was purged with Ar for 15 min before addition of Br_2 (2.13 mL, 41.2 mmol). 2-Hexyldecan-1-ol (11.96 mL, 41.2 mmol) was then added dropwise over 30 mins and the reaction left to stir at rt overnight. The solvent was removed in *vacuo* and the resulting residue washed with hexane (100 mL) and filtered. The filtrate was concentrated under reduced pressure and purified *via* silica gel column chromatography eluting with 20% dichloromethane in hexanes to yield the title compound as a colourless oil (12.3 g, 97%); ^{1}H NMR; δ_H (400 MHz $CDCl_3$): 3.45 (2H, d, J 4.8 Hz, CH_2), 1.68-1.51 (1H, m, CH), 1.46-1.15 (24H, m, CH_2), 1.02-0.80 (6H, m, CH_3).[12]

*2-(2-Hexyldecyl)isoindoline-1,3-dione **(26)***

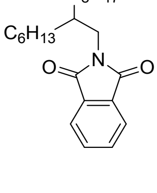

To a stirred solution of 7-(bromomethyl)pentadecane **(25)** (3.0 g, 9.83 mmol) in anhydrous DMF (10 mL) was added potassium phthalimide (1.911 g, 10.32 mmol) under Ar. The reaction was then heated

to 90 °C for 18 h before being cooled to rt, poured into water (50 mL) and extracted with diethyl ether (3 x 30 mL). The combined organic layers were then washed with 0.2 M KOH (50 mL), water (50 mL) and sat NH_4Cl (50 mL) before being dried ($MgSO_4$) and concentrated to a crude yellow oil. The crude oil was purified *via* silica gel column chromatography eluting with dichloromethane to yield the title compound as a colourless oil (3.43 g, 94%); ^{1}H NMR; δ_H (400 MHz $CDCl_3$): 7.9-7.84 (2H, m, Ar-H), 7.76-7.70 (2H, m, Ar-H), 3.57 (2H, d, J 7.3 Hz, CH_2), 1.93-1.82 (1H, m, CH), 1.45-1.15 (24H, m, CH_2), 0.96-0.78 (6H, m, CH_3).[12]

2-Hexyldecan-1-amine ***(27)***

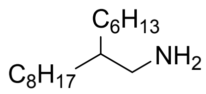

2-(2-Hexyldecyl)isoindoline-1,3-dione **(26)** (3.4 g, 9.15 mmol), hydrazine hydrate (0.852 mL, 27.5 mmol) and methanol (40 mL) were stirred at 95 °C until complete consumption of the starting material was observed (6 h). The residual solvent was removed in *vacuo* and the resulting residue diluted with dichloromethane (100 mL) and washed with 10% KOH solution (2 x 50 mL). The combined aqueous layers were extracted with dichloromethane (3 x 30 mL). The combined organic layers were then washed with brine (2 x 50 mL) before being dried ($MgSO_4$) and concentrated to a yellow oil which was passed through a short silica plug eluting with ethyl acetate to yield the title compound as a colourless oil (1.96 g, 89%); ^{1}H NMR; δ_H (400 MHz $CDCl_3$): 2.60 (2H, d, J = 5.0 Hz, CH_2), 1.39 (1H, m, CH), 1.45-1.15 (24H, m, CH_2), 0.96-0.78 (6H, m, CH_3).[12]

3,3'-Dibromo-2,2'-bithiophene ***(28)***

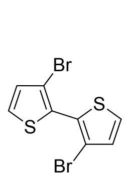

To a stirred solution of diisopropylamine (9.52 mL, 67.5 mmol) in anhydrous tetrahydrofuran (60 mL) was added a solution of *n*-butyllithium (25.6 mL, 61.3 mmol, 2.39M in hexane) dropwise at -78 °C under Ar. The solution was then allowed to warm to rt and stirred for 15 min before being cooled to -

78 °C and 3-bromothiophene (5.75 mL, 61.3 mmol) added dropwise. The suspension was then stirred for 1 h at low temperature before careful addition of copper (II) chloride (8.66 g, 64.4 mmol) in one portion. The mixture was then allowed to warm to rt and treated with aqueous HCl (10 mL) and extracted with diethyl ether (3 x 150 mL) before being washed with water (2 x 100 mL), dried ($MgSO_4$) and concentrated to dryness. The crude product was purified *via* silica gel column chromatography eluting with hexane and further purified *via* recrystallization from hexane to yield the title compound as a pale yellow solid (4.54 g, 46% yield); m.p.: 101 – 102 °C (98 – 99 °C lit.); ^{1}H NMR; δ_H (400 MHz $CDCl_3$): 7.41 (2H, d, J 5.4 Hz, Ar-H), 7.08 (2H, d, J 5.4 Hz, Ar-H).[13]

4-(2-Hexyldecyl)-4H-dithieno[3,2-b:2',3'-d]pyrrole ***(29)***

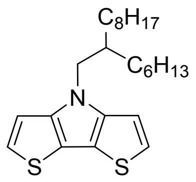

A solution of sodium tert-butoxide (741 mg, 7.71 mmol), $Pd_2(dba)_3$ (141 mg, 0.154 mmol), 2,2'-bis(diphenylphosphenyl)-1,1'-binaphthalene (384 mg, 0.617 mmol) and 3,3'-dibromo-2,2'-bithiophene **(28)** (1.0g, 3.09 mmol) in anhydrous toluene (10 mL) was purged with Ar for 20 min before addition of 2-hexyldecan-1-amine **(27)** (820 mg, 3.39 mmol) *via* syringe and the reaction heated to 110 °C for 12 h under Ar. Once cool, the reaction was poured into water (100 mL) and extracted with ethyl acetate (3 x 50 mL). The combined organic layers were washed with water (50 mL) before being dried ($MgSO_4$) and concentrated to a pale yellow oil. The crude product was purified via silica gel column chromatography eluting with hexane to yield the *title compound* as a colourless oil (854 mg, 69%); ^{1}H NMR; δ_H (400 MHz $CDCl_3$): δ 7.12 (2H, d, J = 5.3 Hz, Ar-H), 6.98 (2H, d, J = 5.3 Hz, Ar-H), 4.05 (2H, d, J = 7.3 Hz, CH_2), 2.00 (1H, br, CH), 1.41 – 1.12 (24H, m, CH_2), 0.97 – 0.79 (6H, m, CH_3); ^{13}C NMR; δ_c (125 MHz $CDCl_3$): 145.3, 122.7, 114.6, 111.1, 51.7, 39.1, 31.9, 31.8, 31.6, 29.9, 29.6, 29.5, 29.2, 26.4, 22.7, 22.6, 14.10, 14.06; *m/z* (MALDI-TOF): 403.

4-(2-Hexyldecyl)-2,6-bis(tributylstannyl)-4H-dithieno[3,2-b:2',3'-d]pyrrole ***(30)***

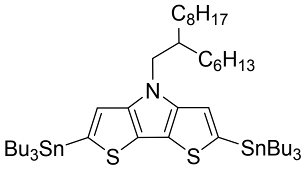

To a stirred solution of 2,2,6,6-tetramethylpiperidine (0.41 mL, 2.477 mmol) in anhydrous tetrahydrofuran (10 mL) under Ar at -78 °C was added a solution of *n*-butyllithium (0.95 mL, 2.378 mmol, 2.5M in hexane) dropwise *via* syringe. The reaction was then stirred for 1 h at -78 °C before addition of 4-(2-Hexyldecyl)-4H-dithieno[3,2-b:2',3'-d]pyrrole **(29)** (400 mg, 0.991 mmol) in anhydrous tetrahydrofuran (10 mL). The reaction was stirred for 10 min at -78 °C before being warmed to rt and stirred for 1 h. The reaction was then cooled to -78 °C and tributyltin chloride (0.59 mL, 2.180 mmol) added in one portion and the reaction allowed to warm to rt and stirred overnight. The reaction was then quenched with sat. ammonium chloride solution (50 mL) and extracted with diethyl ether (3 x 20 mL). The combined organic layers were then washed with brine (20 mL) and water (20 mL) before being dried ($MgSO_4$) and concentrated in *vacuo*. The resulting oil was purified *via* silica gel column chromatography (pre-treated with triethylamine) eluting with hexane to yield the title compound as a pale yellow oil (921 mg, 95%); ^{1}H NMR; δ_H (400 MHz $CDCl_3$): 6.92 (2H, s, Ar-H), 4.05 (2H, d, J = 7.1 Hz, CH_2), 1.99 (1H, br, CH), 1.69 - 1.55 (12H, m, CH_2), 1.45 - 1.05 (48H, m, CH_2), 0.95 – 0.80 (24H, m, CH_3); ^{13}C NMR; δ_c (125 MHz $CDCl_3$): 148.3, 134.6, 120.1, 118.2, 51.6, 39.2, 31.2, 31.8, 31.7, 30.0, 29.6, 29.3, 29.1, 29.0, 28.9, 27.3, 26.5, 26.4, 22.7, 22.6, 14.1, 13.7, 12.6, 10.9; *m/z* (MALDI-TOF): 982.

pDTPBBT

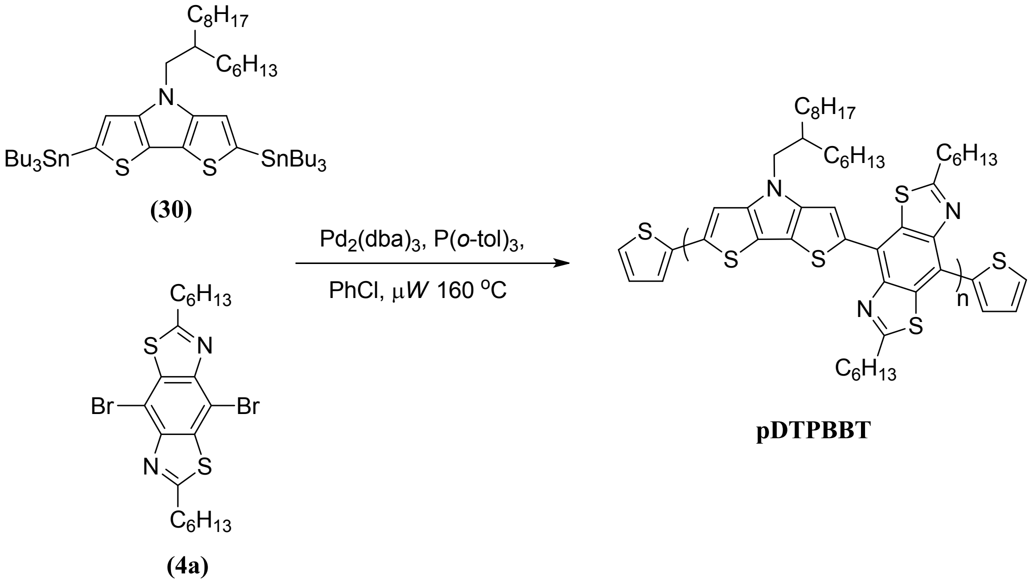

To a 5 mL microwave vial under Ar was added 4,8-Dibromo-2,6-dihexylbenzo[1,2-d:4,5-d']bis(thiazole) **(4a)** (306 mg, 0.590 mmol), 4-(2-Hexyldecyl)-2,6-bis(tributylstannyl)-4H-dithieno[3,2-b:2',3'-d]pyrrole **(30)** (430 mg, 0.590 mmol) and anhydrous chlorobenzene (2 mL) and the vial purged with Ar for 20 min. $Pd_2(dba)_3$ (27 mg, 0.029 mmol) and P(*o*-tol)$_3$ (36 mg, 0.118 mmol) were then added and the vial purged for a further 10 min. The vial was then heated to 160 °C for 50 min under Ar before addition of tributyl(thiophen-2-yl)stannane (0.1 mL) and heating for a further 10 min. 2-Bromothiophene (0.5 mL) was then added and the reaction heated for a final 10 min before being cooled to rt and precipitated into methanol (300 mL). The crude polymer was then purified via Soxhlet extraction with methanol, acetone, hexane and finally collection of the product with chloroform. The chloroform extract was concentrated and reprecipitated into acetone to yield the title compound as a dark purple solid (264 mg); Anal. Calculated for $C_{44}H_{63}N_3S_4$: C, 69.52; H, 8.09; N, 5.53. Found: C, 68.30; H, 7.84; N, 5.19; Due to the poor solubility in chloroform at room temperature accurate determination of molecular weight could not be achieved.

^{1}H NMR spectra of the 4,8-BBT copolymers

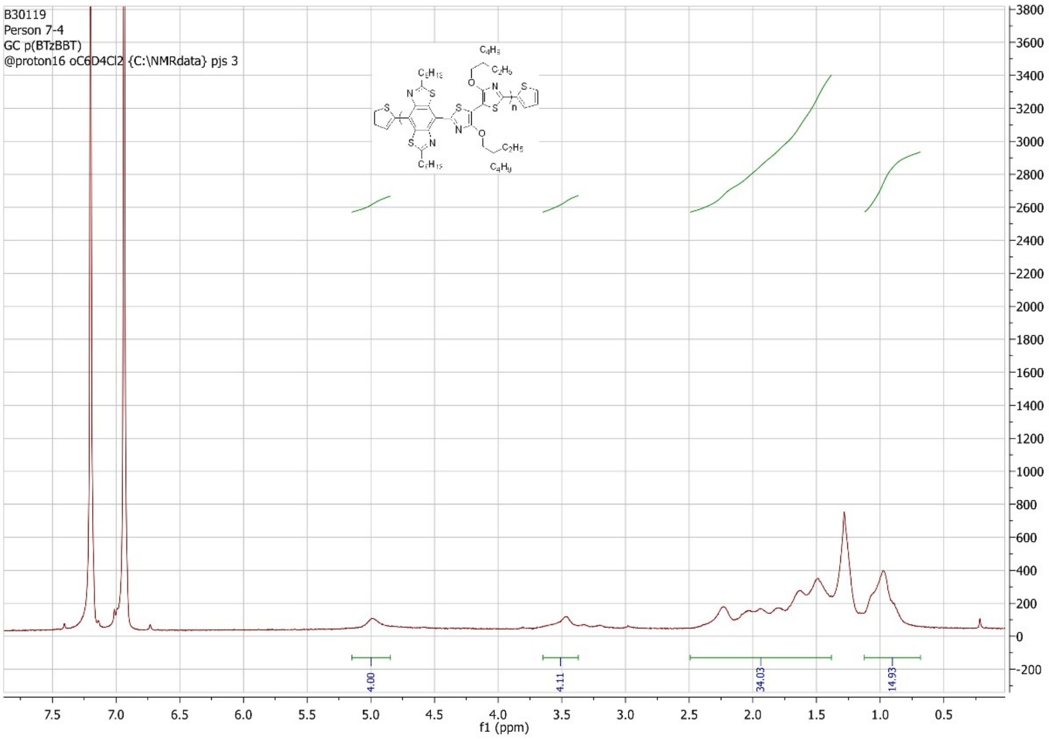

Figure S1: *^{1}H NMR of* ***pBTzBBT*** *in $C_6D_4Cl_2$*

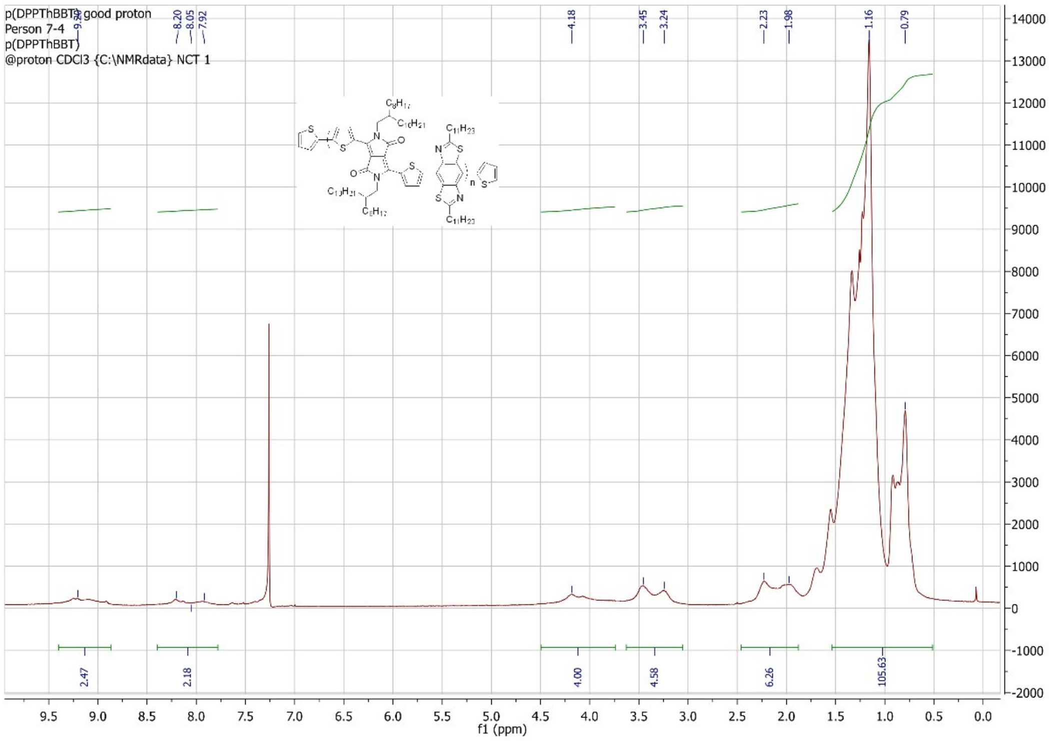

Figure S2: *^{1}H NMR spectra of* ***pDPPThBBT*** *in $CDCl_3$*

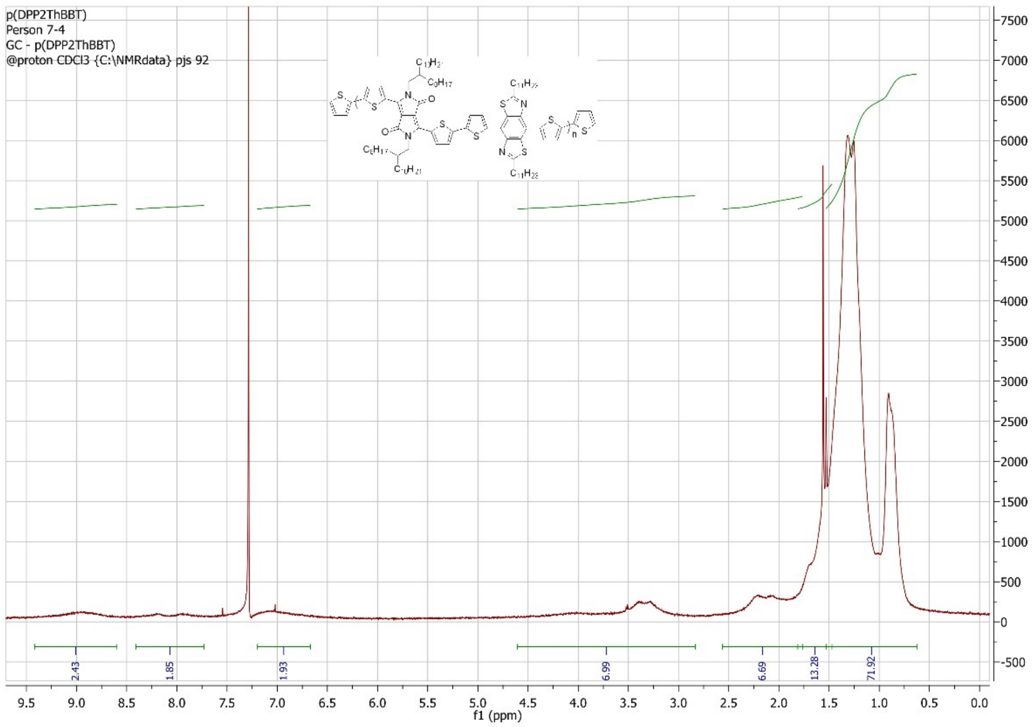

Figure S3: *^{1}H NMR of* ***pDPP2ThBBT*** *in* $CDCl_3$

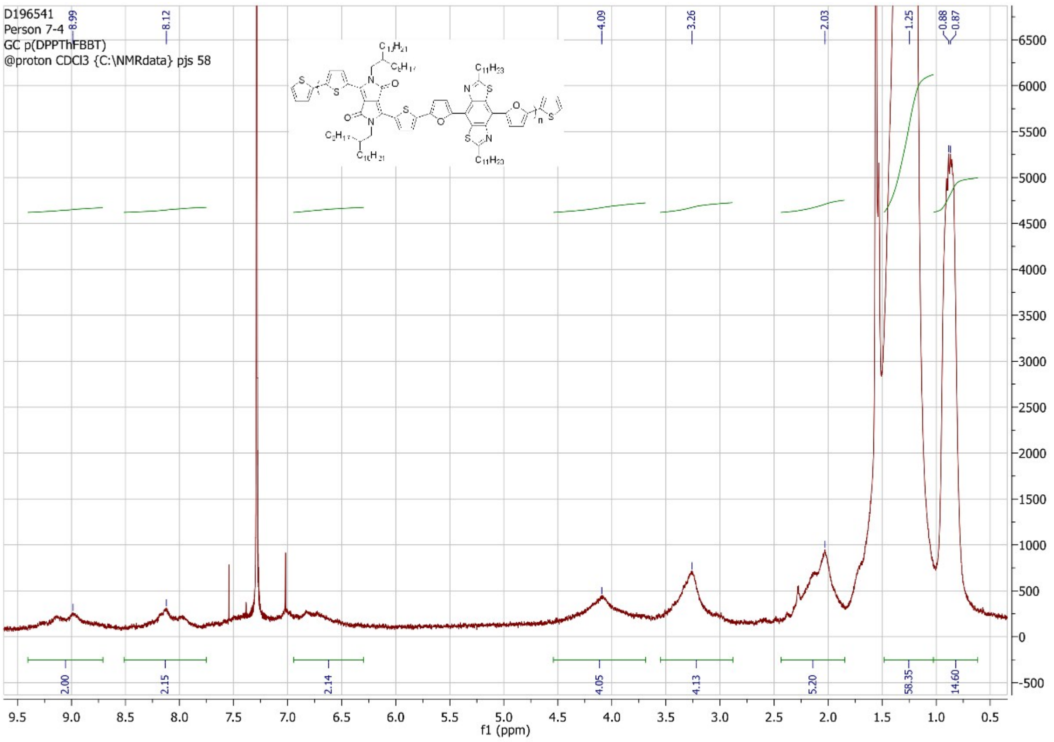

Figure S4: *^{1}H NMR of* ***pDPPThFBBT*** *in* $CDCl_3$

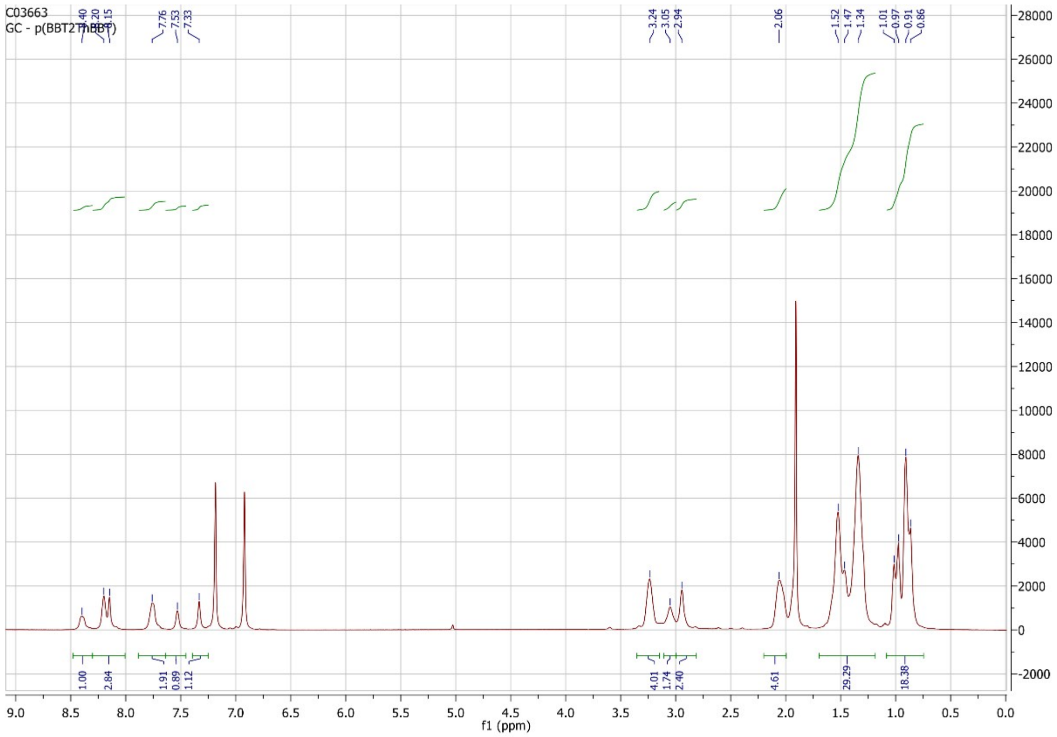

Figure S5: *^{1}H NMR spectrum of **pBT2ThBBT** in* $C_6D_4Cl_2$

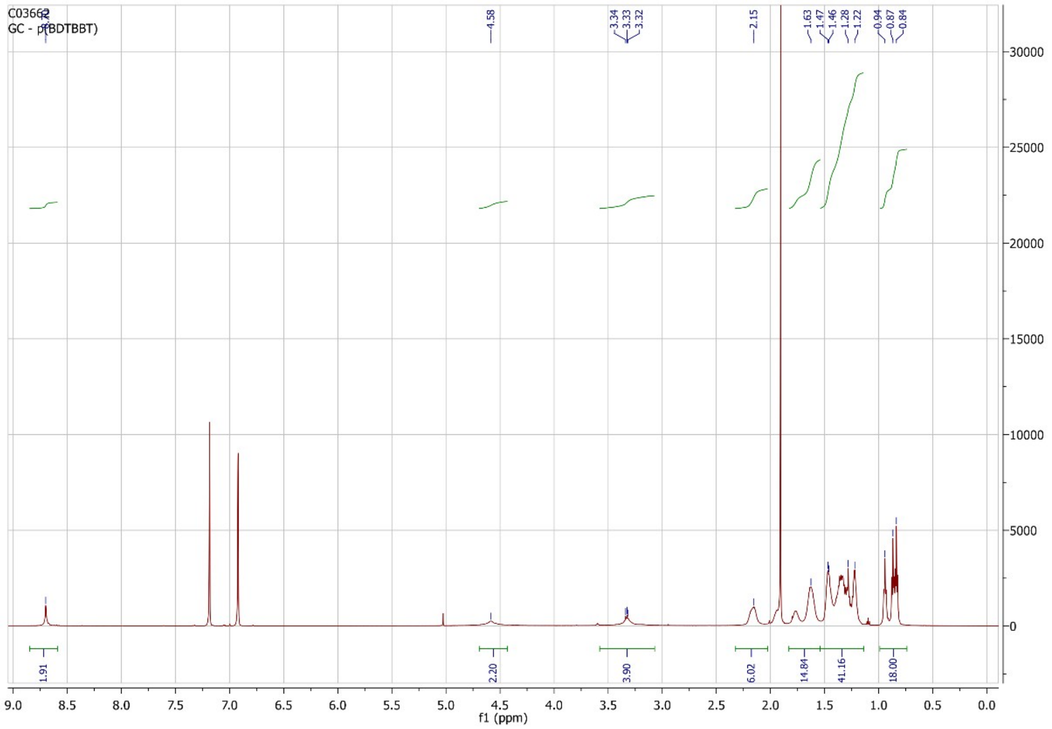

Figure S6: *^{1}H NMR spectrum of **pBDTBBT** in* $C_6D_4Cl_2$

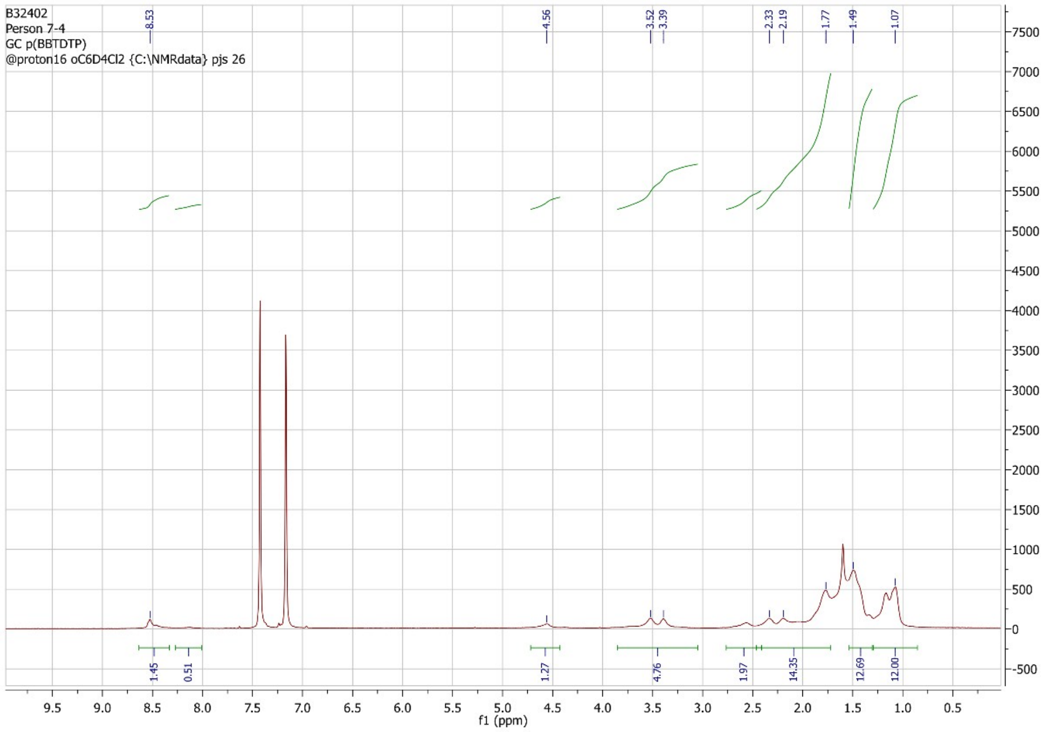

***Figure S7**: ^{1}H NMR of **pDTPBBT** in $C_6D_4Cl_2$*

Cyclic Voltammograms

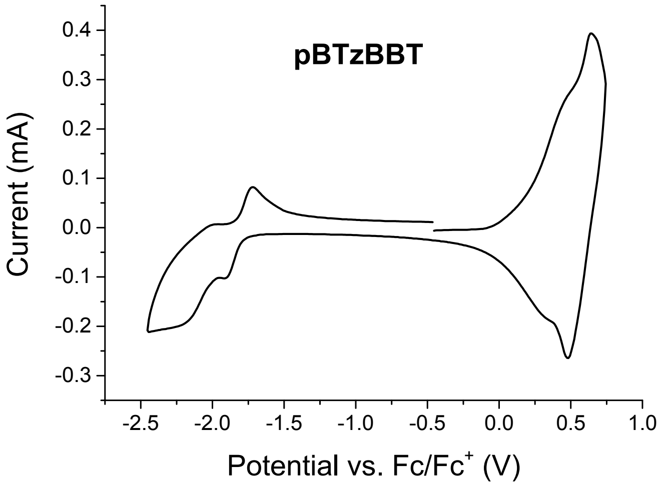

***Figure S8**: Cyclic voltammogram of **pBTzBBT** conducted as a thin-film on platinum disk electrode with MeCN/^{n}Bu$_4$NPF$_6$ supporting electrolyte (100 mV/s scan rate).*

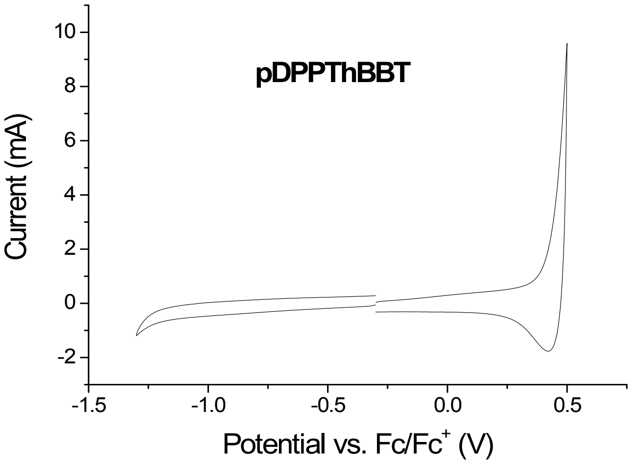

Figure S9*: Cyclic voltammogram of* ***pDPPThBBT*** *conducted as a thin-film on platinum disk electrode with MeCN/$_n$Bu$_4$NPF$_6$ supporting electrolyte (100 mV/s scan rate).*

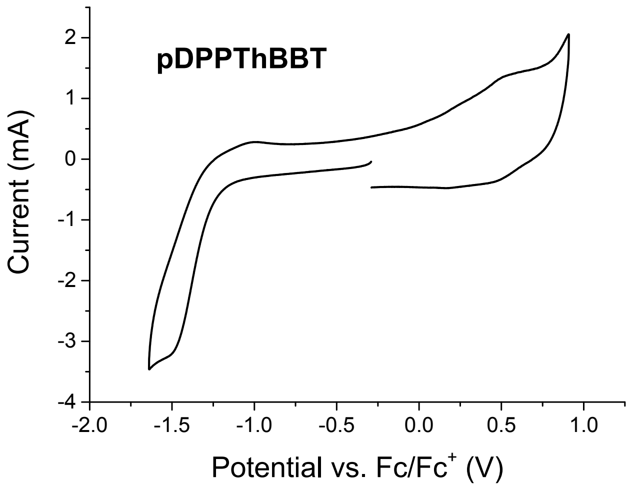

Figure S10*: Cyclic voltammogram of* ***pDPPThBBT*** *conducted using a 0.1 mg/mL solution in CHCl$_3$/^{n}Bu$_4$NPF$_6$ (100 mV/s scan rate).*

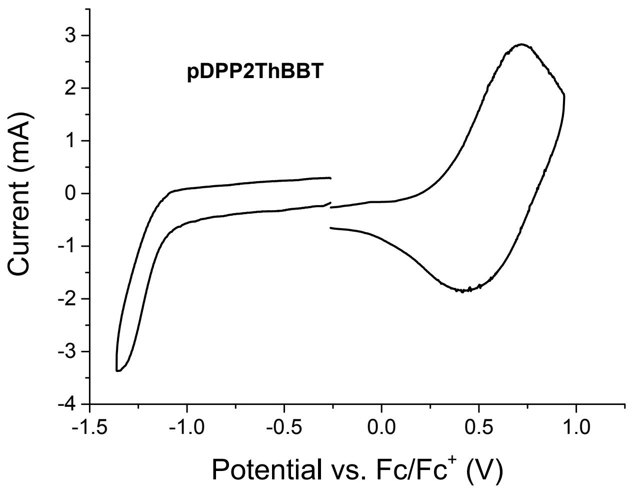

***Figure S11**: Cyclic voltammogram of **pDPP2ThBBT** conducted as a thin-film on platinum disk electrode with MeCN/$^{n}Bu_4NPF_6$ supporting electrolyte (100 mV/s scan rate).*

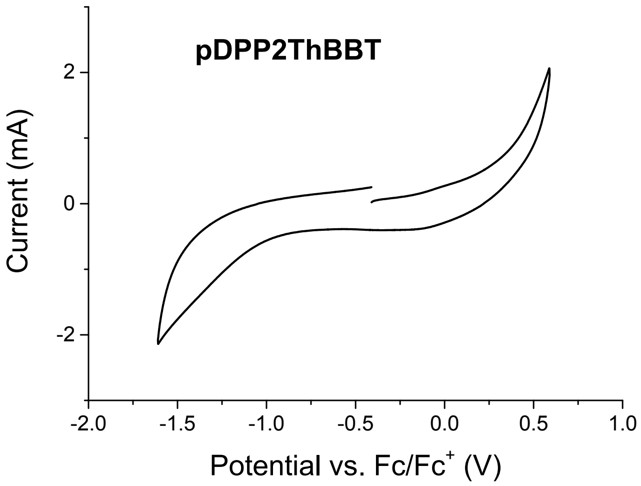

***Figure S12**: Cyclic voltammogram of **pDPP2ThBBT** conducted using a 0.1 mg/mL solution in $CHCl_3$/$^{n}Bu_4NPF_6$ (100 mV/s scan rate).*

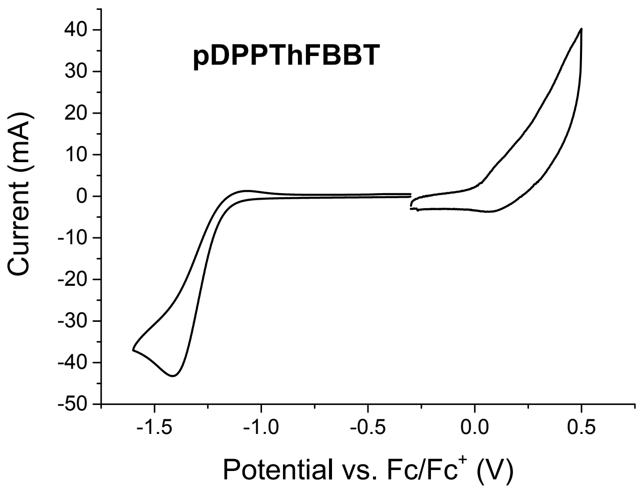

Figure S13: *Cyclic voltammogram of* ***pDPPThFBBT*** *conducted as a thin-film on platinum disk electrode with MeCN/$^{n}Bu_4NPF_6$ supporting electrolyte (100 mV/s scan rate).*

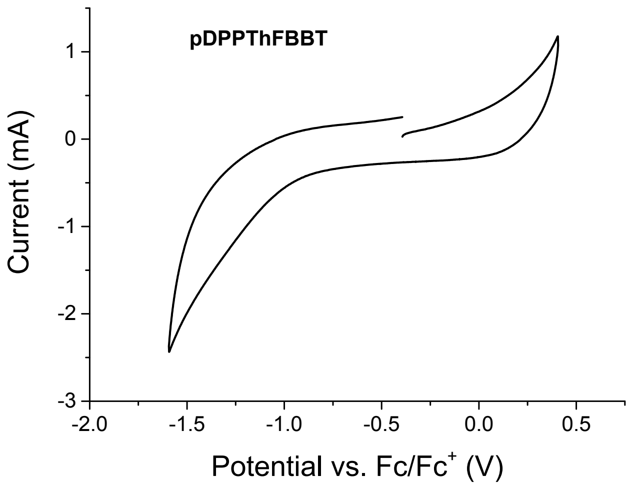

Figure S14: *Cyclic voltammogram of* ***pDPPThFBBT*** *conducted using a 0.1 mg/mL solution in $CHCl_3$/$^{n}Bu_4NPF_6$ (100 mV/s scan rate).*

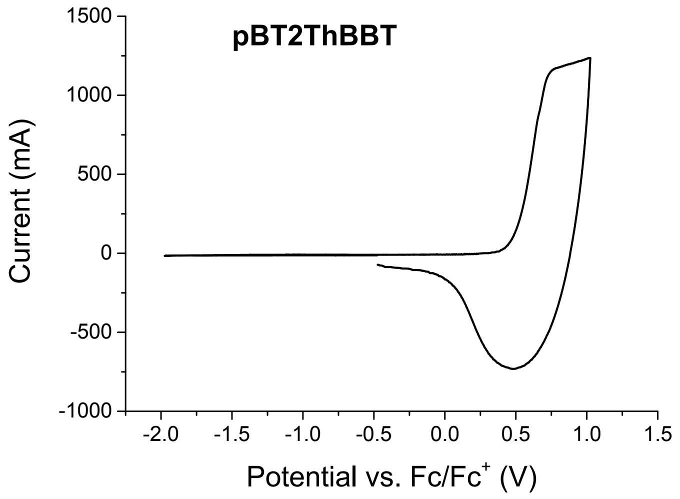

***Figure S15**: Cyclic voltammogram of **pBT2ThBBT** conducted as a thin-film on platinum disk electrode with MeCN/$^{n}Bu_4NPF_6$ supporting electrolyte (100 mV/s scan rate).*

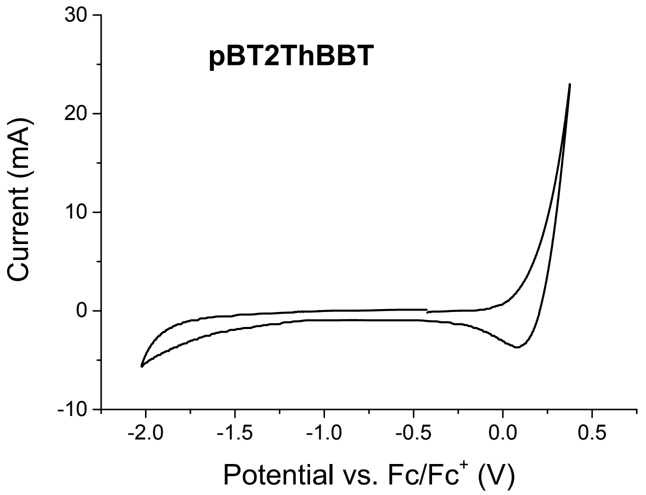

***Figure S16:** Cyclic voltammogram of **pBT2ThBBT** conducted using a 0.1 mg/mL solution in $CHCl_3$/$^{n}Bu_4NPF_6$ (100 mV/s scan rate).*

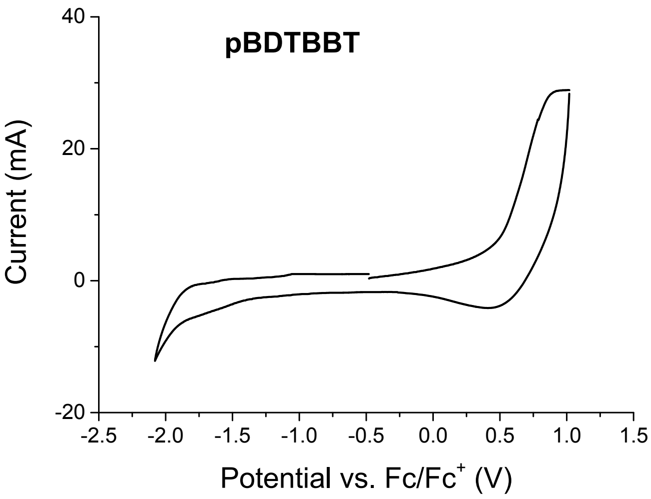

***Figure S17**: Cyclic voltammogram of **pBDTBBT** conducted as a thin-film on platinum disk electrode with MeCN/$^{n}Bu_4NPF_6$ supporting electrolyte (100 mV/s scan rate).*

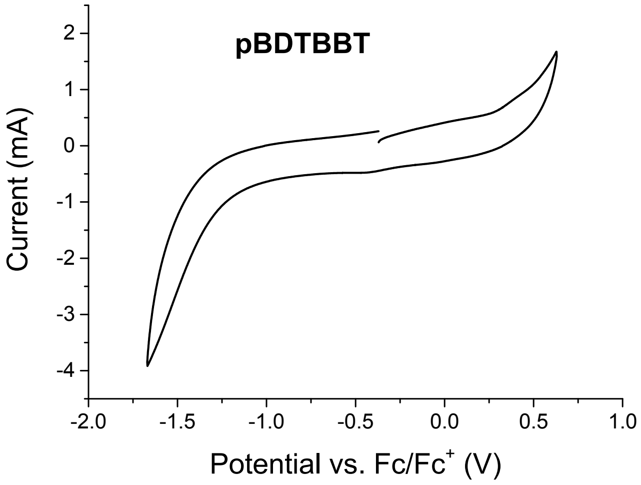

***Figure S18:**: Cyclic voltammogram of **pBDTBBT** conducted using a 0.1 mg/mL solution in $CHCl_3$/$^{n}Bu_4NPF_6$ (100 mV/s scan rate).*

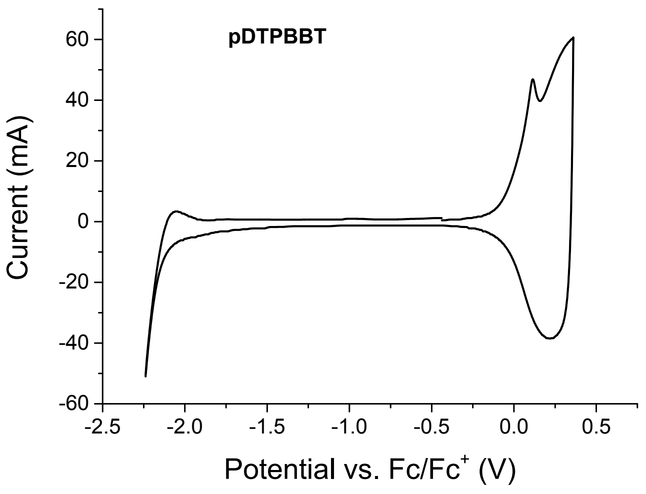

Figure S19: *Cyclic voltammogram of* ***pDTPBBT*** *conducted as a thin-film on platinum disk electrode with* $MeCN/^{n}Bu_4NPF_6$ *supporting electrolyte (100 mV/s scan rate).*

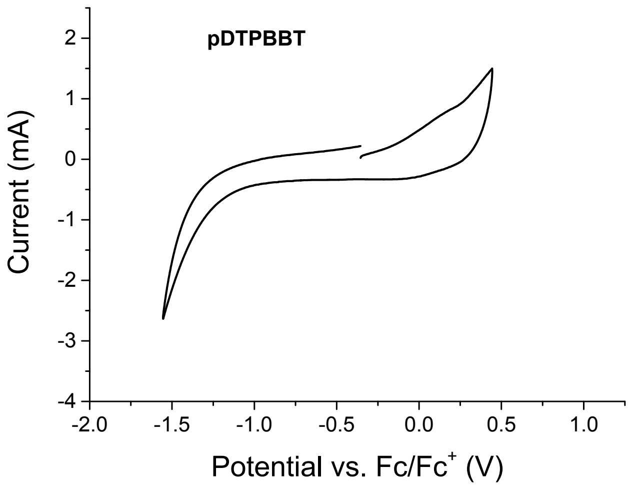

Figure S20*: Cyclic voltammogram of* ***pDTPBBT*** *conducted using a 0.1 mg/mL solution in* $CHCl_3/^{n}Bu_4NPF_6$ *(100 mV/s scan rate).*

Organic Field-Effect Transistor Data

pDPP2ThBBT optimisation study

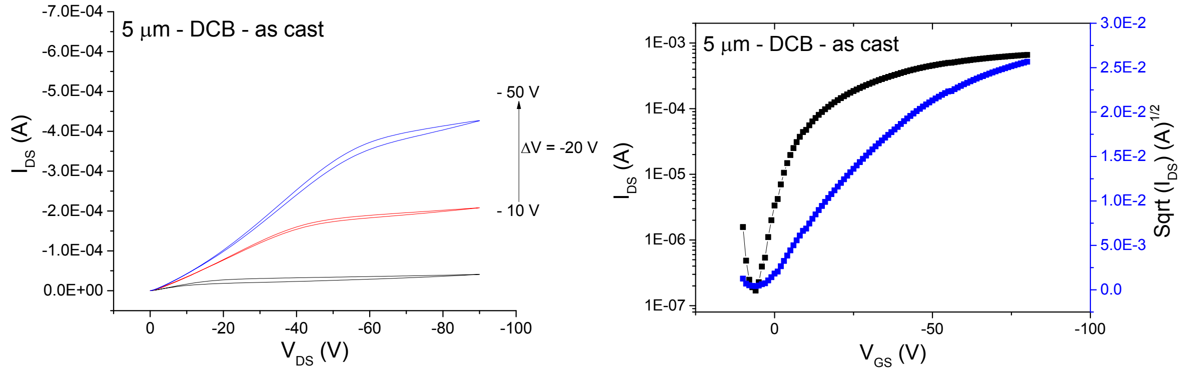

***Figure S21**: Output (left) and Transfer (right) characteristics of a **pDPP2ThBBT** OFET (5 μm/DCB/as cast)*

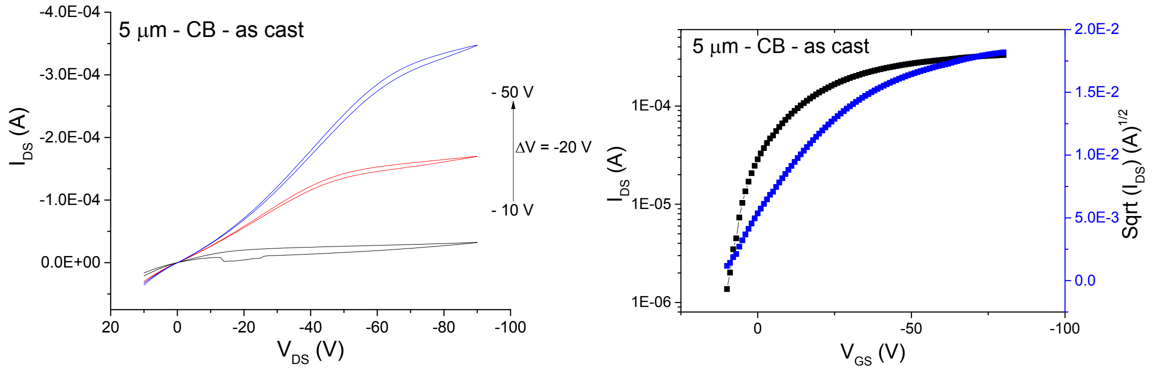

Figure S22**: Output (left) and Transfer (right) characteristics of a **pDPP2ThBB OFET *(5 □m/CB/as cast).*

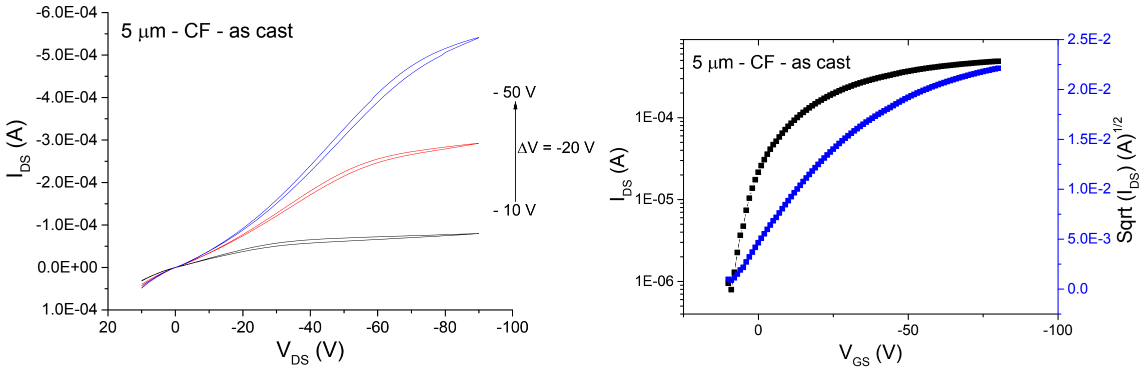

Figure S23**: Output (left) and Transfer (right) characteristics of a **pDPP2ThBBT OFET *(5 □m/CF/as cast).*

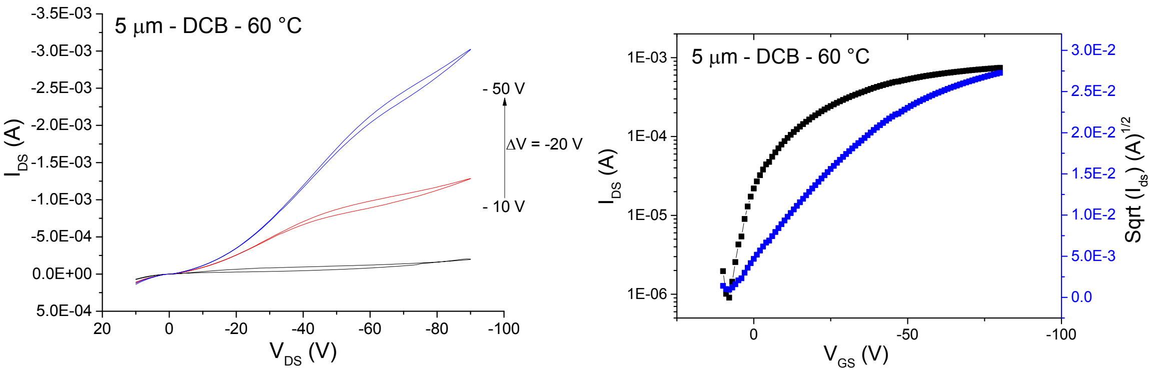

Figure S24: *Output (left) and Transfer (right) characteristics of a* ***pDPP2ThBBT*** OFET *(5 μm/DCB/60 °C).*

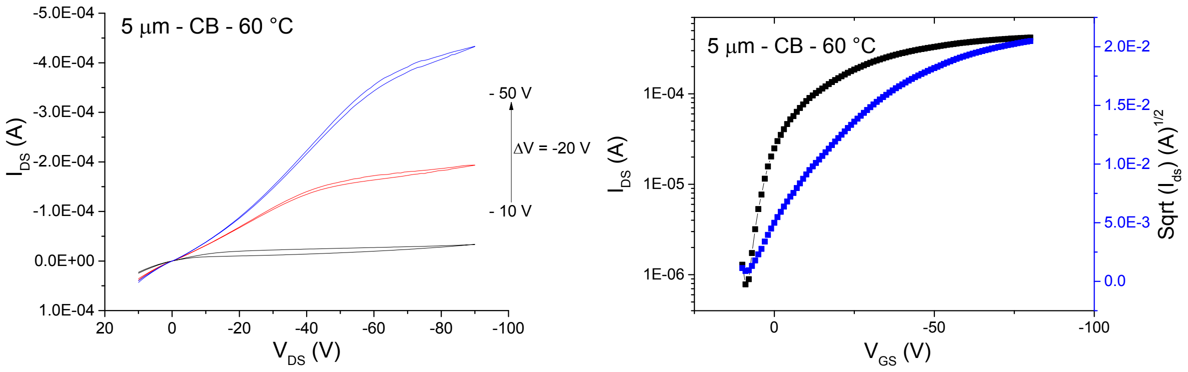

Figure S25: *Output (left) and Transfer (right) characteristics of a* ***pDPP2ThBBT*** OFET *(5 μm/CB/60 °C).*

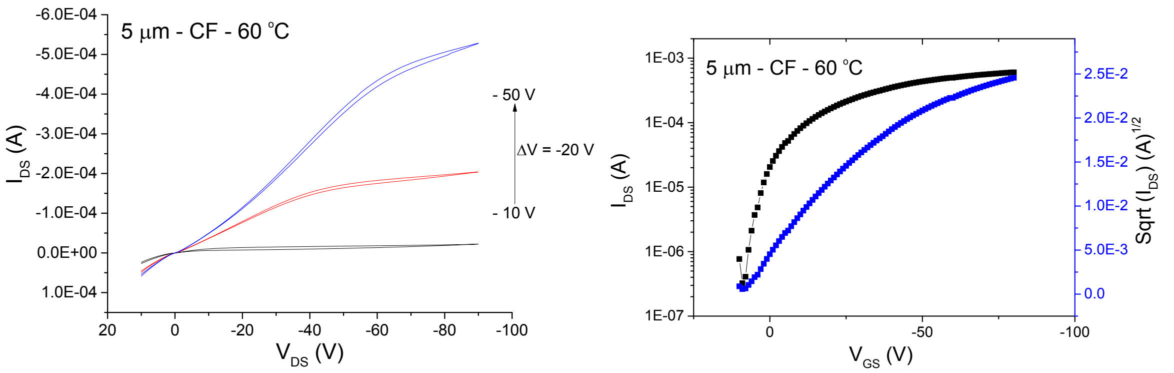

Figure S26: *Output (left) and Transfer (right) characteristics of a* ***pDPP2ThBBT*** OFET *(5 μm/CF/60 °C).*

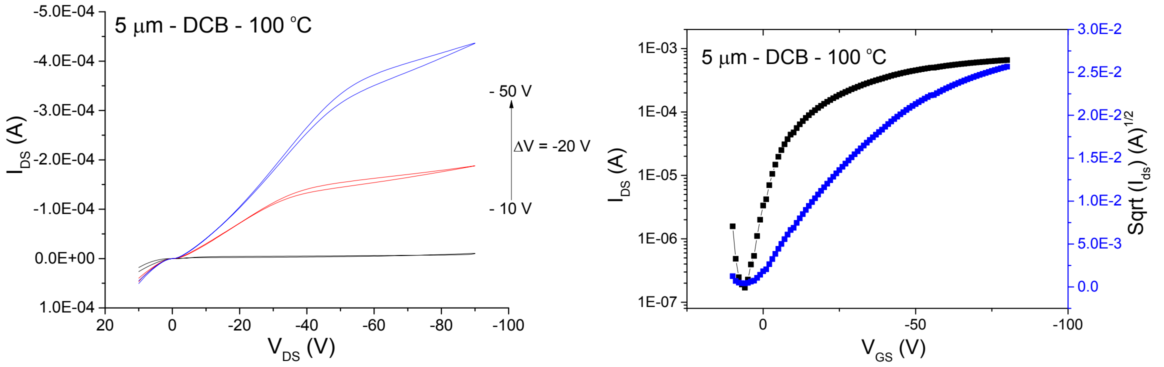

***Figure S27**: Output (left) and Transfer (right) characteristics of a **pDPP2ThBBT** OFET (5 μm/DCB/100 °C).*

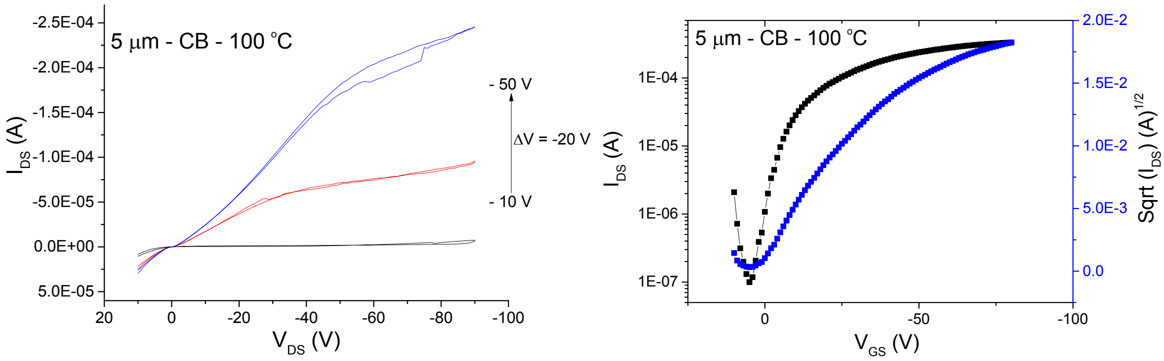

***Figure S28**: Output (left) and Transfer (right) characteristics of a **pDPP2ThBBT** OFET (5 μm/CB/100 °C).*

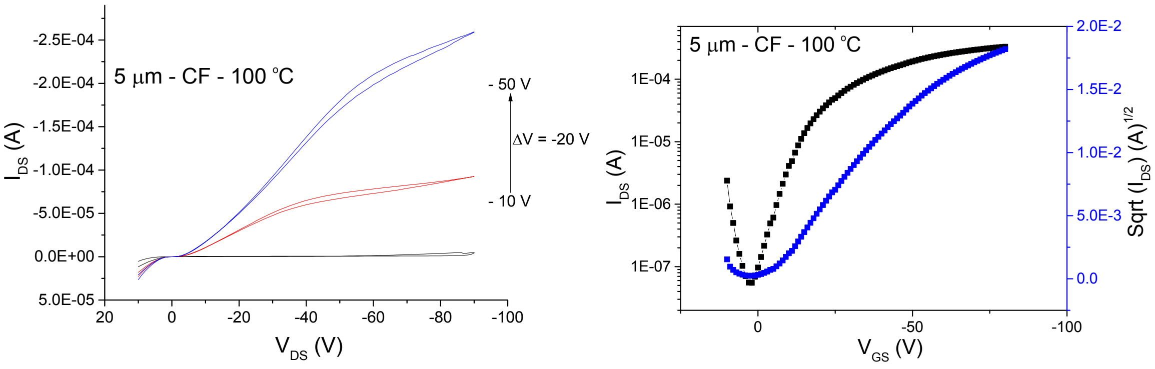

***Figure S29**: Output (left) and Transfer (right) characteristics of a **pDPP2ThBBT** OFET (5 μm/CF/100 °C).*

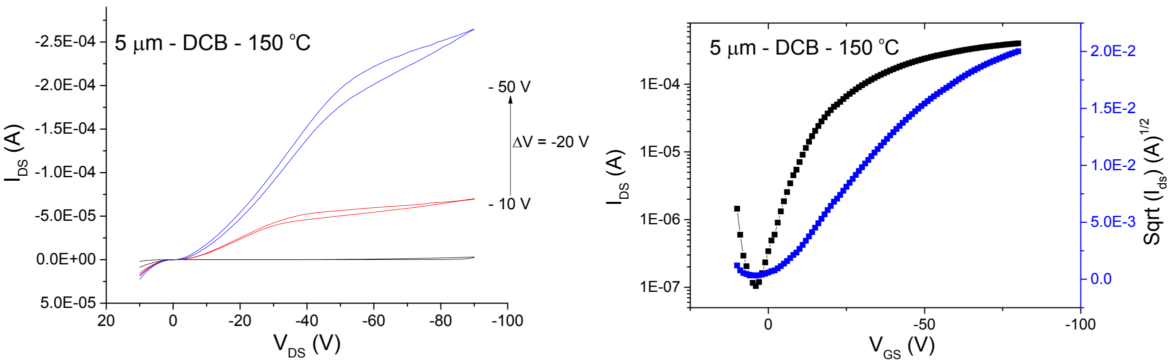

Figure S30**: Output (left) and Transfer (right) characteristics of a **pDPP2ThBBT OFET *(5 □m/DCB/150 °C).*

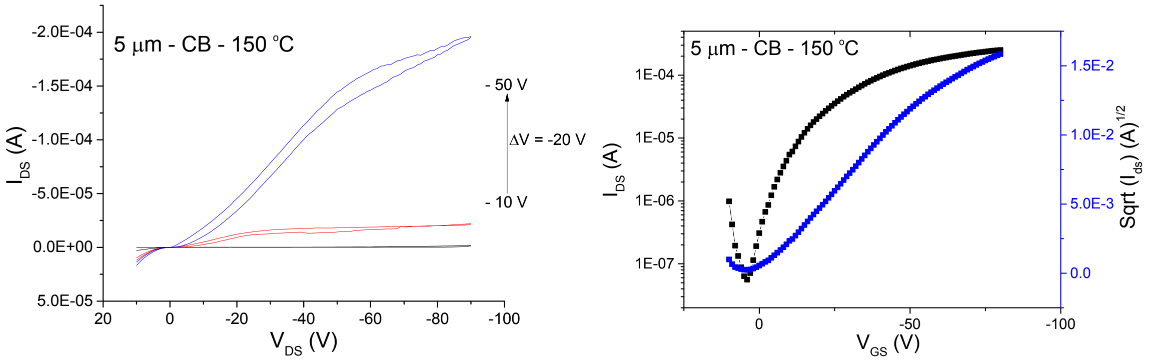

Figure S31**: Output (left) and Transfer (right) characteristics of a **pDPP2ThBBT OFET *(5 □m/CB/150 °C).*

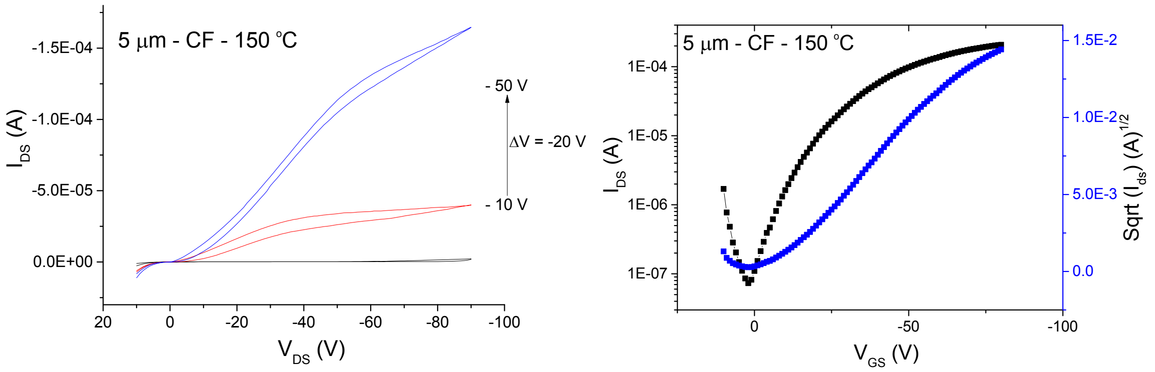

Figure S32**: Output (left) and Transfer (right) characteristics of a **pDPP2ThBBT OFET *(5 □m/CF/150 °C).*

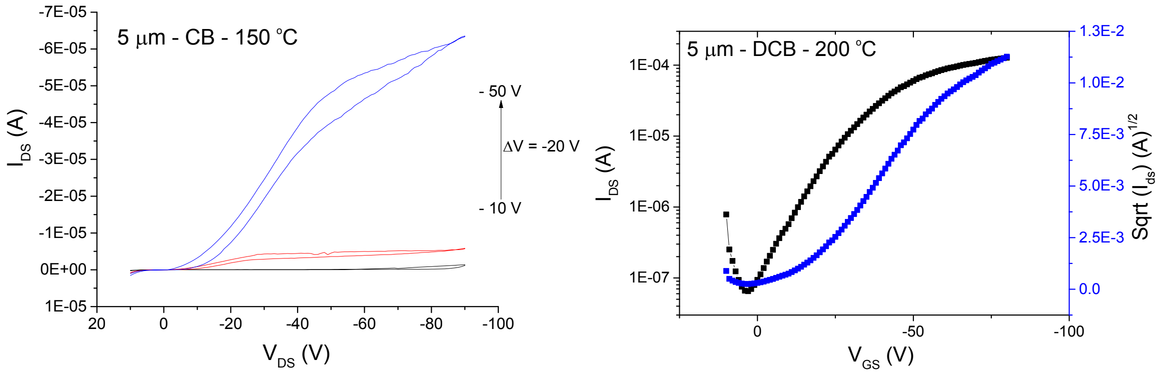

Figure S33**: Output (left) and Transfer (right) characteristics of a **pDPP2ThBBT OFET *(5 □m/DCB/200 °C).*

Other 4,8-BBT copolymers

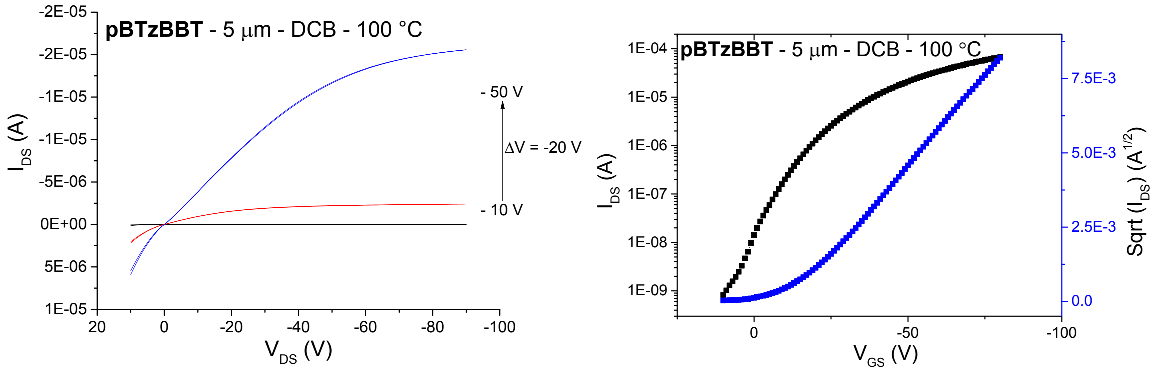

Figure S34**: Output (left) and Transfer (right) characteristics of a **pBTzBBT OFET *(5 □m/DCB/100 °C.*

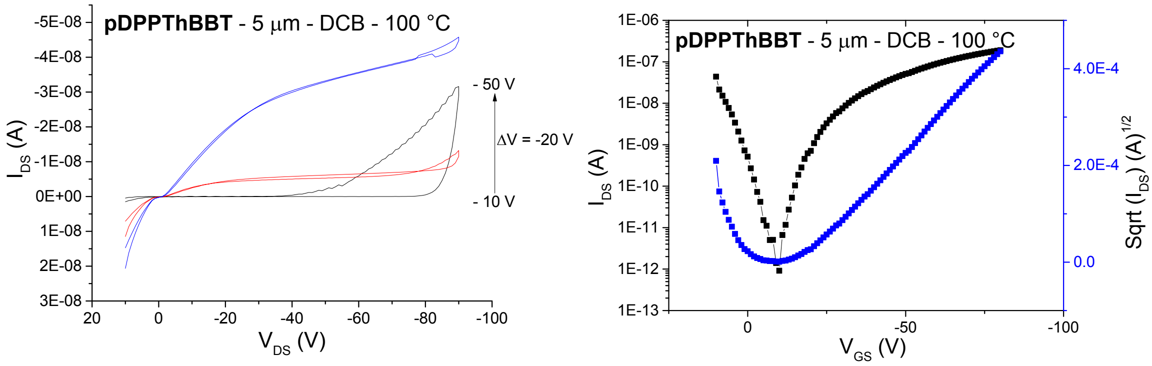

Figure S35**: Output (left) and Transfer (right) characteristics of a **pDPPThBBT OFET *(5 □m/DCB/100 °C).*

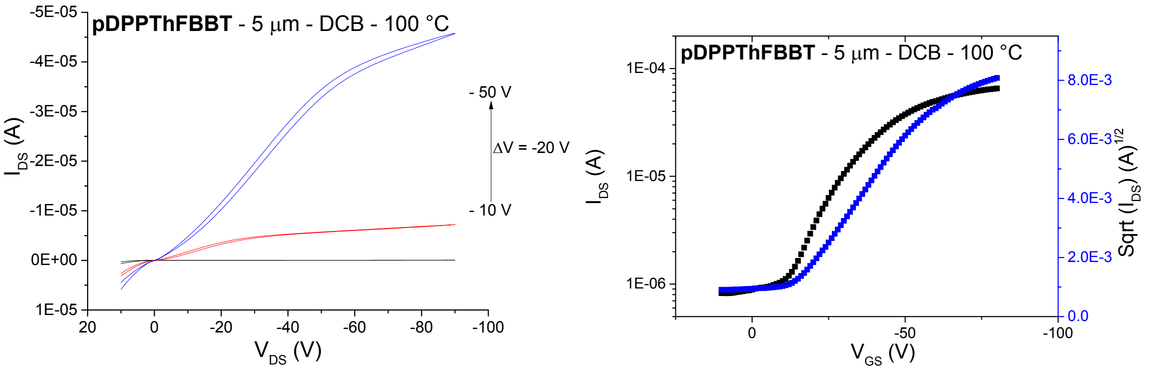

Figure S36**: Output (left) and Transfer (right) characteristics of a **pDPPThFBBT OFET *(5 □m/DCB/100 °C).*

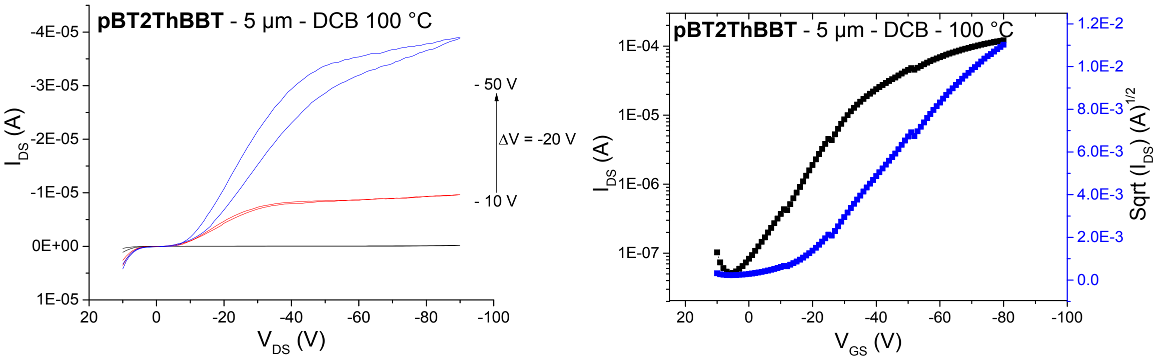

Figure S37**: Output (left) and transfer (right) characteristics of **pBBT2ThBT OFET ***(**5 □m/DCB/100 °C)*

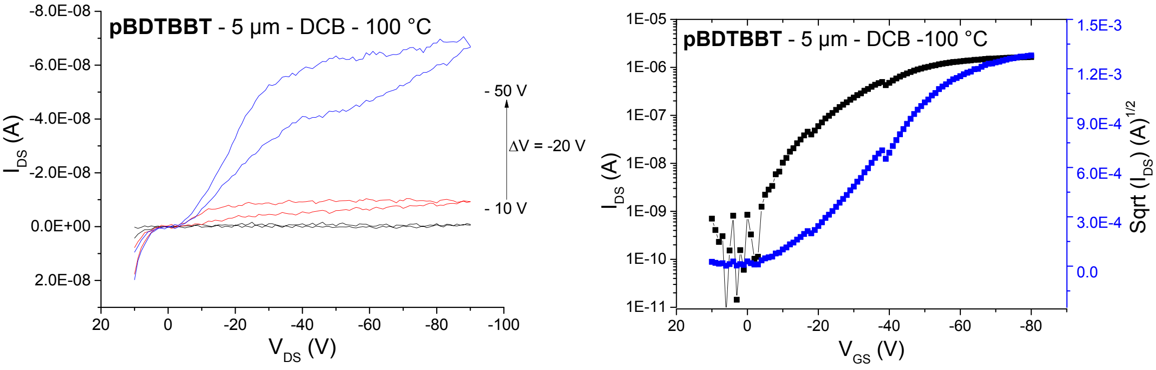

Figure S38**: Output (left) and transfer (right) characteristics of **pBDTBBT OFET *(5 □m/DCB/100 °C).*

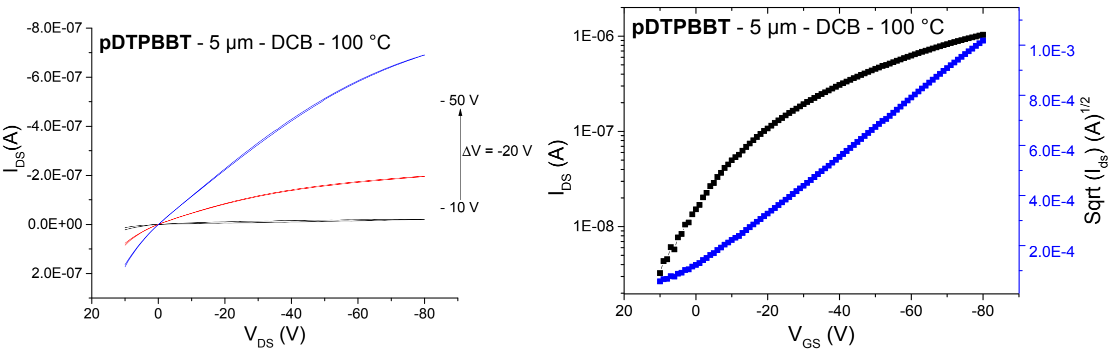

Figure S39**: Output (left) and transfer (right) characteristics of a **pDTPBBT OFET *(5 □m/DCB/100 °C).*

OPV device Optimisation

pDPPThBBT

Solar cells were fabricated in the inverted structure (ITO/Cs_2CO_3/Active layer/MoO_3/Ag). For the inverted structure Cs_2CO_3 was spin-coated on ITO substrates from 2-methoxyethanol solution of 2 mg ml^{-1} concentration at a spin speed of 5000 rpm to yield a film thickness of less than 5 nm. The film was annealed at 150°C for 10 minutes. The layer of MoO_3, which acts as the anode in the inverted structure, was thermally evaporated followed by a metal contact of more than 150 nm of silver. The active layer blend constitutes of a 1:3 donor:acceptor mix (*w/w*) with a total concentration of 70 mg ml^{-1}, using chlorobenzene as the solvent.

**Blend ratio**	**1:1**		**1:3**		**1:5**	
**Concentration (mg/ml)**	69		75		74	
**Thickness (nm)**	91±10		110±15		132±14	
**Architecture**	Inverted	Conventional	Inverted	Conventional	Inverted	Conventional
**PCE avg. (%)**	0.48 ± 0.02	0.04 ± 0.00	0.5 ± 0.03	0.06 ± 0.01	0.41 ± 0.02	0.14 ± 0.01
**PCE best (%)**	0.50	0.4	0.53	0.07	0.43	0.15
**J_{sc} (mA/cm^2)**	2.28 ± 0.05	0.52 ± 0.03	1.60 ± 0.05	0.61 ± 0.02	1.28 ± 0.02	1.03 ± 0.02
**V_{oc} (mV)**	557 ± 18	495 ± 2	622 ± 27	478 ± 14	588 ± 23	498 ± 4

**FF (%)**	37.9 ± 0.4	15.2 ± 0.2	50.2 ± 1	19.2 ± 0.8	54.9 ± 2.0	27.3 ± 0.9

Table S1*: OPV device parameters as a function of blend ratio for* ***pDPPThBBT****. Device averages were obtained over 6 devices.*

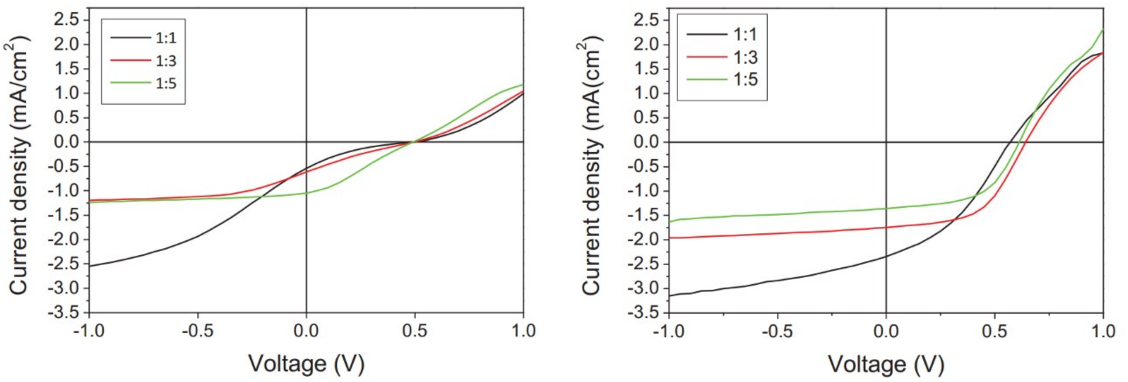

Figure S40: Conventional (left) and inverted (right) solar cell parameters as a function of D:A ratio. Conventional architecture: ITO/MoO_3/pDPPThBBT:$PC_{71}BM$/Ca/Al). Inverted architecture: ITO/Cs_2CO_3/ pDPPThBBT:$PC_{71}BM$ /MoO_3/Ag

pDPP2ThBBT

Device	Ratio	Spin coating speed (rpm)	J_{SC} (mA cm^{-2})	V_{OC} (V)	*FF* (%)	PCE (%) Avg.	PCE(%) best
pDPP2ThBBT:$PC_{71}BM$	0.5:1	700	2.4±0.5	0.6±0.02	60±5	0.9±0.3	1.2
pDPP2ThBBT:$PC_{71}BM$	0.5:1	1000	1.75±0.75	0.6±0.16	64±5	0.6±0.2	0.8

Table S2*: OPV device optimisation for* ***pDPP2ThBBT. Effect of thickness is investigated.*** *Device averages were obtained over 6 devices.*

pDPPThFBBT

Device	Solvent and conditions	J_{SC} (mA cm^{-2})	V_{OC} (V)	*FF* (%)	PCE (%) Avg.	PCE (%) best	DIO
pDPPThFBBT: $PC_{71}BM$	*o*-Dichlorobenzene, annealed at 120°C	2.25±0.45	0.59±0.12	18±3.6	0.20±0.05	0.25	Yes
pDPPThFBBT: $PC_{71}BM$	*o*-Dichlorobenzene, annealed at 120°C	2.50±0.12	0.64±0.03	55.0±1.1	0.90±0.02	0.92	No

Table S3*: OPV device optimisation for* ***pDPPThFBBT. Effect of DIO additive is investigated*** *Device averages were obtained over 6 devices.*

pBDTBBT

Device	Solvent and conditions	J_{SC} (mA cm^{-2})	V_{OC} (V)	*FF* (%)	PCE Avg. (%)	PCE best (%)	DIO (3%)
pBDTBBT: $PC_{71}BM$ (1:1) Bulk	*o*-Dichlorobenzene, 1000 rpm, without annealing	2.71±0.05	0.81±0.02	39±0.78	0.82±0.02	0.84	Yes
pBDTBBT: $PC_{71}BM$ (1:1) Bulk	*o*-Dichlorobenzene, 1000 rpm, annealed at 120°C for 10 mins	1.27±0.03	0.49±0.01	50.0±1.0	0.30±0.01	0.31	Yes
pBDTBBT: $PC_{71}BM$ (1:1) Bulk	*o*-Dichlorobenzene, 3000 rpm, without annealing	2.70±0.05	0.65±0.01	40±0.40	0.69±0.01	0.70	Yes
pBDTBBT: $PC_{71}BM$ (1:1) Bulk	*o*-Dichlorobenzene, 3000 rpm, annealed at 120°C for 10 mins	2.12±0.04	0.80±0.02	45±0.09	0.74±0.01	0.75	Yes
pBDTBBT: $PC_{71}BM$ (1:1) Bulk	*o*-Dichlorobenzene, 1000 rpm, without annealing	1.13±0.02	0.87±0.02	33.0±0.7	0.31±0.01	0.32	No
pBDTBBT: $PC_{71}BM$ (1:1) Bulk	*o*-Dichlorobenzene, 1000 rpm, annealed at 120°C for 10 mins	2.26±0.04	0.83±0.02	38.0±0.8	0.69±0.02	0.71	No
pBDTBBT: $PC_{71}BM$ (1:1) Bulk	*o*-Dichlorobenzene, 3000 rpm, without annealing	1.03±0.02	0.94±0.02	29.0±0.6	0.29±0.01	0.30	No
pBDTBBT: $PC_{71}BM$ (1:1) Bulk	*o*-Dichlorobenzene, 3000 rpm, annealed at 120°C for 10 mins	2.00±0.04	0.80±0.02	43.0±0.09	0.69±0.01	0.70	No
	Concentration of solution: 30 mg ml^{-1}<br>Device layout: ITO/PEDOT:PSS/Active layer/Ca/Al						

Table S4*: OPV device optimisation for* ***pBDTBBT. Effect of DIO additive, annealing temperature, and spin coating speed were investigated.*** *Device averages were obtained over 6 devices.*

pDTPBBT

Device	Solvent and conditions	J_{SC} (mA cm^{-2})	V_{OC} (V)	*FF* (%)	PCE Avg. (%)	PCE (%) best	DIO (3%)
pDTPBBT: $PC_{71}BM$ (1:0.8)	Chlorobenzene, 25 mg/ml, 800 rpm, annealed at 100°C, 10mins	0.93±0.01	0.51±0.01	26.5±0.2	0.12±0.01	0.13	No
pDTPBBT: $PC_{71}BM$ (1:0.8)	Chlorobenzene, 25 mg/ml, 800 rpm, annealed at 150°C, 10mins	2.80±0.06	0.69±0.19	30.8±7.7	0.57±0.04	0.62	No
pDTPBBT: $PC_{61}BM$ (1:1)	Chlorobenzene, 20 mg/ml, 800 rpm, RT	0.41±0.04	0.85±0.02	28±0.32	0.10±0.01	0.11	No
pDTPBBT: $PC_{71}BM$ (1:1)	Chlorobenzene, 20 mg/ml, 800 rpm, RT	0.75±0.09	0.89±0.01	28±0.57	0.19±0.02	0.22	No
pDTPBBT: $PC_{71}BM$ (1:1)	Chlorobenzene, 20 mg/ml, 800 rpm, annealed at 100°C, 10mins	1.55±0.08	0.83±0.03	33.6±0.16	0.43±0.02	0.45	No
pDTPBBT: $PC_{71}BM$ (1:1.25)	Chlorobenzene, 25 mg/ml, 800 rpm, RT	1.59±0.30	0.46±0.04	29.4±3.4	0.22±0.06	0.27	Yes
pDTPBBT: $PC_{71}BM$ (1:1.25)	Chlorobenzene, 25 mg/ml, 800 rpm, annealed at 100°C, 10mins	1.90±0.11	0.51±0.01	30.5±0.28	0.29±0.02	0.31	Yes
Concentration of solution: 20-25 mg ml^{-1}<br>Device layout: ITO/PEDOT:PSS/Active layer/Ca/Al. Device averages were obtained over 4-6 devices.							

Table S5**: OPV device optimisation for **pDTPBBT. Effect of donor: acceptor ratio, thermal annealing and DIO additive is investigated.

Supplementary OPV figures

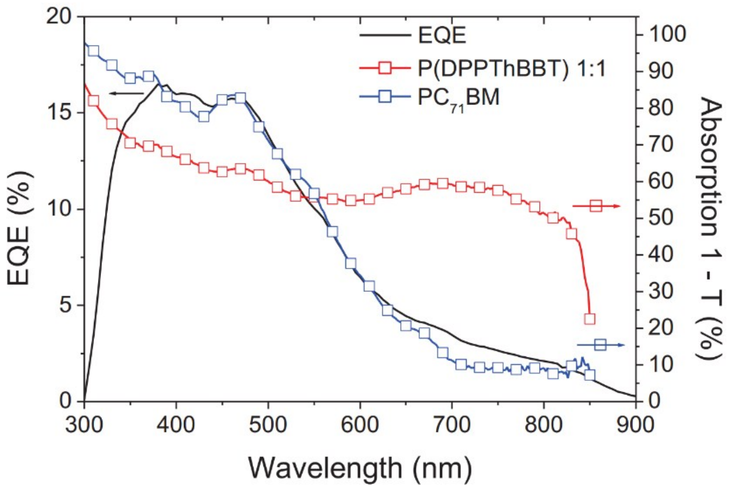

*Figure S41: EQE spectrum of **pDPPThBBT**:$PC_{71}BM$ (black), absorption spectra of blend film (red) and absorption spectra of $PC_{71}BM$ film (blue).*

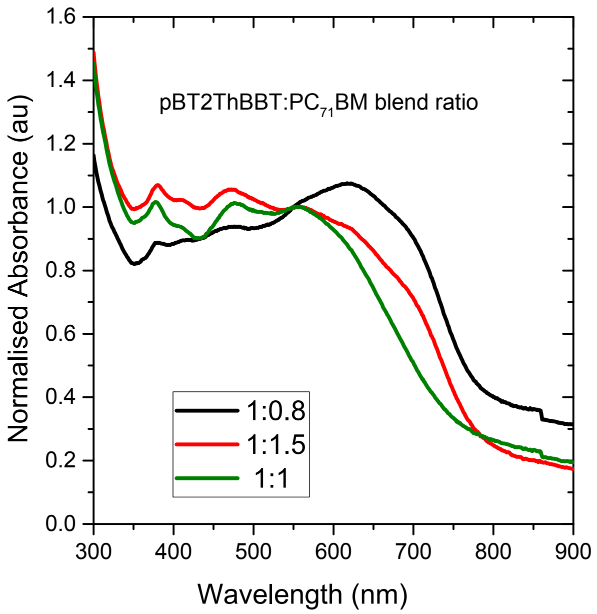

Figure S42: UV-Visible absorption of pBT2ThBBT:PC_{71}BM blend films, normalised to the absorbance at 550 nm.

pBT2ThBBT:PC_{71}BM. Effect of donor: acceptor blend ratio is investigated. Device averages were obtained over 4 devices.

**Device**	**D:A ratio**	***J_{SC}* (mA cm^{-2})**	***V_{OC}* (V)**	***FF* (%)**	**PCE (%) Avg.**	**PCE (%) best**	**DIO**
pDPPThFBBT: PC_{71}BM	1.0:0.8	7.39±0.44	0.67±0.04	53.0±3.8	2.50±0.15	2.64	Yes
pDPPThFBBT: PC_{71}BM	1.0:1.0	14.32±0.39	0.65±0.02	48.0±1.3	4.33±0.12	4.45	Yes
pDPPThFBBT: PC_{71}BM	1.0:1.5	7.72±0.24	0.68±0.02	48.0±1.4	2.18±0.06	2.25	Yes

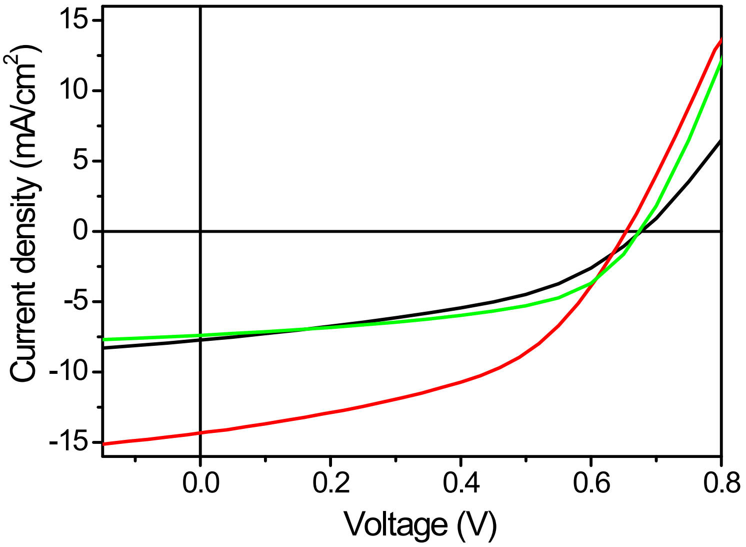

Figure S43: J-V parameters for various blend ratios. Device architecture – ITO/PEDOT:PSS/ pBT2ThBBT:$PC_{71}BM$ blend/Ca/Al.

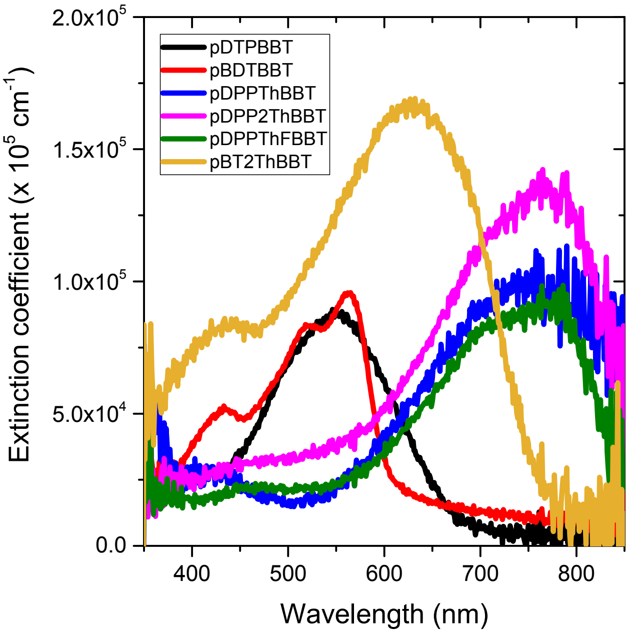

Figure S45: The extinction coefficient of all the BBT polymers in thin film form.

The measurement of exciton diffusion in PBBT2THBT

We have measured time-resolved fluorescence in PBBT2THBT by dispersing the different concentration of $PC_{71}BM$ quencher molecules. The time-resolved florescence was measured by exciting the sample with 100 fs light pulses at 400 nm and PL was detected at 780 nm using a synchroscan streak camera C6860 from Hamamatsu which has time resolution of ~2 ps. The time resolved fluorescence as a function of different concentration of C_{70} quencher is shown in Figure S46.

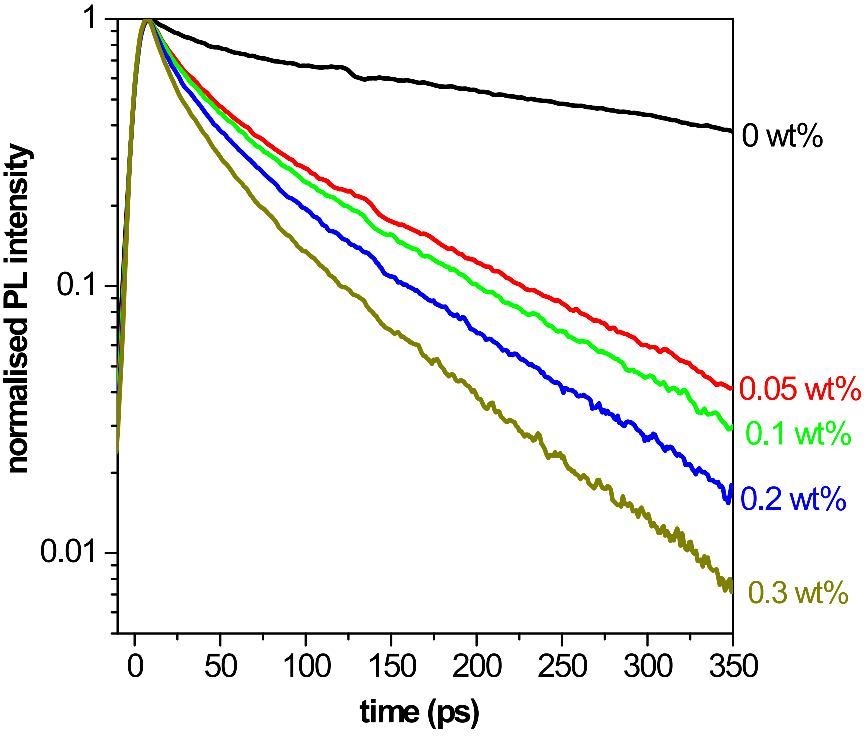

Figure S46: The naturalised PL decays of blends with different concentration of $PC_{71}BM$ quencher.

In order to get quantitative information about exciton diffusion coefficient, we analyse the data in the regime of lower concentration of quencher[1,2] so that excitons will diffuse some distance in order to be quenched by the acceptor. In this regime, the rate of quenching is limited by both diffusion and Förster process i.e. excitons have to diffuse to be within a certain radius of the quencher where rate of quenching is purely Forster-like[3].

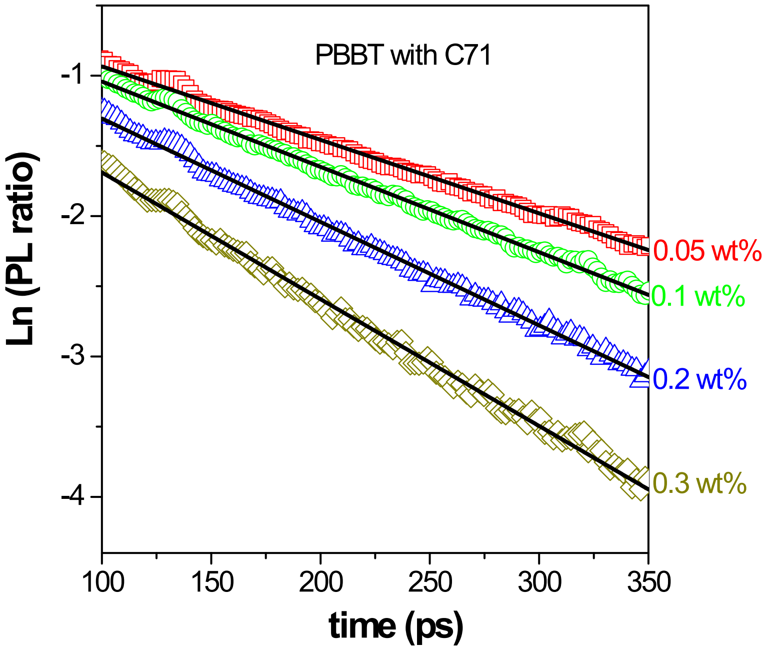

Figure S47: The natural logarithms of the ratio of the PL with lower concentration of quencher. A line was fitted to experimental data to obtain the value of rate of quenching.

The diffusion coefficient could then be generated from the interpolation formula described by Goselle *et a*[4,5];

$$\ln(ratio) = -4\pi D r_F N t - 8 r_F^2 N[\pi D t]^{1/2} \qquad (1)$$

where r_F is the quenching radius

$therefore\ as\ t \to \infty$, the equation 1 becomes

$$\frac{d\ln(ratio)}{dt} = -4\pi r_F D N \qquad (2)$$

and rate of quenching $k_q(t)$ is given by

$$k_q(t) = -\frac{d\ln(ratio)}{dt} \qquad (3)$$

The above equations (2 and 3) show that at lower concentrations of quencher and at long times, the rate of quenching is expected to be time independent. We determine this rate of quenching experimentally by taking the gradient of the ratio of quenched to unquenched films

after 100 ps when the log(ratio)s become linear (fig 2). The obtained values of the rate of quenching are plotted in Fig 3 against concentration of quencher.

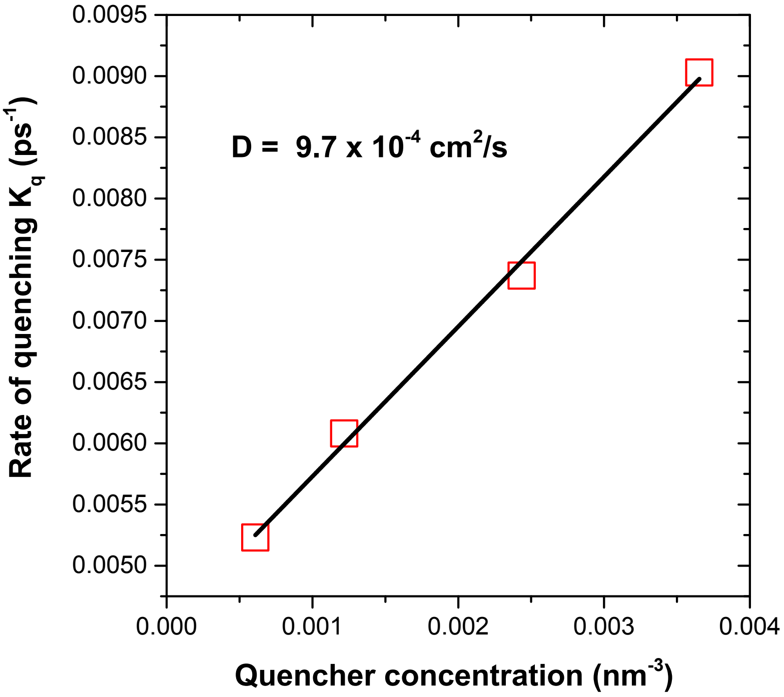

Figure S48: The rate of quenching $k_q(t)$, obtained form by taking the gradient of figure 3 after 100 ps, plotted as a function of concentartion of the quencher. The black is linear fit, used to determine diffusion coeffiecient.

The value of the diffusion coefficient was then extracted by fitting a line to the data and using equations 2 and 3. We found a diffusion coefficient, $D\ of\ (9.7 \pm 0.8) \times 10^{-4}\ cm^2/s$ assuming $r_F = 1\ nm$. The 1D diffusion length is given by $L_{1D} = \sqrt{2D\tau}$ and is found to be $L_{1D} = 10.3\ nm$

References

1. J. F. Wolfe, B. H. Loo and F. E. Arnold, *Macromolecules*, 1981, **14**, 915-920.
2. J. K. Landquist, *J. Chem. Soc. C*, 1967, **1**, 2212-2220.
3. G. Conboy, H. J. Spencer, E. Angioni, A. L. Kanibolotsky, N. J. Findlay, S. J. Coles, C. Wilson, M. B. Pitak, C. Risko, V. Coropceanu, J.-L. Bredas and P. J. Skabara, *Mater. Horiz*, 2016, **3**, 333-339.
4. A. M. Grubb, M. J. Schmidt, A. J. Seed and P. Sampson, *Synthesis*, 2012, **44**, 1026-1029.
5. P. Stanetty, M. Schnürch and M. D. Mihovilovic, *J. Org. Chem.*, 2006, **71**, 3754-3761.
6. X. Guo, J. Quinn, Z. Chen, H. Usta, Y. Zheng, Y. Xia, J. W. Hennek, R. P. Ortiz, T. J. Marks and A. Facchetti, *J. Am. Chem. Soc.*, 2013, **135**, 1986-1996.
7. Y. Li, S. P. Singh and P. Sonar, *Adv. Mater.*, 2010, **22**, 4862-4866.
8. D. Cortizo-Lacalle, S. Arumugam, S. E. T. Elmasly, A. L. Kanibolotsky, N. J. Findlay, A. R. Inigo and P. J. Skabara, *J. Mater. Chem.*, 2012, **22**, 11310-11315.
9. D. E. Seitz, S.-H. Lee, R. N. Hanson and J. C. Bottaro, *Synth. Commun.*, 1983, **13**, 121-128.
10. L. Biniek, S. Fall, C. L. Chochos, D. V. Anokhin, D. A. Ivanov, N. Leclerc, P. Lévêque and T. Heiser, *Macromolecules*, 2010, **43**, 9779-9786.
11. N. Wang, Z. Chen, W. Wei and Z. Jiang, *J. Am. Chem. Soc.*, 2013, **135**, 17060-17068.
12. X. Guo, R. P. Ortiz, Y. Zheng, M.-G. Kim, S. Zhang, Y. Hu, G. Lu, A. Facchetti and T. J. Marks, *J. Am. Chem. Soc.*, 2011, **133**, 13685-13697.
13. Y. A. Getmanenko, T. A. Purcell, D. K. Hwang, B. Kippelen and S. R. Marder, *J. Org. Chem.*, 2012, **77**, 10931-10937.

1. Sajjad, M. T; Ward, A. J.; Kästner, C.; Ruseckas, A.; Hoppe, H.; Samuel, I. D. W., controlling exciton diffusion and fullerene distribution in photovoltaic blends by side chian modification. **Submitted**
2. Ward, A. J.; Ruseckas, A.; Samuel, I. D. W., A Shift from Diffusion Assisted to Energy Transfer Controlled Fluorescence Quenching in Polymer–Fullerene Photovoltaic Blends. *The Journal of Physical Chemistry C* **2012,** *116* (45), 23931-23937.
3. Powell, R. C.; Kepler, R. G., Evidence of Long-Range Exciton-Impurity Interaction in Tetracene-Doped Anthracene Crystals *Physics review letters* **1969**,22,636-639
4. Klein, U. K. A.; Frey, R.; Hauser, M.; Gösele, U., Theoretical and experimental investigations of combined diffusion and long-range energy transfer. *Chemical Physics Letters* **1976**, 41, 139-142.
5. Gösele, U; Hauser, M.; Klein, U. K. A.; Frey, R., Diffusion and long-range energy transfer. *Chemical Physics Letters* **1975**, 34, 519-522.
